# Nutritional status of school-age children and adolescents in low- and middle-income countries across seven global regions: a synthesis of scoping reviews

**DOI:** 10.1017/S1368980022000350

**Published:** 2022-02-14

**Authors:** Stephanie V Wrottesley, Emily Mates, Eilise Brennan, Vasundhara Bijalwan, Rachael Menezes, Stephanie Ray, Zakari Ali, Amirhossein Yarparvar, Deepika Sharma, Natasha Lelijveld

**Affiliations:** 1 Emergency Nutrition Network (ENN), 2nd Floor, 69 High St, Marlborough House, Kidlington, Oxfordshire OX5 2DN, UK; 2 Department of Population Health, London School of Hygiene & Tropical Medicine, London, UK; 3 Nutrition Theme, MRC Unit The Gambia, London School of Hygiene & Tropical Medicine, Banjul, The Gambia; 4 UNICEF Regional Office for Europe and Central Asia, Almaty, Kazakhstan; 5 UNICEF Headquarters, New York, NY, USA

**Keywords:** Nutrition, School-age children, Adolescents, Low- and middle-income countries, Interventions

## Abstract

**Objective:**

To summarise available evidence on the nutritional status of school-age children and adolescents (5–19 years) from seven global regions and on interventions implemented to improve malnutrition in this population.

**Setting:**

Global.

**Design:**

Findings were compiled from seven scoping literature reviews, including data from low- and middle-income countries within the following UNICEF-defined global regions: East Asia and Pacific; Europe and Central Asia; South Asia; West and Central Africa; Eastern and Southern Africa; Middle East and North Africa and Latin America and the Caribbean.

**Results:**

A double burden of malnutrition was evident across the world regions reviewed: stunting, thinness, anaemia and other micronutrient deficiencies persisted, alongside rising overweight and obesity prevalence. Transitions towards diets increasingly high in energy-dense, processed and micronutrient-poor foods were observed. Evidence from intervention studies was limited, but suggested that providing multiple micronutrient-fortified foods or beverages at school may effectively target micronutrient deficiencies and facilitate weight gain in undernourished populations. Interventions to prevent or manage overweight and obesity were even more limited. There was minimal evidence of using novel technological approaches to engage school-age children and adolescents, or of involving them in designing interventions.

**Conclusion:**

The limited data available on nutrition of school-age children and adolescents are neither standardised nor comparable. Consensus on methods for assessing nutritional status and its determinants for this age group is urgently needed to set targets and monitor progress. Additionally, strategies are required to ensure that nutritious, safe and sustainable diets are available, affordable and appealing.

The United Nations Sustainable Development Goal 2 calls for an end to all forms of malnutrition by 2030^(1)^. To this end, global investment in nutrition has predominantly focused on children under 5 years of age and on pregnant and lactating women. In recent years, the adolescent period (defined by the WHO as between the ages of 10 and 19 years) has gained recognition as a potential second window of opportunity for achieving targeted gains in growth and development, with long-term nutrition and health benefits for current and future generations^(2–5)^. However, the lack of inclusion of school-age children (5–9 years) and adolescents in research, policy and programming limits our understanding of the burden of malnutrition in these age groups, as well as of effective interventions^(6–8)^.

As children transition through adolescence and puberty, they experience rapid physical, behavioural and emotional growth. They also gain independence and the social determinants of their health behaviours broaden^(9)^. During this time, adolescents may be highly susceptible to the adoption of unhealthy lifestyles (including diet and activity behaviours), which can persist into adulthood^(9,10)^. In low- and middle-income countries (LMIC), this is exacerbated by rapid urbanisation, which exposes adolescents to increasingly obesogenic environments characterised by energy-dense and ultra-processed diets and low levels of physical activity, often alongside food insecurity^(11–13)^.

According to the latest global Demographic and Health Survey data for non-pregnant adolescent girls (15–19 years) (Lelijveld *et al*., unpublished results), the prevalence of thinness ranges between 2 % in the Europe and Central Asia (ECA) region and 7 % in South Asia (SA). On the other hand, the East Asia and Pacific (EAP) region demonstrates a 4 % prevalence of overweight and obesity, compared with a prevalence of 40 % in Middle East and North Africa (MENA) region. Anaemia prevalence has remained high, affecting approximately 26 % of adolescent girls in ECA and 52 % in West and Central Africa (WCA). Given the rapidly transitioning patterns of malnutrition experienced in LMIC, as well as the limited inclusion of both girls and boys during later childhood and adolescence in monitoring of nutrition indicators at national and regional levels, exploration into available data describing the burden of malnutrition within and between regions is needed. In addition, better understanding of the determinants of malnutrition in these age groups, including dietary patterns, as well as differences according to age, sex and setting (e.g. rural *v*. urban), would be valuable in developing and targeting interventions.

To date, few interventions have been implemented to improve nutritional status in 5–19-year-olds. Additionally, while previous systematic reviews have explored evidence from interventions which tackle specific nutrition indicators (e.g. overweight and obesity^(14)^) and/or via specific approaches (e.g. lifestyle modification^(15)^) reviews have failed to explore interventions targeting all forms of malnutrition in LMIC via diverse approaches, while taking into account context-specific prevalence and determinants of malnutrition across global regions.

Thus, this review aims to (1) summarise available evidence on the nutritional status of school-age children and adolescents (5–19 years) from LMIC in seven global regions and (2) describe evidence on the design, primary outcome(s) and main findings of interventions implemented to improve malnutrition in this population.

## Methods

In preparing this paper, data were compiled from seven scoping literature reviews conducted by the authors focusing on LMIC within the following seven UNICEF-defined world regions: EAP; ECA; SA; WCA; Eastern and Southern Africa (ESA); MENA; and Latin America and the Caribbean (LAC). Due to the heterogeneity in the availability, design and outcomes of studies across countries, scoping review methodology was used to ensure that studies were not excluded based on systematic review criteria and that research gaps related to the heterogeneity of research in the area could be identified. All reviews were conducted between January 2020 and January 2021 to summarise the nutritional status of school-age children and adolescents (5–19 years old), as well as interventions implemented to target malnutrition in this population.

### Search strategy

Literature searches were carried out using the PubMed, Embase, Cochrane, Web of Science (ESA and MENA), Africa Wide Information (ESA), ADOLEC (LAC), Global Health (EAP) and ScienceDirect (ECA) databases. A population, interventions, control and outcome (PICO) framework (Table 1) was used to define search terms (Supplementary Appendix A) and to guide the screening of articles. The results were reported according to the PRISMA guidelines for scoping reviews.

**Table 1:** Population, interventions, control and outcome (PICO) framework

Population	Intervention	Comparison	Outcome
School-age children and adolescents 5–19 years living in low- and middle-income countries (LMIC) in each of the following UNICEF-defined global regions: East Asia and Pacific (EAP); Europe and Central Asia (ECA); South Asia (SA); West and Central Africa (WCA); Eastern and Southern Africa (ESA); Middle East and North Africa (MENA); Latin America and the Caribbean (LAC)	None required Any intervention type (ESA, MENA, LAC), or any intervention that successfully improved nutritional status (EAP, ECA, SA, WCA)	None required	Prevalence of: • Stunting • Wasting • Thinness • Overweight and/or obesity • Anaemia and/or Fe deficiency • Iodine deficiency and/or goitre • Vitamin A deficiency • Vitamin D deficiency • Zn deficiency • Dietary intakes and/or patterns

Outcome definitions and classifications: stunting, height-for-age <−2 standard deviations (sd) below the WHO Child Growth Reference median; wasting, weight-for-height <−2 sd below the WHO Child Growth Reference median^(90)^; thinness, BMI-for-age <−2 sd below the WHO Growth Reference median^(90)^; overweight, BMI-for-age >+1 sd above the WHO Growth Reference median OR BMI-for-age expressed as ≥85th percentile of the CDC growth reference OR BMI-for-age equivalent to BMI ≥25 kg/m^2^ using International Obesity Task Force (IOTF) cut-offs by sex; obese, BMI-for-age ≥+2 sd above the WHO Growth Reference median OR BMI-for-age expressed as ≥95th percentile of the CDC growth reference OR BMI-for-age equivalent to BMI ≥30 kg/m^2^ using IOTF cut-offs by sex^(90–92)^; anaemia, children <12 years (haemoglobin, Hb, <115 g/l), children 12–14 years and females 15+ years (Hb <120 g/l), males 15+ years (Hb <130 g/l); iron deficiency, serum ferritin <15 µg/l; iron-deficiency anaemia, various classifications used but involve a combination of anaemia and concurrent low ferritin level (iron deficiency + Hb <120 g/l)^(93)^; iodine deficiency, median urinary iodine concentration (UIC) <100 µg/l^(94)^; goitre, grade 1 – a goitre that is palpable but not visible when the neck is in the normal position (i.e. the thyroid gland is not visibly enlarged), nodules in a thyroid that is otherwise not enlarged fall into this category; grade 2 – a swelling in the neck that is clearly visible when the neck is in a normal position and is consistent with an enlarged thyroid gland when the neck is palpated^(95)^; vitamin A deficiency, serum retinol concentrations ≤0·70 μmol/l^(96)^; vitamin D deficiency, 25(OH)D <20 ng/ml (deficiency), 25(OH)D 21–29 ng/ml (insufficiency)^(97)^; zinc deficiency, children <10 years (serum zinc <65 ug/dl)^(98)^.

### Screening of articles and selection of studies

For all of the reviews, the search results were extracted and imported into reference management software (Mendeley or EndNote X9). All duplicates were removed, and the results were screened by a single researcher, based on their title and abstract. Full texts were then screened for eligibility according to the following criteria:

### Inclusion criteria

- Guided by the PICO framework (Table 1)
- Peer-reviewed publications, with conference abstracts (LAC; EAP; ECA) and grey literature (EAP) included in some cases
- Only human studies
- No restrictions on study design
- Articles in English, Portuguese, French or Spanish
- Articles published since 2005 (ECA, ESA, MENA), 2010 (LAC, EAP, WCA) and 2016 (SA, due to the availability of a UNICEF analysis, including literature published between 1990 and 2015^(16)^

### Exclusion criteria

- Children and/or adolescents with a diagnosed disease (including COVID-19), with an addiction, or who were pregnant
- EAP and SA reviews excluded studies with a sample size of *n* < 100, due to the large number of results

### Data extraction

Data for the following outcomes of interest were extracted from relevant research articles for each regional literature review: stunting, wasting; thinness, overweight and/or obesity, anaemia and/or Fe deficiency, iodine deficiency and/or goitre, vitamin A deficiency, vitamin D deficiency, Zn deficiency and dietary intakes and/or patterns. Definitions and classifications of outcomes are provided with Table 1. Results are pooled by region to allow for regional comparisons. Available data describing determinants of nutrition outcomes, including differences by age, sex and setting (rural *v*. urban), are also discussed in narrative findings per outcome.

Since this paper aimed to provide a comprehensive overview of the available literature describing the nutritional status of adolescents and school-age children, and because the number of studies for individual countries and/or outcomes of interest varied substantially within and across regions, a formal risk of bias assessment was not carried out, in order to avoid excluding relevant studies. Due to the heterogeneity of studies and the limited results for many countries, it was not possible to conduct a meta-analysis or to apply weighting criteria to individual study results. The regional prevalence ranges provided are therefore based on the lowest and highest prevalence described across countries within the region.

## Results

### Availability of research articles

**Fig. 1:**
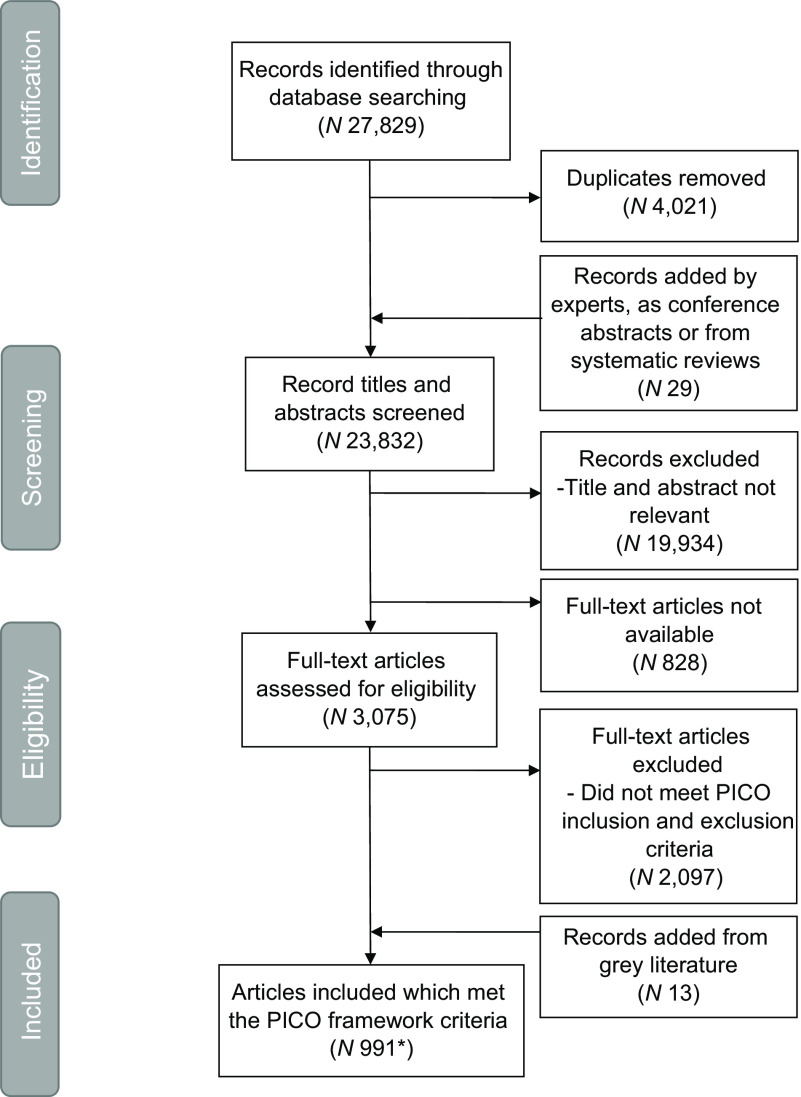
Flow diagram of search results. PICO, Population, Interventions, Control and Outcome; *Of the 991 articles included, the breakdown of articles per region was: South Asia, *n* 247; West and Central Africa, *n* 193; Europe and Central Asia, *n* 122; Eastern and Southern Africa, *n* 130; Middle East and North Africa, *n* 120; East Asia and Pacific, *n* 112; Latin America and the Caribbean, *n* 67

Figure 1 presents a flow diagram of the search results across the seven literature reviews. A total of 27 829 articles were identified for title and abstract screening. Following full text screening of 3075 articles, a total of 991 were included (a full list of all included studies by region can be found in Supplementary Appendix B).

**Fig. 2:**
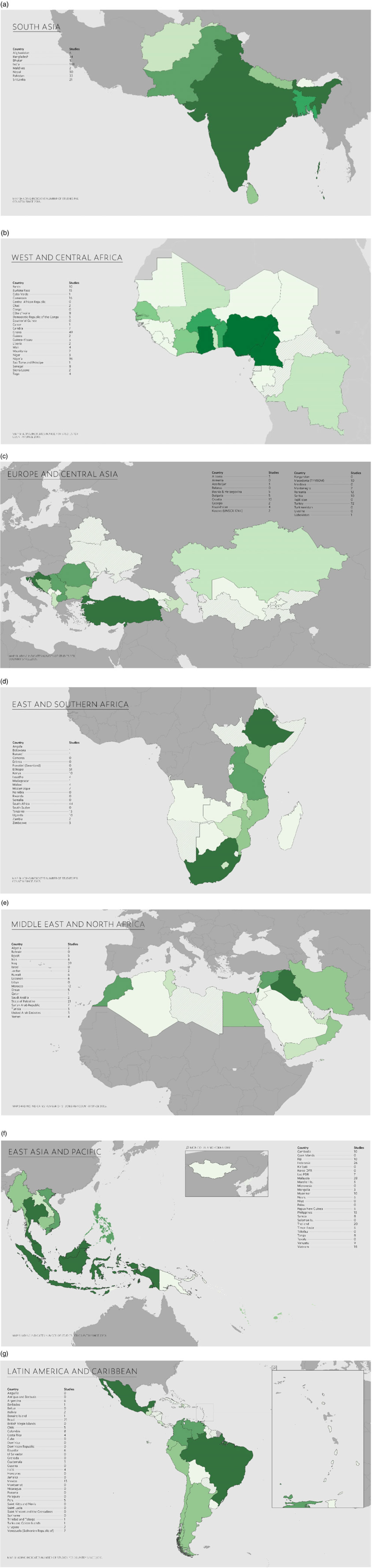
Maps depicting the number of studies with data available from single-country and multi-country studies in the following regions: a, South Asia; b, West and Central Africa; c, Europe and Central Asia; d, Eastern and Southern Africa; e, Middle East and North Africa; f, East Asia and the Pacific; g, Latin America and Caribbean. (a) 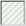, 0; 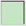, 4–17; 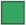, 31–39; 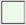, 1–3; 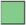, 8–30; 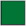, 40+; (b), 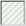, 0; 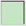, 3–5; 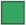, 9–15; 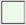, 1–2; 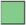, 6–8; 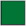, 16+; (c) 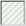, 0; 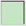, 3–4; 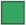, 8–12; 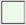, 1–2; 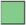, 5–7; 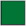, 3+; (d) 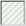, 0; 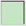, 4–17; 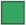, 31–33; 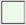, 1–3; 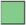, 8–30; 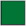, 34+; (e) 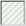, 0; 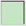, 3–4; 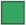, 7–13; 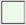, 1–2; 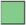, 5–6; 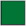, 4+; (f) 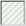, 0; 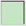, 3–4; 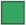, 7–13; 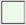, 1–2; 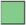, 5–6; 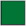, 4+; (g) 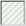, 0; 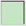, 3–4; 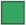, 6–8; 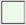, 1–2; 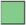, 5–6; 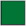, 9+

Summaries of the available evidence for countries within each region are depicted in Fig. 2(a) to (g). SA was the region represented in the largest number of studies (*n* 247), with most data available from India (*n* 144). Nigeria, in WCA, was also represented in a large number of studies (*n* 96), as was Turkey (*n* 72) in ECA region. There were large discrepancies in the availability of evidence across contexts, and even within regions. For example, WCA had a relatively large number of total studies (*n* 193); however, twelve of the twenty-four countries were represented in only three or fewer studies. A particularly low number of published studies were available for the LAC region (*n* 96), with twenty out of thirty-seven countries having no relevant published studies.

**Fig. 3:**
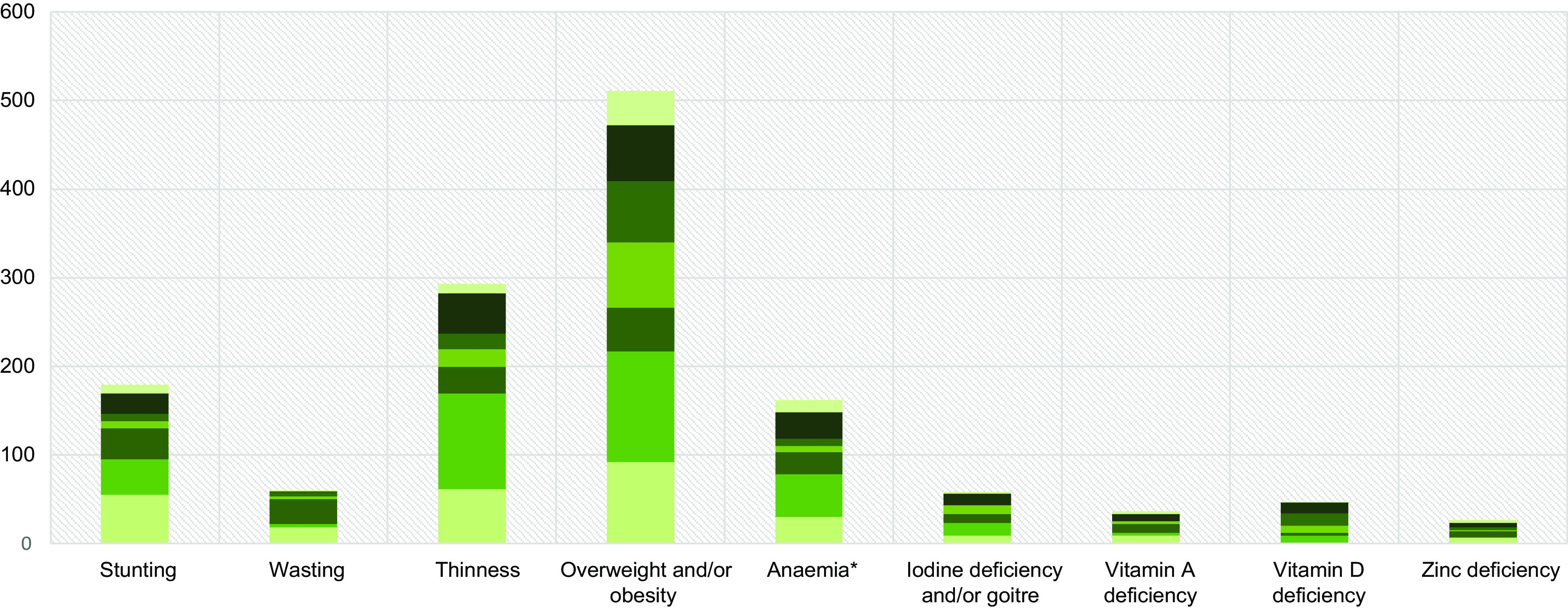
Total number of studies reporting data for anthropometric indicators, anaemia and micronutrient deficiencies by region. *Includes studies that also provide data for iron (Fe) deficiency and/or Fe deficiency anaemia. 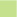, West and Central Africa; 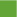, South Asia; 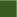, Eastern and Southern Africa; 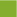, Europe and Central Asia; 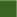, Middle East and North Africa; 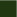, East Asia and the Pacific; 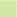, Latin America and the Caribbean

Figure 3 depicts the number of studies reporting data for each nutrition outcome by global region. A summary of the number of studies reporting data for anthropometric indicators and anaemia by country and region is provided in Supplementary Appendix C. The majority of studies reported data for the prevalence of overweight and/or obesity (*n* 511), followed by thinness (*n* 293), with the highest number of studies for both indicators coming from SA (*n* 125 and *n* 108, respectively) and the lowest from LAC region (*n* 39 and *n* 11, respectively). Data describing the prevalence of stunting (*n* 179) and wasting (*n* 60) were more limited. The region with the most studies on stunting prevalence was WCA (*n* 55), while ECA and MENA had the fewest studies (*n* 8 each). Almost half (*n* 28) of the studies presenting data on wasting prevalence were conducted in ESA (mainly in Ethiopia, *n* 14), with eighteen studies being available for WCA (mainly in Nigeria, *n* 10). For the remaining regions, between one and five studies presented data on wasting prevalence. The majority of studies reporting data on the prevalence of anaemia and/or Fe deficiency and/or Fe deficiency anaemia (IDA) (*n* 162) came from SA (*n* 48) and the least from MENA (*n* 8) and ECA (*n* 7). Fewer studies were available for other micronutrient deficiencies: iodine deficiency and/or goitre (*n* 58), vitamin A deficiency (*n* 36), vitamin D deficiency (*n* 47) and Zn deficiency (*n* 26). The availability of evidence varied by region, with SA (*n* 14) and EAP (*n* 13) predominantly reporting data on iodine deficiency and/or goitre, ESA (*n* 10) and WCA (*n* 9) predominantly reporting data on vitamin A deficiency and MENA (*n* 14) predominantly reporting data on vitamin D deficiency.

### Narrative results per nutrition outcome

#### Stunting

**Fig. 4:**
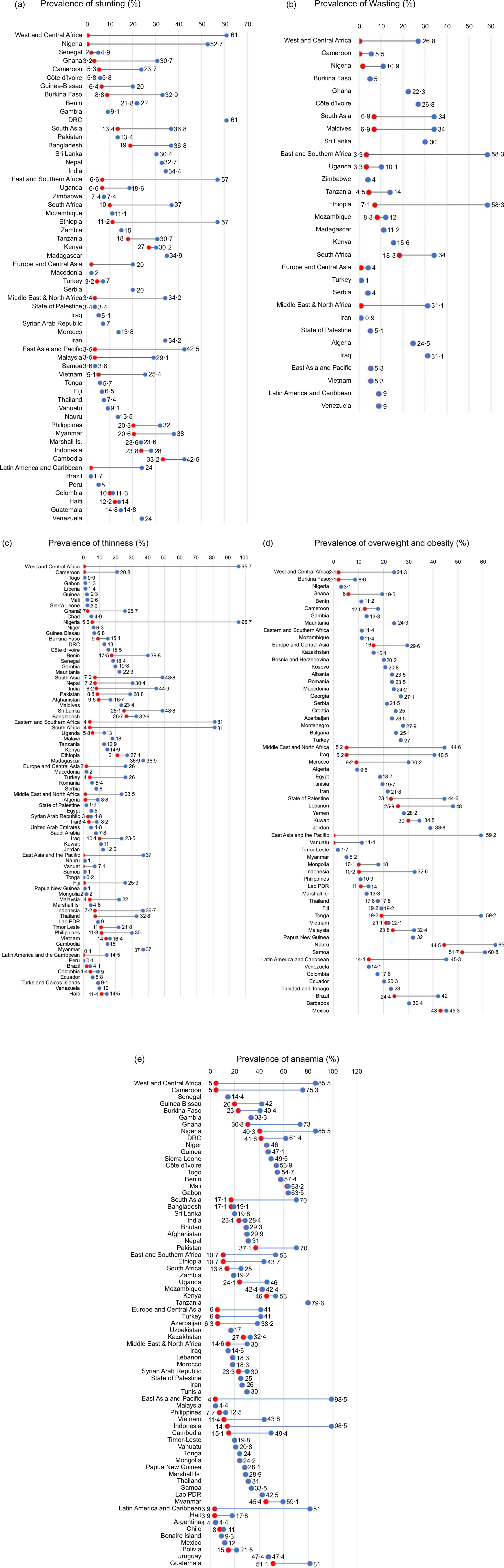
Range in prevalence of nutrition indicators by country and region for: a, stunting; b, wasting; c, thinness; d: overweight and obesity; e, anaemia; red circles depict the lowest and blue circles the highest prevalence in the range; where only one value was available for a country only a single (blue) circle is depicted

The lowest prevalence of stunting was documented in Brazil (1·7 %)^(17)^, in LAC region, and the highest was in the Democratic Republic of the Congo (61·0 %)^(18)^, in WCA (Fig. 4(a) and Supplementary Appendix D). Results for sex differences in stunting prevalence varied between studies; however, the prevalence was consistently higher in boys in ESA and WCA. One study in LAC demonstrated a higher risk of severe stunting in boys^(19)^. While longitudinal data from EAP showed a decline in stunting prevalence with age (up to 15 years)^(20)^, cross-sectional data from EAP and SA suggested a higher prevalence of stunting at older ages^(21–28)^. Across regions (LAC, ESA, WCA, SA), rural children and adolescents were more likely to be stunted than their urban counterparts. Other predictors of stunting included malaria parasitaemia, low socio-economic status (SES), parental (or father’s) unemployment status, a lower level of maternal education, attending a public *v*. a private school and being born to a single mother with higher parity. Lower nutrition knowledge, skipping breakfast, infrequently consuming snacks and having lower poultry consumption were also associated with higher stunting prevalence. Data from LAC and WCA showed that stunted children were more likely to be anaemic^(29)^ and to have low memory and cognitive scores^(30)^, respectively.

#### Wasting

The lowest documented prevalence of wasting was in WCA region (0·3 % in Cameroon)^(31)^, and the highest was in Ethiopia (58·3 %), in ESA^(32)^ (Fig. 4(b) and Supplementary Appendix D). Data from ESA showed differences in how wasting prevalence have changed over time, with repeated cross-sectional surveys conducted in Mozambique between 1992 and 2012 showing a decline from 19·0 % to 12·0 % (8–15 years of age)^(33)^, while national data from Zimbabwe indicated a very slight increase in wasting prevalence from 3·1 % in 2009 to 3·6 % in 2011^(34)^. In WCA region, public (*v*. private) schooling, a rural (*v*. an urban) dwelling and low (*v*. high) SES were predictors of wasting in school-age children and adolescents.

#### Thinness

Nauru and Vanuatu, in EAP region, had the lowest documented prevalence of thinness (0·0 %)^(35,36)^, while the highest prevalence was documented in Nigeria (95·7 % (within country range: 5·6 %–95·7 %))^(37)^, in WCA (Fig. 4(c) and Supplementary Appendix D). There was substantial variation in the prevalence of thinness within regions, with the lowest prevalence in WCA being 0·6 % in Cameroon^(38)^. Pooled analysis in SA found that, while the prevalence of thinness had declined over time, the overall burden had increased, due to growing population sizes^(39)^. Data from WCA, MENA, ESA and EAP showed a higher prevalence of thinness in boys compared with girls. The same pooled analysis of national and sub-national surveys in SA also showed a higher prevalence of thinness in boys^(39)^; however, other individual studies from SA showed mixed evidence for sex differences. Older age was positively associated with thinness in LAC and MENA, but negatively associated in SA. In WCA, public (*v*. private) schooling, a rural (*v*. an urban) dwelling and low (*v*. high) SES were predictors of thinness. Thinness was also associated with public (*v*. private) schooling in SA, and with lower SES in SA and ECA. Rural dwelling was positively associated with thinness in some, but not all, studies in SA. Other predictors included larger family size and living in joint families, lower levels of maternal education and father’s literacy, poor dietary diversity, poor water, sanitation and hygiene (WASH) practices and a history of illness. Food insecurity and maternal unemployment were predictors of thinness in MENA.

#### Overweight and obesity

The range of overweight and obesity prevalence varied widely within and between regions (Fig. 4(d) and Supplementary Appendix D). The lowest documented prevalence rates for overweight and obesity were 0·0 %, using data from Nigeria (WCA)^(40–42)^. The highest prevalence of overweight was 73·0 % in Brazil (LAC)^(43)^, and the highest obesity prevalence was 37·2 % (Nigeria in WCA and Venezuela in LAC)^(44,45)^.

In some regions (LAC, MENA, SA), results for sex differences in overweight and obesity prevalence were mixed; however, studies in WCA and ESA showed consistently higher overweight and/or obesity prevalence rates in girls. Age-related differences in prevalence also varied, with studies in EAP and ECA suggesting higher prevalence of overweight and obesity in younger ages and those in ESA suggesting an increase with age. Private schooling (LAC, ESA, SA), urban dwelling (ECA, ESA, SA, WCA) and higher SES (ECA, ESA, SA, MENA, WCA) were consistently identified as risk factors for overweight and obesity. Dietary behaviours positively associated with overweight and obesity across regions included higher intakes of processed snacks, sugar-sweetened beverages, fast-food, total sugar and lower intakes of fruit and vegetables, as well as skipping breakfast, purchasing food at school, visiting restaurants more frequently and purchasing more food from street vendors. Lower levels of physical activity and/or more time spent being sedentary or using screens on digital devices also increased the risk of overweight and obesity, while higher levels of maternal/parental education reduced the risk. A higher maternal BMI and parental obesity, as well as higher family income and being more food secure, were positively associated with overweight and obesity in adolescents. Data from WCA showed that adolescents with overweight and obesity were more likely to have high systolic blood pressure and a distorted body image.

### Micronutrient deficiencies

#### Anaemia and iron

There were wide ranges in anaemia prevalence documented within countries, as well as between countries and regions (Fig. 4(e) and Supplementary Appendix D). The lowest prevalence of anaemia was found in LAC region (3·9 %, in Mexico)^(46)^ and the highest in EAP (98·5 %, in Indonesia)^(47)^. Fewer studies reported on the prevalence of Fe deficiency and/or IDA; however, available data showed that Fe deficiency prevalence ranged from 3·7 % in Nepal (SA)^(48)^ to 71·3 % in Ghana (WCA)^(49)^, with IDA prevalence ranging from 0·2 % in Malaysia (EAP)^(50)^ to 37·4 % in Ethiopia (ESA)^(51)^. In LAC, the prevalence of anaemia (and of stunting and thinness) was consistently high in studies from Haiti and Venezuela, as well as in indigenous and underserved/rural (*v*. urban) populations, children and adolescents attending public (*v*. private) schools and for children and adolescents from low (*v*. high) SES households. Similar findings for associations between living in slum or rural areas and low SES and anaemia prevalence were documented in ECA, ESA and SA. Other predictors of anaemia in SA included low dietary diversity – particularly linked to irregular consumption of green leafy vegetables, milk, egg, fruits and meat – worm infestation, lack of Fe and folic acid supplementation and poor hand washing practices. There were mixed results for age- and sex-related differences in anaemia prevalence, with studies from some regions (LAC and WCA) presenting inconsistent results. For studies from other regions, some showed higher anaemia prevalence in girls (SA) and at older ages (SA, MENA), while others indicated higher prevalence in boys (ECA) or at younger ages (EAP).

#### Iodine and goitre

Across regions, data suggested low prevalence of iodine deficiency and/or goitre overall, due to implementation of effective salt iodisation programmes (Table 2). For example, nationally representative longitudinal data from Sri Lanka showed a reduction in the prevalence of iodine deficiency (2·7 % to 1·6 %) and goitre (18·0 % to 1·9 %) between 2000 and 2016^(52)^. However, there was variation within and between regions, with both the lowest (0·0 % in Indonesia)^(53)^ and the highest (88·1 % in Papua New Guinea)^(54)^ prevalence of iodine deficiency documented in EAP region. Data from studies in ECA, SA and WCA suggested a greater likelihood of iodine deficiency in girls and at older ages.

**Table 2:** Summary of available studies describing prevalence of micronutrient deficiencies in school-age children and adolescents, by region

Region	Iodine deficiency and/or goiter				Vitamin A deficiency			Vitamin D deficiency			Zn deficiency		
	*n*	*n* girls only	Iodine deficiency prevalence (% range)	Goitre prevalence (% range)	*n*	*n* girls only	Prevalence (% range)	*n*	*n* girls only	Prevalence (% range)	*n*	*n* girls only	Prevalence (% range)
West and Central Africa	9		1·6; 56·2	2·2; 73·5	9		3·0; 51·1	0			6		13·6; 69·0
South Asia	13	1	6·3; 31·8	2·2; 9·3	3		1·2; 74·0	9		8·4; 93·0	1		54·0
Eastern and Southern Africa	10		3·4; 86·0		8	2	4·1; 70·9	3		7·0; 42·0	6	1	3·0; 75·6
Europe and Central Asia	10		2·0; 13·0	7·0; 57·6	2	1*	0·0; 21·0	7	1	21·0; 80·0	1		27·8
Middle East and North Africa	0				0			12	2; 1, boys only	18·8; 100·0	3		12·4; 12·7
East Asia and the Pacific	13		0·0; 88·1	2·1; 9·3	8		1·0; 11·0	12		15·0; 100·0	5		21·6; 69·6
Latin America and Caribbean	2		5·3; 10·0		3		0·5; 11·6	1		5·0; 21·0	3		12·9; 21·6

* Study conducted in women of reproductive age (15–49 years).

Iodine deficiency, median urinary iodine concentration (UIC) <100 µg/l^(94)^; goitre, grade 1 – a goitre that is palpable but not visible when the neck is in the normal position (i.e. the thyroid gland is not visibly enlarged), nodules in a thyroid that is otherwise not enlarged fall into this category; grade 2 – a swelling in the neck that is clearly visible when the neck is in a normal position and is consistent with an enlarged thyroid gland when the neck is palpated^(95)^; vitamin A deficiency, serum retinol concentrations ≤0·70 μmol/l^(96)^; vitamin D deficiency, 25(OH)D < 20 ng/ml (deficiency), 25(OH)D 21–29 ng/ml (insufficiency)^(97)^; Zn deficiency, children <10 years (serum Zn <65 μg/dl)^(98)^; N indicates number of available articles presenting data on micronutrient deficiencies from single-country studies and from single-country studies including girls only; prevalence range per region based on the lowest and highest prevalence described across countries within the region.

#### Vitamin A

Overall, the prevalence of vitamin A deficiency ranged from 0·0 % in Nigeria (WCA)^(55)^ and Turkey (ECA)^(56)^ to 74·0 % in (SA)^(57)^ (Table 2). There were also wide ranges in the prevalence of vitamin A deficiency documented within countries where more than one study was available (e.g. between 0·0 % and 51·5 % prevalence in Nigeria)^(41,55)^. Few studies disaggregated data by sex and age; however, one study in EAP (Philippines) showed a higher prevalence of vitamin A deficiency in children 6–12 years of age (10·7 %) compared with those 13–19 years of age (4·0 %)^(58)^.

#### Vitamin D

The prevalence of vitamin D deficiency ranged from 5 % in Guatemala (LAC)^(59)^ to 100 % in Mongolia (EAP)^(60)^ and Saudi Arabia (MENA)^(61)^ (Table 2). In EAP and ESA, the prevalence of vitamin D deficiency was higher for urban adolescents than for their rural counterparts. Sex differences were also evident, with lower prevalence rates shown in boys from ECA, MENA and SA – potentially due to higher levels of sun exposure. There was also evidence of seasonal differences in vitamin D deficiency, with comparatively higher risk of deficiency at the end of winter in ECA and in summer in MENA (less time spent outdoors due to high temperatures). Other predictors of vitamin D deficiency in SA included low sun exposure, low Ca intake, use of sun protection and darker skin.

#### Zinc

Both the highest and lowest prevalence of Zn deficiency was documented in ESA; 3 % in Kenya^(62)^ and 75·6 % in South Africa^(63)^ (Table 2). Overall, studies from ESA showed a positive association between prevalence of Zn deficiency and being male, food insecurity and poverty, while data from Turkey (ECA) showed no differences in prevalence according to sex or SES.

#### Dietary intakes and patterns

A total of 241 articles presented data on dietary intakes and patterns. The number of available studies ranged from fifty-four in WCA to eighteen in ESA. Methods for describing diets of school-age children and adolescents varied between studies; however, some commonly used indicators included assessment of food insecurity, nutrition knowledge, dietary intakes and patterns, dietary diversity, breakfast consumption and junk/fast-food consumption. Data collection for these indicators was questionnaire-based and included dietary assessment via FFQ (quantitative, semi-quantitative or non-quantitative) or 24-h-recall questionnaires (either on single or multiple days), as well as various other questionnaire tools. A summary of the commonly assessed dietary indicators and the main findings per region are provided in Table 3.

**Table 3:**
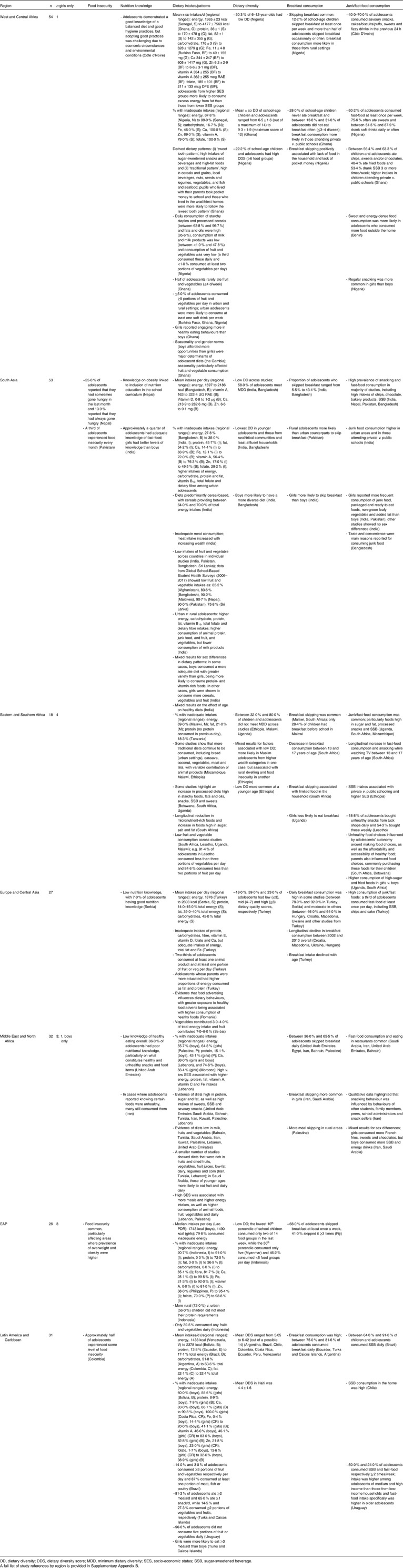
Summary of commonly reported dietary indicators and main findings per region

Overall, the findings showed that school-age children and adolescents commonly skipped breakfast, with some studies suggesting that this was more likely in girls and in those from rural areas, as well as those experiencing greater household poverty. Food insecurity and a lack of affordability of, and access to, food (healthy diets in particular) was a strong driver of poor dietary diversity and unhealthy food choices. In contrast, some studies showed a reduction in dietary diversity for higher wealth categories that was often coupled with higher junk/fast-food consumption. There was evidence of transitioning dietary patterns across regions, with diets varying between more traditional patterns (e.g. high intakes of cereals and grains, vegetables, nuts, seeds and legumes, coconut, local beverages and meat) and patterns increasingly high in processed foods, fats and oils, snacks, SSB and sweets. This was echoed in the food practices reported in all regions, where snacking, eating in restaurants and consuming high quantities of junk/fast-foods were increasingly common, and where intakes of micronutrient-rich fruits and vegetables were low. Where data on nutrition knowledge were available, this was found to be poor in MENA and ECA (between 7 % and 14 % demonstrating moderate or good levels of knowledge), but higher in SA and WCA. In Nepal (SA) specifically, knowledge around obesity was attributed to the inclusion of nutrition education in the school curriculum. However, even in cases where adolescents reported having good knowledge on what constituted unhealthy foods, these items were commonly consumed. Besides affordability and access, dietary behaviours were influenced by a range of factors, including food advertising, children and adolescents’ level of autonomy around food choices, seasonality and the views or practices of family members, peers and school administrators.

#### Interventions

A summary of the intervention studies, identified according to intervention type and primary outcome, is provided in Table 4. Intervention studies targeting malnutrition in school-age children and adolescents were limited in all regions (*n* 103), with the most studies available in LAC (*n* 30). Little evidence was available from regions in Africa, especially in ESA (*n* 7) and from African countries in MENA (*n* 1). In regions where intervention studies were available, few countries were represented. For example, in LAC, two-thirds of the interventions took place in Mexico and Brazil (*n* 10 each).

**Table 4:**
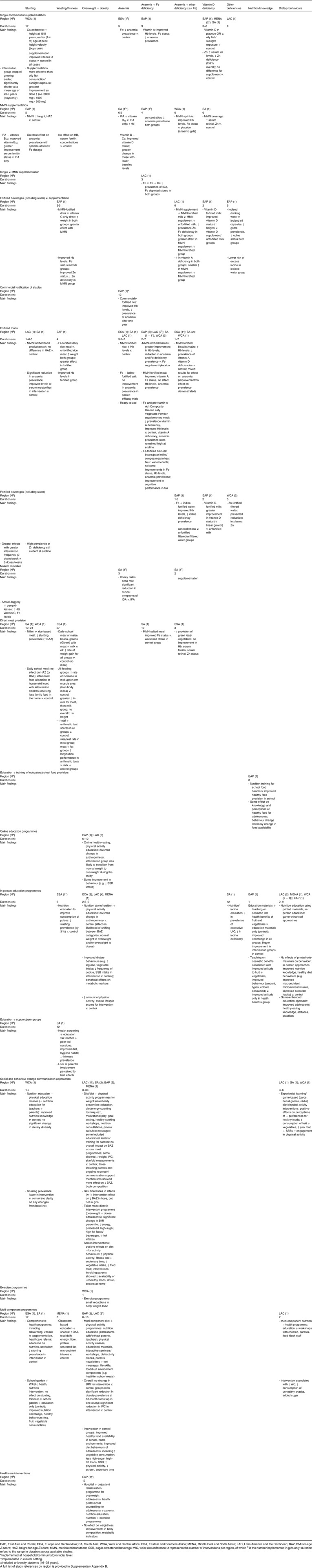
Summary of intervention studies across world regions, according to intervention type and primary outcome

The primary outcomes of interventions were anaemia and/or micronutrient deficiencies (*n* 42, 41 % of intervention studies); overweight and obesity (*n* 33, 32 %); undernutrition (stunting, wasting or thinness) (*n* 18, 17 %) and improved nutrition knowledge or dietary practices (*n* 10, 10 %). Most interventions included boys and girls, with a total of 10 (10 %) including girls only. A total of twenty-two intervention studies targeted school-age children (5–9 years), forty-three targeted adolescents (10–19 years) and the remaining thirty-eight included participants across these age groups. The majority of interventions were implemented in schools (*n* 98, 95 %), with three being implemented at community or provincial level, one in a clinical setting and one in a university setting.

Interventions targeting micronutrient deficiencies (*n* 42) focused primarily on reducing the prevalence of anaemia and/or Fe deficiency via supplementation and fortified food (e.g. biscuits or meals) and beverages (e.g. milk or water) (*n* 20). The remaining micronutrient interventions either incorporated other micronutrients (iodine, vitamin A or multiple micronutrients; MMN) within anaemia reduction programmes (*n* 8) or targeted other single-micronutrient deficiencies, including vitamin D (*n* 7), iodine (*n* 1), Zn (*n* 3) and Ca (*n* 2). The duration of interventions ranged from one to 12 months. Overall, benefits of supplementation and fortification were seen in improved Fe and vitamin D status, as well as Hb levels, reduced anaemia prevalence and reduced prevalence of vitamin A, Zn and iodine deficiencies in some cases. There was evidence of greater improvement in these outcomes from using fortification as compared with using supplementation and from using MMN approaches as compared with single micronutrients (particularly Fe) or iron and folic acid. Supplementation with vitamin D facilitated greater improvement in vitamin D status than either sunlight exposure or oily fish consumption, and while both iodised oil capsules and iodised water increased urinary iodine concentration (UIC), there was a greater risk of excess in those receiving capsules. One study in the Gambia showed that, while Ca supplementation facilitated greater linear growth and higher stature at 15 years of age for boys, it also resulted in earlier cessation of growth and shorter stature in adulthood (23 years)^(64)^.

The majority of interventions addressing undernutrition (*n* 18) took place in SA (*n* 5) and WCA (*n* 4); the duration of interventions ranged from one to 27 months. The majority of interventions addressing overweight and obesity (*n* 33) took place in LAC (*n* 19) and EAP (*n* 6) and ranged from 2·5 to 36 months in duration. The provision of food or beverages, particularly fortified with MMN, was associated with reductions in stunting and/or thinness. In cases where fortified foods did not effectively reduce stunting prevalence, beneficial effects on micronutrient status and anaemia prevalence were shown. In one study from Kenya, while food provided at school had no overall effect on height, gains in weight, lean muscle mass and cognitive function were observed, particularly when the meal included meat, rather than milk or oil^(65)^. Providing nutrition education and ‘healthy’ snacks (including sandwiches and fruit) in schools reduced thinness and increased daily intakes of energy, fibre, protein and micronutrients; however, saturated fat intake also increased. Other multi-component programmes, including a combination of school gardens, nutrition, WASH and health education, supplementation, deworming and healthcare referral, showed mixed effects on thinness and/or stunting; however, those that were ineffective in reducing stunting and thinness prevalence did show positive effects on nutrition knowledge and health behaviours (e.g. increased consumption of fruit and vegetables).

Interventions targeting overweight and obesity comprised a range of social and behaviour change communication (SBCC) approaches. These included educational leaflets, in-person education and counselling, group learning and workshops, as well as interactive approaches (e.g. cooking classes, exercise programmes and game-based methods). Multi-component programmes that included a combination of nutrition education, diet and physical activity programmes, as well as targeting the food environment, the built environment and the building of life skills seemed most effective in improving access to healthy food in school or home environments, as well as improving behaviours related to diets and physical activity. Few interventions had any effect, or a large effect, on the overall prevalence of overweight and obesity; however, some studies documented beneficial effects on weight, body composition (waist circumference and skinfolds) and metabolic markers (e.g. glycaemic control). Programmes that were more successful in facilitating behaviour change and/or anthropometric changes often involved other stakeholders (e.g. parents and teachers) in the intervention programme. There was limited evidence of using novel approaches (e.g. social media platforms and game-based approaches) to engage children and adolescents. However, in Nigeria, two studies that incorporated game-enhanced approaches (board games, clubs and vouchers) to nutrition and health education showed positive effects on participants’ knowledge and perceptions of healthy eating, promoted the consumption of more nutritious foods (e.g. fruits and vegetables) and facilitated more engagement in physical activity^(66,67)^.

## Discussion

This review summarises evidence on the burden of malnutrition in school-age children and adolescents in LMIC from seven global regions, as well as on interventions implemented to improve nutritional status. While a total of 990 studies were identified, large geographical data gaps were found to exist, due to the fact that the bulk of evidence concentrated on a small number of countries. Studies presenting prevalence data focused on certain anthropometric and nutrition indicators, particularly overweight and obesity, thinness and anaemia, with a dearth of evidence available on the prevalence of other micronutrient deficiencies. With the exception of the LAC and EAP regions, there was limited evidence available on interventions targeting the rising burden of overweight and obesity. The majority of interventions targeted micronutrient deficiencies (mainly anaemia and/or Fe deficiency). Thus, there was little acknowledgement across regions of the broader spectrum of nutrition challenges affecting adolescents, the similar and interrelated causes of undernutrition, overweight and obesity and micronutrient deficiencies and the benefits of double-duty actions to address these. In contrast, substantial evidence exists from high-income countries, including detailed data on food intake, the role of food environments and commercial marketing and advertising (traditional and social media) in driving food choices and interventions targeting the rising burden of overweight and obesity^(68,69)^, and programmes such as school feeding are widely available^(70)^, despite the fact that the majority of the world’s children and adolescents (5–19 years) live in LMIC^(71)^.

Despite limitations in data availability, evidence was found of an emerging double burden of malnutrition across world regions, with stunting and thinness remaining especially high in the WCA, ESA and SA regions. Overweight and obesity affected all world regions, especially countries in LAC, EAP and MENA, where prevalence rates were consistently high across countries. All regions were affected by anaemia, although there was variability between countries. Where data were available, most regions experienced high rates of iodine, vitamin A, vitamin D and Zn deficiencies. Dietary transitions associated with rapid urbanisation were evident across all regions, with diets high in energy-dense, processed and micronutrient-poor foods increasingly being consumed.

A large degree of methodological variability was observed across the studies included in this review, limiting our ability to compare findings within and between regions. In particular, inconsistencies in the use of reference populations (WHO, International Obesity Task Force, US Centers for Disease Control and context-specific references in some cases) and thresholds used to classify thinness, overweight and obesity limited the comparability between studies and may account for some of the variability described. Similarly, the lack of a universal method to assess diet diversity and/or quality in school-age children and adolescents meant that a range of methods and indicators were used. Studies lacked consistency in the age ranges examined, with subjects from various subgroups within the 5–19-year age range being included. There was also inadequate availability of sex-disaggregated data, as well as data for other subgroups, such as those out of school, those living in rural (*v*. urban) settings, those with disabilities and those in vulnerable contexts, such as refugee populations. There is a need for consensus on the methodology to be used in assessing the nutritional status of school-age children and adolescents, to allow for comparable assessments of the burden of malnutrition across global regions, as well as to set international and national targets and to monitor progress against agreed targets^(72)^.

A range of risk factors for nutrition outcomes were identified across studies. Our findings suggest that undernutrition (stunting, wasting and thinness) is more prevalent in boys, and overweight and obesity are more prevalent in girls, particularly as children age and transition through puberty and beyond. As supported by previous research, this may in part be because girls are less likely to be physically active and more likely to engage in sedentary behaviours (e.g. watching television)^(73–75)^, as well as being more likely to skip breakfast and to consume foods high in fat and sugar. An absence of sex-related trends in food security and dietary patterns have also been demonstrated^(73)^.

There was consistent evidence that urban dwelling, being from a higher socioeconomic background, being more food secure and consuming breakfast and healthy snacks decreased the risk of undernutrition. Conversely, in more obesogenic environments, these same factors were associated with higher consumption of processed and junk/fast-food items, a greater likelihood of eating outside the home and a higher risk of overweight and obesity. In both cases, micronutrient deficiencies persisted. This highlights the increasingly ubiquitous nature of malnutrition in LMIC, and that, despite variations in nutritional outcomes between contexts, interventions that improve access to, and consumption of, healthy, diverse diets should be prioritised as double-duty actions to improve outcomes across the malnutrition spectrum. However, better insight into what drives poor diet and activity behaviours in children and adolescents, and how best to target boys and girls as they age, across diverse contexts, is critical in order to ensure that the barriers that exist to making healthy dietary choices are addressed. This will require a food systems approach to make nutritious, safe, affordable and sustainable diets available, appealing and aspirational, while contributing to long-term environmental sustainability and planetary health^(76,77)^.

Regarding intervention studies, it was found that the majority targeted anaemia through fortification or supplementation programmes, while those aimed at prevention or management of undernutrition, or overweight and obesity, were more limited. There were few studies in general, and there was little comparability between studies on intervention targets and the baseline nutritional status of subjects, as well as the type, dose and duration of the interventions implemented. This made it challenging to draw conclusions regarding effective intervention approaches for school-age children and adolescents.

However, our synthesis of evidence suggests that MMN-fortified foods or beverages may be effective in reducing micronutrient deficiencies, alongside weight gain, in undernourished populations. Supplementation and non-fortified food-based approaches were less successful, as was sunlight exposure in the case of vitamin D. Previous evidence from systematic reviews assessing the effects of fortified foods and beverages in adults, adolescents and children across high-, middle- and low-income countries have demonstrated varied results on anaemia and Fe status, with inconclusive effects on growth, morbidity and cognitive outcomes^(78–80)^. However, these reviews also highlighted similar issues around the comparability of findings. Other evidence has highlighted the benefits of MMN fortification and supplementation as compared with single-micronutrient approaches^(81)^. The recent *Lancet* Series on Maternal and Child Undernutrition Progress highlighted the strong evidence supporting MMN supplementation during the antenatal period for improved birth outcomes and suggested the potential benefit of extending this into the preconception period for adolescent girls and young women in high-burden contexts^(82–84)^. Current WHO guidelines recommend daily Fe-only or weekly Fe and folic acid supplementation in menstruating, non-pregnant women and adolescents, depending on the anaemia burden^(82,83,85)^. However, evidence is lacking on implementation strategies to examine the acceptability and effectiveness of supplementation dose, delivery and duration, either for these guidelines or for MMN approaches^(8)^. Additionally, evidence is scarce regarding the benefits for nutritional status, development and morbidity in school-age children and adolescents in the short and longer term^(86,87)^, and further exploration is needed of the effectiveness of MMN programmes within a package of nutrition-specific and nutrition-sensitive interventions, for girls and boys and across contexts.

Interventions targeting overweight and obesity via education and SBCC strategies showed beneficial effects on nutrition knowledge; however, evidence on the impacts on behaviour change and growth outcomes was inconclusive. Most interventions assessed anthropometric outcomes over a short-term follow-up period (up to a year maximum), with only three studies (in LAC) following up between 18 months and 3 years. There is a need for multi-component programmes that start early and extend through the childhood and adolescent years, which consider the wider food environment, and which involve other stakeholders (peers, family andteachers), in order to facilitate behaviour change, improve dietary diversity and quality and improve nutritional status. Such strategies should provide effective double-duty approaches to promoting healthy growth and body size, as well as tackling micronutrient deficiencies across contexts, and should be proactive in those regions where undernutrition remains the priority and where overweight is rising. The use of novel technological approaches to engaging adolescents, as well as approaches designed by adolescents themselves, both of which were largely absent in our findings, are also critical in order to ensure that interventions appeal to adolescent needs and interests. Interventions that extend beyond schools (e.g. using health platforms and social/community-based platforms) are also needed to address the needs of the approximately one-in-five children and adolescents globally who do not attend school^(88)^.

### Limitations

This review has provided a comprehensive summary of available literature from seven global regions; however, methodological differences between the literature reviews feeding into this synthesis may have limited comparability. For example, three reviews included articles published since 2005 (ECA, ESA, MENA), three included articles published since 2010 (LAC, EAP, WCA) and one included articles published since 2016 (SA). Additionally, the EAP and SA reviews excluded studies with sample sizes of *n* < 100 and the EAP review also included data from grey literature. Despite differences in search criteria, the large number of search results in SA were comparable to those published in the UNICEF review^(16)^, specifically showing persistent burdens of stunting and thinness, alongside emerging burdens of overweight and obesity, which were higher in more affluent, well-educated and urban households. In reviews that did not screen according to sample size, only between 1·5 % and 9·0 % of studies had *n* < 100 subjects, and in the EAP review grey literature corroborated the results from peer-reviewed publications. Thus, these differences were unlikely to substantially influence the overall findings per region. Similarly, due to the wide research and streamlining of methods, for example the databases searched, across literature reviews, it is feasible that some manuscripts from particular regions were overlooked; e.g. regional databases such as SciELO were not searched and may have provided additional studies for the LAC region. Future research that involves more targeted searches by researchers living in LMIC may be beneficial in filling some context-specific gaps in the data identified in this literature review synthesis.

As described above, differences in methodology between individual studies also limited comparability within and between countries and regions. This restricted our ability to draw conclusions on the burden of malnutrition and on effective interventions. It is also an important finding in and of itself: highlighting key research gaps, as well as a need for global consensus on the indicators used to describe nutritional status and to assess dietary quality in school age children and adolescents. In addition, previous findings have demonstrated a high degree of comparability between standards of weight classification (i.e. US Centers for Disease Control, International Obesity Task Force and WHO growth standards) in adolescents and in their associations with health outcomes^(89)^. This suggests that the potential for misclassification of anthropometric outcomes between studies is likely to be small overall.

## Conclusion

Globally, persistent food insecurity, poor growth and micronutrient deficiencies continue to plague school-age children and adolescents, as the new challenge of obesity emerges. Despite this, the persistent absence of these age groups across research, policy and programming means that the limited information available is neither standardised nor comparable. Consensus is urgently needed on methods for assessing nutritional status and its determinants (e.g. dietary intake and quality), in order to set targets and monitor progress. In addition, an understanding of double-duty interventions is needed, to ensure that healthy diets are available, affordable and appealing for all adolescents, alongside efforts to promote healthy behaviours that support optimal growth and development for current and future generations.

## Supplementary material

For supplementary material accompanying this paper visit https://doi.org/10.1017/S1368980022000350. click here to view supplementary material
