# Supplementary material for: Nutritional status of school-age children and adolescents in low- and middle-income countries across seven global regions: a synthesis of scoping reviews
Source: Public Health Nutr. 2022 Feb 14;26(1):63–95. doi: 10.1017/S1368980022000350 (PMC11077463; doi:10.1017/S1368980022000350)
Supplement: Supplementary file 1 [file S1368980022000350sup.zip › S1368980022000350sup001.docx]

**Supplementary Appendix A: Summary of search terms used for region-specific literature reviews as informed by the Population, Interventions, Control and Outcome (PICO) framework**

| ***Population*** | ***Intervention*** | ***Comparison*** | ***Outcome*** |
| --- | --- | --- | --- |
| ((adolescen* or "school aged" or "school children" or "school child" or "school-age" or teen*) OR (exp adolescent/) OR (exp school child/))  **AND**  (Countries OR region names) | Any intervention type (ESA, MENA, LAC), or any intervention that successfully improved nutritional status (EAP, ECA, SA, WCA) | Not required | ((anaemia OR anemia OR iron OR ferrous OR ferric OR hepcidin OR ferritin OR transferrin OR heme OR haem) OR (exp anemia/) OR ( exp iron deficiency anemia/) OR (exp megaloblastic anemia/ ) OR (exp microcytic anemia/) OR (exp macrocytic anemia/) OR (exp normochromic normocytic anemia/) OR (exp iron intake/) OR (exp iron depletion/) OR (exp iron absorption/) OR (exp iron deficiency/) OR (exp iron binding capacity/) OR (exp iron blood level/) OR (exp transferrin receptor/) OR (exp transferrin blood level/) OR (exp transferrin/ ) OR (exp hepcidin/) OR (exp ferritin/))  **OR**  (("vitamin A" OR retinol OR carotenoid* OR xerophthalmia) OR (exp retinol/) OR (exp carotenoid/) OR (exp retinol deficiency/))  **OR**  (("vitamin D" OR "vitamins D deficiency") OR (exp vitamin D/) OR (exp vitamin D deficiency/))  **OR**  ((zinc OR "zinc deficiency") OR (exp zinc/) OR (exp zinc deficiency/))  **OR**  ((calcium OR "calcium deficiency") OR (exp calcium/) OR (exp hypercalcemia/) OR (exp hypocalcaemia/))  **OR**  ((iodine OR "iodine deficiency" OR goitre OR goiter OR "iodised salt" OR "iodized salt") OR (exp iodine deficiency/) OR (exp goiter/) OR (exp iodine/))  **OR**  ((nutritio* OR malnutr* OR micronutrient OR undernutr* OR overnutr* OR undernourish* OR malnourish* OR stunt* OR "stunted growth" OR "growth failure" OR "linear growth" OR "failure to thrive" OR "growth disorder" OR "wasting" OR "wasted" OR overweight OR obes* or "high body mass index" OR "high BMI" OR thinness) OR (exp nutritional status/) OR (exp malnutrition/) OR (exp underweight/) OR exp (exp overnutrition/) OR (exp stunting/) OR (exp growth disorder/) OR (exp failure to thrive/) OR (exp wasting syndrome/) OR (exp obesity/) OR (exp obesity management/) OR (exp diet-induced obesity/))  **OR**  ((diet OR "dietary pattern*" OR "healthy eating" OR "healthy diet" OR "dietary diversity" OR "food recall" OR "diet recall") OR (exp dietary intake/) OR (exp dietary pattern/) |

**Supplementary Appendix B: Summary of references for all included studies according to UNICEF-defined world regions**

**East Asia and Pacific**

1. Abdullah N, Teo P, Huybrechts I, et al. (2013) Infrequent breakfast consumption is associated with higher body adiposity and abdominal obesity in Malaysian school-aged adolescents. *PLoS ONE* **8**, e59297.

2. Agustina R, Nadiya K, Andini EA, et al. (2020) Associations of meal patterning, dietary quality and diversity with anemia and overweight-obesity among Indonesian school-going adolescent girls in west Java. *PLoS ONE* **15**.

3. Ahmad A, Zulaily N, Shahril MR, et al. (2018) Association between socioeconomic status and obesity among 12-year-old Malaysian adolescents. *PLoS ONE* **13**, e0200577.

4. Ahmad Ali Z, Rusidah S, Azli B, et al. (2013) Nutritional status of Malaysian primary school children aged 8-10 years: findings from the 2008 National IDD Survey. *Malaysian Journal of Nutrition* **19**, 149–161.

5. Al-Mekhlafi HM, Al-Zabedi EM, Al-Maktari MT, et al. (2014) Effects of vitamin A supplementation on iron status indices and iron deficiency anaemia: a randomized controlled trial. *Nutrients* **6**, 190–206.

6. Al-Sadat N, Majid HA, Sim PY, et al. (2016) Vitamin D deficiency in Malaysian adolescents aged 13 years: findings from the Malaysian Health and Adolescents Longitudinal Research Team study (MyHeARTs). *BMJ Open* **6**, 010689.

7. Andriastuti M, Ilmana G, Nawangwulan SA, et al. (2019) Prevalence of anemia and iron profile among children and adolescent with low socio-economic status. *International Journal of Pediatrics and Adolescent Medicine*.

8. Angeles-Agdeppa I, Dinney L & Capanzana MV (2019) Usual energy and nutrient intakes and food sources of Filipino children aged 6-12 years from the 2013 National Nutrition Survey. *Nestle Nutrition Institute Workshop Series* **91**, 111–122.

9. Angeles-Agdeppa I, Gayya-Amita PI & Longalong WP (2019) Existence of double burden of malnutrition among Filipino children in the same age-groups and comparison of their usual nutrient intake. *Malaysian Journal of Nutrition* **25**, 445–461.

10. Angeles-Agdeppa I, Magsadia CR & Capanzana MV (2011) Fortified juice drink improved iron and zinc status of schoolchildren. *Asia Pacific Journal of Clinical Nutrition* **20**, 535–543.

11. Angeles-Agdeppa I, Monville-Oro E, Gonsalves JF, et al. (2019) Integrated school based nutrition programme improved the knowledge of mother and schoolchildren. *Maternal & child nutrition* **15 Suppl 3**, e12794.

12. Angeles-Agdeppa I, Saises M, Capanzana M, et al. (2011) Pilot-scale commercialization of iron-fortified rice: effects on anemia status. *Food and nutrition bulletin* **32**, 3–12.

13. Apirajkamol N, Panamonta O & Panamonta M (2016) Increased levels of median urinary iodine excretion of primary school children in the suburban area, Khon Kaen, Thailand. *The Southeast Asian journal of tropical medicine and public health* **47**, 101–8.

14. Aryati A, Nurzaime Z, Nor Saidah AM, et al. (2017) Body weight status of school adolescents in Terengganu, Malaysia: a population baseline study. *BMC Public Health* **17**.

15. Aurino E, Fernandes M & Penny ME (2017) The nutrition transition and adolescents’ diets in low- and middle-income countries: a cross-cohort comparison. *Public health nutrition* **20**, 72–81.

16. Balkish Mahadir N, Siti Zuraidah M, Rashidah A, et al. (2013) Overweight among primary school-age children in Malaysia. *Asia Pacific Journal of Clinical Nutrition* **22**, 408–415.

17. Boonchoo W, Takemi Y, Hayashi F, et al. (2017) Dietary intake and weight status of urban Thai preadolescents in the context of food environment. *Preventive Medicine Reports* **8**, 153–157.

18. Caleyachetty R, Thomas GN, Kengne AP, et al. (2018) The double burden of malnutrition among adolescents: Analysis of data from the Global School-Based Student Health and Health Behavior in School-Aged Children surveys in 57 low- and middle-income countries. *American Journal of Clinical Nutrition* **108**, 414–424.

19. Charles CV, Summerlee AJS & Dewey CE (2012) Anemia in Cambodia: prevalence, etiology and research needs. *Asia Pacific journal of clinical nutrition* **21**, 171–81.

20. Chawla N, Panza A, Sirikulchayanonta C, et al. (2017) Effectiveness of a school-based multicomponent intervention on nutritional status among primary school children in Bangkok, Thailand. *Journal of Ayub Medical College* **29**, 13–20.

21. Chin Y, Woon F, Chong L, et al. (2014) Associations between home environment, behavioral factors and body-mass-index among primary school children in Selangor, Malaysia. *Obesity Reviews* **15**, 107–108.

22. Chong H, Soo T & Rahmah R (2012) Childhood obesity - prevalence among 7 and 8 year old primary school students in Kota Kinabalu. *Medical Journal of Malaysia* **67**, 147–150.

23. Dancause KN, Vilar M, Chan C, et al. (2012) Patterns of childhood and adolescent overweight and obesity during health transition in Vanuatu. *Public health nutrition* **15**, 158–66.

24. Department of Science and Technology - Food and Nutrition Research Institute (DOST - FNRI) (2015) *Anthropometric survey: 2015 updating of the nutritional status of Filipino children and other population groups*. .

25. Dewi YLR, Widardo & Suprapto B (2013) Iron and iodine supplementation in school children in Ngargoyoso sub-district, Karanganyar regency, Central Java, Indonesia. *Journal of Biology, Agriculture and Healthcare* **3**, 88–92.

26. Emi NA, Gan WY, Mohd Shariff Z, et al. (2020) Associations of an empirical dietary pattern with cardiometabolic risk factors in Malaysian adolescents. *Nutrition and Metabolism* **17**, 28.

27. Farapti F, Muji S, Artanti KD, et al. (2019) Highlighting of urinary sodium and potassium among Indonesian schoolchildren aged 9-12 years: the contribution of school food. *Journal of Nutrition and Metabolism* **2019**.

28. Food and Nutrition Research Institute-Department of Science and Technology (FNRI-DOST) (2015) *Philippine 8th National Nutrition Survey: Biochemical Survey*. Republic of the Philippines: Food and Nutrition Research Institute-Department of Science and Technology (FNRI-DOST).

29. Ganmaa D, Stuart JJ, Sumberzul N, et al. (2017) Vitamin D supplementation and growth in urban Mongol school children: results from two randomized clinical trials. *PloS one* **12**, e0175237.

30. Gausman J, Kim R & Subramanian SV (2019) Stunting trajectories from post-infancy to adolescence in Ethiopia, India, Peru, and Vietnam. *Maternal & child nutrition* **15**, e12835.

31. General Directorate of Statistics (GDS), Ministry of Health, Timor-Leste & ICF (2018) *Timor-Leste Demographic and Health Survey 2016*. Dili, Timor-Leste and Rockville, Maryland, USA: GDS and ICF.

32. Goris JM, Temple VJ, Zomerdijk N, et al. (2018) Iodine status of children and knowledge, attitude, practice of iodised salt use in a remote community in Kerema district, Gulf province, Papua New Guinea. *PloS one* **13**, e0197647.

33. Goris JM, Zomerdijk N & Temple VJ (2017) Nutritional status and dietary diversity of Kamea in Gulf Province, Papua New Guineas. *Asia Pacific journal of clinical nutrition* **26**, 665–670.

34. Hidayanty H, Bardosono S, Khusun H, et al. (2016) A social cognitive theory-based programme for eating patterns and sedentary activity among overweight adolescents in Makassar, South Sulawesi : a cluster randomised controlled trial. *Asia Pacific journal of clinical nutrition* **25**, S83–S92.

35. Hoang NTD, Orellana L, Le TD, et al. (2018) Anthropometric status among 6-9-year-old school children in rural areas in Hai Phong City, Vietnam. *Nutrients* **10**.

36. Hong TK, Trang NHHD & Dibley MJ (2013) Changes in adiposity indicators of Ho Chi Minh City adolescents in a 5-year prospective cohort study. *International journal of obesity (2005)* **37**, 1261–7.

37. Horiuchi Y, Kusama K, Kanha S, et al. (2018) Urban-rural differences in nutritional status and dietary intakes of school-aged children in Cambodia. *Nutrients* **11**.

38. Htet MK, Dillon D, Akib A, et al. (2012) Microcytic anaemia predominates in adolescent school girls in the delta region of Myanmar. *Asia Pacific journal of clinical nutrition* **21**, 411–5.

39. Huang C, Tseng C, Chen H, et al. (2016) Iodine nutritional status of school children in Nauru 2015. *Nutrients* **8**, 520.

40. Hussain S, Elnajeh M, Jalaludin MY, et al. (2015) Vitamin D status of healthy adolescents from two states in Malaysia. *International Journal of Pediatric Endocrinology* **2015**.

41. Iodine Global Network I (2019) Global scorecard of iodine nutrition in 2019. https://www.ign.org/newsletter/idd_may19_global_scorecard.pdf.

42. Kamil Abidalhussain A, Saidon A, Yee K, et al. (2011) Assessment of weight status of secondary school children in Selangor, Malaysia. *Australian Journal of Basic and Applied Sciences* **5**, 1675–1682.

43. Kartini A, Suhartono, Pangestuti DR, et al. (2018) Goiter and hypothyroidism among elementary school children in lowland agricultural area, Brebes district Indonesia. *Indian Journal of Public Health Research and Development* **9**, 120–125.

44. Khin Nyo T, Win Lai M, Han W, et al. (2017) Stunting and zinc nutritional status among primary school children in North-Okkalapa Township. *Myanmar Health Sciences Research Journal* **29**, 86–90.

45. Khor GL, Chee WSS, Shariff ZM, et al. (2011) High prevalence of vitamin D insufficiency and its association with BMI-for-age among primary school children in Kuala Lumpur, Malaysia. *BMC public health* **11**, 95.

46. Kim BK, Jeong JY, Seok KH, et al. (2014) Current iodine nutrition status and awareness of iodine deficiency in Tuguegarao, Philippines. *International Journal of Endocrinology* **2014**, 210528.

47. Kremer P, Waqa G, Vanualailai N, et al. (2011) Reducing unhealthy weight gain in Fijian adolescents: results of the Healthy Youth Healthy Communities study. *Obesity reviews : an official journal of the International Association for the Study of Obesity* **12 Suppl 2**, 29–40.

48. Lao Statistics Bureau (2018) *Lao Social Indicator Survey II 2017, Survey Findings Report*. Vientiane, Lao PDR: Lao Statistics Bureau and UNICEF.

49. Le Nguyen BK, Le Thi H, Nguyen Do VA, et al. (2013) Double burden of undernutrition and overnutrition in Vietnam in 2011: results of the SEANUTS study in 0.5-11-year-old children. *The British journal of nutrition* **110 Suppl 3**, S45-56.

50. Le Nguyen Bao K, Tran Thuy N, Nguyen Huu C, et al. (2016) Anemia and iron deficiency in Vietnamese children, 6 to 11 years old. *Asia-Pacific journal of public health* **28**, 94S-102S.

51. Mahaletchumy A, Rampal L & Sharif ZM (2019) Prevalence of overweight/obesity and its associated factors among secondary school students in semi urban area in Malaysia. *Medical Journal of Malaysia* **74**, 513–520.

52. Mahaweerawat U & Somdee T (2018) Iodine fortification of dessert in iodine deficiency prevention program for primary school children, Maha Sarakham Province, Thailand. *Southeast Asian Journal of Tropical Medicine and Public Health* **49**, 502–508.

53. Mai TMT, Pham NO, Tran TMH, et al. (2020) The double burden of malnutrition in Vietnamese school-aged children and adolescents: a rapid shift over a decade in Ho Chi Minh City. *European Journal of Clinical Nutrition*.

54. Majid HA, Ramli L, Ying SP, et al. (2016) Dietary intake among adolescents in a middle-income country: an outcome from the Malaysian health and adolescents longitudinal research team study (the myhearts study). *PLoS ONE* **11**, e0155447.

55. Maramag CC, Ribaya-Mercado JD, Rayco-Solon P, et al. (2010) Influence of carotene-rich vegetable meals on the prevalence of anaemia and iron deficiency in Filipino schoolchildren. *European journal of clinical nutrition* **64**, 468–74.

56. Mayurachat K, Warunee F, Jutamas C, et al. (2013) An intervention study of changing eating behaviors and reducing weight in Thai children aged 10-12. *Pacific rim international journal of nursing research* **17**, 317‐328.

57. Ministry of Health and Sports (MoHS) & ICF (2017) *Myanmar Demographic and Health Survey 2015-16*. Nay Pyi Taw, Myanmar and Rockville, Maryland USA: MoHS and ICF.

58. Ministry of Health Malaysia (2018) *Malaysia National Health and Morbidity Survey 2017: Key Findings from the Adolescent Health and Nutrition Surveys*. Kuala Lumpur, Malaysia: Institute for Public Health, National Institutes of Health, Ministry of Health Malaysia.

59. National Institute of Statistics, Directorate General for Health & ICF International (2015) *Cambodia Demographic and Health Survey 2014*. Phnom Penh, Cambodia, and Rockville, Maryland, USA: National Institute of Statistics, Directorate General for Health, and ICF International.

60. Ng M, Fleming T, Robinson M, et al. (2014) Global, regional, and national prevalence of overweight and obesity in children and adults during 1980–2013: a systematic analysis for the Global Burden of Disease Study 2013. *The Lancet* **384**, 766–781.

61. Ngan HTD, Tuyen LD, Phu PV, et al. (2018) Childhood overweight and obesity amongst primary school children in Hai Phong City, Vietnam. *Asia Pacific journal of clinical nutrition* **27**, 399–405.

62. Nguyen PV, Hong TK, Nguyen DT, et al. (2016) Excessive screen viewing time by adolescents and body fatness in a developing country: Vietnam. *Asia Pacific journal of clinical nutrition* **25**, 174–183.

63. Nguyen Trung H, Sandalinas F, Sesmaisons A de, et al. (2012) Multi-micronutrient-fortified biscuits decreased the prevalence of anaemia and improved iron status, whereas weekly iron supplementation only improved iron status in Vietnamese school children. *British Journal of Nutrition* **108**, 1419–1427.

64. Nonboonyawat T, Pusanasuwannasri W, Chanrat N, et al. (2019) Prevalence and associates of obesity and overweight among school-age children in a rural community of Thailand. *Korean Journal of Pediatrics* **62**, 179–186.

65. Nur Hanisah O & Razalee S (2014) Body weight perception and weight control behaviors among school adolescents in Pulau Pinang. *Pakistan Journal of Nutrition* **13**, 760–767.

66. Oddo VM, Maehara M & Rah JH (2019) Overweight in Indonesia: an observational study of trends and risk factors among adults and children. *BMJ Open* **9**, e031198.

67. Partap U, Young EH, Allotey P, et al. (2019) Characterisation and correlates of stunting among Malaysian children and adolescents aged 6-19 years. *Global health, epidemiology and genomics* **4**, e2.

68. Passmore E & Smith T (2019) Dual burden of stunting and obesity among elementary school children on Majuro, Republic of Marshall Islands. *Hawai’i journal of health & social welfare* **78**, 262–266.

69. Pengpid S & Peltzer K (2015) Overweight and obesity and associated factors among school-aged adolescents in six Pacific Island countries in Oceania. *International journal of environmental research and public health* **12**, 14505–18.

70. Perignon M, Fiorentino M, Khov K, et al. (2016) Impact of multi-micronutrient fortified rice on hemoglobin, iron and vitamin A status of Cambodian schoolchildren: a double-blind cluster-randomized controlled trial. *Nutrients* **8**, 29.

71. Perignon M, Fiorentino M, Kuong K, et al. (2013) Stunting and anemia are prevalent in cambodian school children and affect cognitive performance. *Annals of Nutrition and Metabolism* **63**, 954–955.

72. Pham NK, Sepehri A, Le TM, et al. (2020) Correlates of body mass index among primary school children in Ho Chi Minh City, Vietnam. *Public Health* **181**, 65–72.

73. Phuong Van Ngoc N, Hong T, Truong H, et al. (2013) High prevalence of overweight among adolescents in Ho Chi Minh city, Vietnam. *BMC Public Health* **13**.

74. Poh BK, Ng BK, Siti Haslinda MD, et al. (2013) Nutritional status and dietary intakes of children aged 6 months to 12 years: findings of the Nutrition Survey of Malaysian Children (SEANUTS Malaysia). *The British journal of nutrition* **110 Suppl 3**, S21-35.

75. Porniammongkol O, Yamborisut U, Intajak T, et al. (2011) Iron status of hill tribe children and adolescent boys: a cross sectional study at a welfare center in Chiang Mai, Thailand. *Pakistan Journal of Nutrition* **10**, 1680–5194.

76. Rachmalina R, Khusun H, Salim LB, et al. (2019) Comparing intake adequacy and dietary diversity between adolescent schoolgirls with normal nutritional status (NG) and undernutrition (UG) based on BMI-for-age (BAZ) living in urban slums in Central Jakarta. *Special Issue: Food choice and behaviour in Indonesia: from under- to over- nutrition.* **25**, S19–S32.

77. Ratsavong K, Van Elsacker T, Doungvichit D, et al. (2020) Are dietary intake and nutritional status influenced by gender? The pattern of dietary intake in Lao PDR: a developing country. *Nutrition Journal* **19**, 31.

78. Reesukumal K, Manonukul K, Jirapongsananuruk O, et al. (2015) Hypovitaminosis D in healthy children in Central Thailand: prevalence and risk factors. *BMC public health* **15**, 248.

79. Rerksuppaphol L & Rerksuppaphol S (2017) Internet based obesity prevention program for Thai school children-a randomized control trial. *Journal of clinical and diagnostic research* **11**, SC07‐SC11.

80. Rerksuppaphol S & Rerksuppaphol L (2016) Effect of zinc plus multivitamin supplementation on growth in school children. *Pediatrics international : official journal of the Japan Pediatric Society* **58**, 1193–1199.

81. Rich-Edwards JW, Ganmaa D, Kleinman K, et al. (2011) Randomized trial of fortified milk and supplements to raise 25-hydroxyvitamin D concentrations in schoolchildren in Mongolia. *The American journal of clinical nutrition* **94**, 578–84.

82. Rojroongwasinkul N, Kijboonchoo K, Wimonpeerapattana W, et al. (2013) SEANUTS: the nutritional status and dietary intakes of 0.5-12-year-old Thai children. *The British journal of nutrition* **110 Suppl 3**, S36-44.

83. Rosmawati N NH Manan, W, WM Izani, N, NJ Nurain, N, NH Razlina, AR (2018) Evaluating the implementation of a canteen-based food nutrition intervention among schoolchildren: a prospective intervention study. *Journal of health and translational medicine* **21**, 21‐27.

84. Rusidah S, Wan Nazaimoon WM, Ahmad Ali Z, et al. (2010) Iodine deficiency status and iodised salt consumption in Malaysia: findings from a national iodine deficiency disorders survey. *Asia Pacific Journal of Clinical Nutrition* **19**, 578–585.

85. Samoa Bureau of Statistics (SBS) (2015) *Samoa Demographic and Health Survey 2014*. Apia, Samoa: Census-Surveys and Demography Division, SBS.

86. Sandjaja S, Budiman B, Harahap H, et al. (2013) Food consumption and nutritional and biochemical status of 0.5-12-year-old Indonesian children: the SEANUTS study. *The British journal of nutrition* **110 Suppl 3**, S11-20.

87. Santiprabhob J, Leewanun C, Limprayoon K, et al. (2014) Outcomes of group-based treatment program with parental involvement for the management of childhood and adolescent obesity. *Patient education and counseling* **97**, 67–74.

88. Serafico ME, Perlas LA, Magsadia CR, et al. (2017) Efficacy of Malunggay (Moringa oleifera) leaves in improving the iron and vitamins A and B status of Filipino schoolchildren. *Acta Horticulturae*, 293–301.

89. Sharifah Nur U, Binti Tuan Y, Madya D, et al. (2012) Relationship between dietary pattern and body mass index among primary school children. *Asian Journal of Clinical Nutrition* **4**, 142–150.

90. Sharifah Wajihah W & Rasyidah G (2020) Association between the school environment and children’s body mass index in Terengganu: a cross sectional study. *PLoS ONE* **15**.

91. Sirikulchayanonta C, Pavadhgul P, Chongsuwat R, et al. (2011) Participatory action project in reducing childhood obesity in Thai primary schools. *Asia-Pacific journal of public health* **23**, 917–27.

92. Sitompul M, Ridwan H, Jalal F, et al. (2013) The efficacy of multiple micro nutrients sprinkle to improve iron status of adolescent girls in islamic religious school-tangerang, Indonesia. *Annals of Nutrition and Metabolism* **63**, 802.

93. Soe LT, Fahmida U, Seniati ANL, et al. (2020) Nutrients essential for cognitive function are typical problem nutrients in the diets of Myanmar primary school children: findings of a linear programming analysis. *Food and Nutrition Bulletin* **41**, 211–223.

94. Soesanti F, Pulungan A, Tridjaja B, et al. (2013) Vitamin D profile in healthy children aged 7-12 years old in Indonesia. *International Journal of Pediatric Endocrinology* **2013**.

95. Somsri P, Satheannoppakao W, Tipayamongkholgul M, et al. (2016) A cosmetic content-based nutrition education program improves fruit and vegetable consumption among Grade 11 Thai students. *Journal of nutrition education and behavior* **48**, 190–8.e1.

96. Sumarlan ES, Windiastuti E & Gunardi H (2018) Iron status, prevalence and risk factors of iron deficiency anemia among 12- to 15-year-old adolescent girls from different socioeconomic status in Indonesia. *Makara Journal of Health Research* **22**, 46–52.

97. Suprapto B & Dewi YLR (2012) Long-term effect of iodized water and iodized oil supplementation on total goitre rate and nutritional status of school children in Ngargoyoso sub-district, Karanganyar regency, Central Java, Indonesia. *Journal of Biology, Agriculture and Healthcare* **2**, 128–135.

98. Susanto F (2015) Nutrient intake evaluation among school age children in Jakarta, Indonesia. *Mediterranean Journal of Nutrition and Metabolism* **8**, 243–250.

99. Sutrisna A, Knowles J, Basuni A, et al. (2018) Iodine intake estimation from the consumption of instant noodles, drinking water and household salt in Indonesia. *Nutrients* **10**.

100. Syahrul S, Kimura R, Tsuda A, et al. (2016) Prevalence of underweight and overweight among school-aged children and it’s association with children’s sociodemographic and lifestyle in Indonesia. *International Journal of Nursing Sciences* **3**, 169–177.

101. Thompson-McCormick JJ, Thomas JJ, Bainivualiku A, et al. (2010) Breakfast skipping as a risk correlate of overweight and obesity in school-going ethnic Fijian adolescent girls. *Asia Pacific journal of clinical nutrition* **19**, 372–82.

102. Trang NHHD, Hong TK & Dibley MJ (2012) Cohort profile: Ho Chi Minh City Youth cohort-changes in diet, physical activity, sedentary behaviour and relationship with overweight/obesity in adolescents. *BMJ Open* **2**, e000362.

103. Tuyen LD, Hien VTT, Binh PT, et al. (2016) Calcium and vitamin D deficiency in Vietnamese: recommendations for an intervention strategy. *Journal of nutritional science and vitaminology* **62**, 1–5.

104. UNICEF (2019) The State of the World’s Children 2019. https://data.unicef.org/resources/dataset/sowc-2019-statistical-tables/.

105. VNSO (Vanuatu National Statistics Office) & SPC (Secretariat of the Pacific Community (2014) *Vanuatu Demographic and Health Survey, 2013*. .

106. Wate JT, Snowdon W, Millar L, et al. (2013) Adolescent dietary patterns in Fiji and their relationships with standardized body mass index. *The international journal of behavioral nutrition and physical activity* **10**, 45.

107. World Health Organization Global School-based Student Health Surveys. https://extranet.who.int/ncdsmicrodata/index.php/catalog/GSHS.

108. World Obesity Federation (2019) *Atlas of Childhood Obesity*. 212. London: .

109. Yasmin G, Kustiyah L & Dwiriani C (2019) Stunted children has higher risk of overweight: a study on children aged 6-12 years in eight provinces in indonesia. *Pakistan Journal of Nutrition* **18**, 455–463.

110. Yusni Y & Meutia F (2019) Anthropometry analysis of nutritional indicators in Indonesian adolescents. *Journal of Taibah University Medical Sciences* **14**, 460–465.

111. Zimmermann MB, Hussein I, Al-Ghannami S, et al. (2016) Estimation of the prevalence of inadequate and excessive iodine intakes in school-age children from the adjusted distribution of urinary iodine concentrations from population surveys. *Journal of Nutrition* **146**, 1204–1211.

112. Zuraida R, Lipoeto NI, Masrul M, et al. (2020) The effect of anemia free club interventions to improve adolescent dietary intakes in Bandar Lampung City, Indonesia. *Open Access Macedonian Journal of Medical Sciences* **8**, 145–149.

**Eastern and Southern Africa**

1. Acham H, Kikafunda JK, Malde MK, et al. (2012) Breakfast, midday meals and academic achievement in rural primary schools in Uganda: implications for education and school health policy. *Food Nutr Res* **56**.

2. Adom T, Kengne AP, De Villiers A, et al. (2019) Prevalence of overweight and obesity among African primary school learners: a systematic review and meta-analysis. *Obesity Science and Practice* **5**, 487–502. Wiley-Blackwell.

3. Aiga H, Abe K, Andrianome VN, et al. (2019) Risk factors for malnutrition among school-aged children: a cross-sectional study in rural Madagascar. *BMC Public Health* **19**. BioMed Central Ltd.

4. Alelign T, Degarege A & Erko B (2015) Prevalence and factors associated with undernutrition and anaemia among school children in Durbete Town, northwest Ethiopia. *Archives of Public Health* **73**. BioMed Central Ltd.

5. Amare B, Moges B, Fantahun B, et al. (2012) Micronutrient levels and nutritional status of school children living in Northwest Ethiopia. *Nutrition Journal* **11**.

6. Anteneh ZA, Gedefaw M, Tekletsadek KN, et al. (2015) Risk factors of overweight and obesity among high school students in Bahir Dar City, north west Ethiopia: school based cross-sectional study. *Advances in Preventive Medicine* **2015**, 1–9. Hindawi Limited.

7. Arage G, Assefa M & Worku T (2019) Socio-demographic and economic factors are associated with nutritional status of adolescent school girls in Lay Guyint Woreda, Northwest Ethiopia. *SAGE Open Medicine* **7**, 205031211984467–205031211984467. SAGE Publications.

8. Armstrong MEG, Lambert MI & Lambert EV (2017) Relationships between different nutritional anthropometric statuses and health-related fitness of South African primary school children. *Annals of Human Biology* **44**, 208–213. Taylor and Francis Ltd.

9. Assey VD, Mgoba C, Mlingi N, et al. (2007) Remaining challenges in Tanzania’s efforts to eliminate iodine deficiency. *Public Health Nutrition* **10**, 1032–1038.

10. Barugahara EI, Kikafunda J & Gakenia WM (2013) Prevalence and risk factors of nutritional anaemia among female school children in Masindi District, Western Uganda. *African Journal of Food, Agriculture, Nutrition and Development* **13**.

11. Belachew T, Lindstrom D, Gebremariam A, et al. (2013) Food insecurity, food based coping strategies and suboptimal dietary practices of adolescents in Jimma Zone southwest Ethiopia. *PLoS One* **8**.

12. Belay E, Handebo S, Derso T, et al. (2019) Prevalence and determinants of pre-adolescent (5-14 years) acute and chronic undernutrition in Lay Armachiho District, Ethiopia. *International Journal for Equity in Health* **18**. BioMed Central Ltd.

13. Berg T, Magala-Nyago C & Iversen PO (2018) Nutritional status among adolescent girls in children’s homes: anthropometry and dietary patterns. *Clinical Nutrition* **37**, 926–933. Churchill Livingstone.

14. Birhanu M, Gedefaw L & Asres Y (2018) Anemia among school-age children: magnitude, severity and associated factors in Pawe Town, Benishangul-Gumuz Region, northwest Ethiopia. *Ethiopian journal of health sciences* **28**, 259–266.

15. Birru SM, Belew AK & Tariku A (2018) One in three adolescent schoolgirls in urban northwest Ethiopia is stunted. *Italian Journal of Pediatrics* **44**. BioMed Central Ltd.

16. Birru SM, Tariku A & Belew AK (2018) Improved dietary diversity of school adolescent girls in the context of urban northwest Ethiopia: 2017. *Italian journal of pediatrics* **44**, 48–48.

17. Bogale TY, Bala ET, Tadesse M, et al. (2018) Prevalence and associated factors for stunting among 6-12 years old school age children from rural community of Humbo district, Southern Ethiopia. *BMC Public Health* **18**. BioMed Central Ltd.

18. Bonney E, Ferguson G & Smits-Engelsman B (2018) Relationship between body mass index, cardiorespiratory and musculoskeletal fitness among south african adolescent girls. *International Journal of Environmental Research and Public Health* **15**. MDPI AG.

19. Brown C, Shaibu S, Maruapula S, et al. (2015) Perceptions and attitudes towards food choice in adolescents in Gaborone, Botswana. *Appetite* **95**, 29–35. Academic Press.

20. Chesere EJ, Orago ASS, Oteba LP, et al. (2008) Determinants of under nutrition among school age children in a nairobi peri-urban slum. *East African Medical Journal* **85**, 471–479.

21. Chirwa ED, Griffiths P, Maleta K, et al. (2014) Postnatal growth velocity and overweight in early adolescents: a comparison of rural and urban African boys and girls. *Am. J. Hum. Biol.* **26**, 643–651.

22. Chomba H, Martin HD & Kimywe J (2019) Prevalence and predictors of obesity among 7-to 17-year-old schoolchildren in urban Arusha, Tanzania. *Journal of Nutrition and Metabolism* **2019**. Hindawi Limited.

23. Choukem S-P, Tochie JN, Sibetcheu AT, et al. (2020) Overweight/obesity and associated cardiovascular risk factors in sub-Saharan African children and adolescents: a scoping review. *International Journal of Pediatric Endocrinology* **2020**. Springer Science and Business Media LLC.

24. Comandini O, Carmignani G, Cipriano A, et al. (2018) Cross-sectional and longitudinal analysis of nutritional status of school-children from Bumbire Island (United Republic of Tanzania). *American Journal of Human Biology* **30**. Wiley-Liss Inc.

25. Craig E, Reilly JJ & Bland R (2016) Risk factors for overweight and overfatness in rural South African children and adolescents. *J Public Health (Oxf)* **38**, 24–33.

26. Craig E, Reilly J & Bland R (2013) Body fatness or anthropometry for assessment of unhealthy weight status? Comparison between methods in South African children and adolescents. *Public Health Nutr* **16**, 2005–2013.

27. Daba KT, Gessesse GW & Sori SB (2020) Proportion of glaucoma among voluntary people coming for glaucoma screening program at Jimma University Department of Ophthalmology, Jimma, Ethiopia. *Ethiop J Health Sci* **30**, 13–22.

28. Danquah FI, Ansu-Mensah M, Bawontuo V, et al. (2020) Risk factors and morbidities associated with childhood obesity in sub-Saharan Africa: a systematic scoping review. *BMC Nutrition* **6**. BioMed Central.

29. Dansa R, Reta F, Mulualem D, et al. (2019) A nutrition education intervention to increase consumption of pulses showed improved nutritional status of adolescent girls in Halaba Special District, southern Ethiopia. *Ecology of Food and Nutrition* **58**, 353–365. Taylor and Francis Inc.

30. Darling AM, Sunguya B, Ismail A, et al. (2020) Gender differences in nutritional status, diet and physical activity among adolescents in eight countries in sub-Saharan Africa. *Trop Med Int Health* **25**, 33–43.

31. Degarege A, Hailemeskel E & Erko B (2015) Age-related factors influencing the occurrence of undernutrition in northeastern Ethiopia. *BMC Public Health* **15**. BioMed Central Ltd.

32. Demilew YM & Emiru AA (2018) Under nutrition and associated factors among school adolescents in Dangila Town, Northwest Ethiopia: a cross sectional study. *Afr Health Sci* **18**, 756–766.

33. Desalegn A, Mossie A & Gedefaw L (2014) Nutritional iron deficiency anemia: magnitude and its predictors among school age children, southwest ethiopia: a community based cross-sectional study. *PLoS ONE* **9**. Public Library of Science.

34. Desalew A, Mandesh A & Semahegn A (2017) Childhood overweight, obesity and associated factors among primary school children in dire dawa, eastern Ethiopia; a cross-sectional study. *BMC Obesity* **4**. BioMed Central Ltd.

35. Fall CHD, Abera M, Chopra H, et al. (2020) Anthropometric nutritional status, and social and dietary characteristics of African and Indian adolescents taking part in the TALENT (Transforming Adolescent Lives through Nutrition) qualitative study. *Public Health Nutrition*. Cambridge University Press.

36. Feeley A, Musenge E, Pettifor JM, et al. (2012) Changes in dietary habits and eating practices in adolescents living in urban South Africa: the Birth to Twenty cohort. *Nutrition* **28**, e1-6.

37. Friedman JF, Phillips-Howard PA, Mirel LB, et al. (2005) Progression of stunting and its predictors among school-aged children in western Kenya. *Eur J Clin Nutr* **59**, 914–922.

38. Gali N, Tamiru D & Tamrat M (2017) The emerging nutritional problems of school adolescents: overweight/obesity and associated factors in Jimma Town, Ethiopia. *Journal of Pediatric Nursing* **35**, 98–104. W.B. Saunders.

39. Gebregyorgis T, Tadesse T & Atenafu A (2016) Prevalence of thinness and stunting and associated factors among adolescent school girls in Adwa Town, north Ethiopia. *International Journal of Food Science* **2016**. Hindawi Limited.

40. Gedefaw L, Tesfaye M, Yemane T, et al. (2015) Anemia and iron deficiency among school adolescents: burden, severity, and determinant factors in southwest Ethiopia. *Adolescent Health, Medicine and Therapeutics*, 189–189. Dove Medical Press Ltd.

41. Getaneh Z, Enawgaw B, Engidaye G, et al. (2017) Prevalence of anemia and associated factors among school children in Gondar town public primary schools, northwest Ethiopia: A school-based cross-sectional study. *PLoS One* **12**, e0190151.

42. Ginsburg C, Griffiths PL, Richter LM, et al. (2013) Residential mobility, socioeconomic context and body mass index in a cohort of urban South African adolescents. *Health and Place* **19**, 99–107. Elsevier Ltd.

43. Girma K, Nibret E & Gedefaw M (2014) The status of iodine nutrition and iodine deficiency disorders among school children in Metekel Zone, northwest Ethiopia. *Ethiopian Journal of Health Sciences* **24**, 109. College of Public Health and Medical Sciences of Jimma University.

44. Gonete KA, Tariku A, Wami SD, et al. (2018) Prevalence and associated factors of anemia among adolescent girls attending high schools in Dembia District, northwest Ethiopia, 2017. *Archives of Public Health* **76**. BioMed Central Ltd.

45. Gonete KA, Tariku A, Wami SD, et al. (2020) Dietary diversity practice and associated factors among adolescent girls in Dembia district, northwest Ethiopia, 2017. *Public Health Reviews* **41**. BioMed Central Ltd.

46. Gwetu TP, Chhagan MK, Taylor M, et al. (2017) Anaemia control and the interpretation of biochemical tests for iron status in children. *BMC Research Notes* **10**. BioMed Central Ltd.

47. Gwetu TP, Taylor M, Chhagan M, et al. (2019) Health and educational achievement of school-aged children: the impact of anaemia and iron status on learning. *Health SA Gesondheid* **24**. AOSIS OpenJournals Publishing AOSIS (Pty) Ltd.

48. Hailu S, Wubshet M, Woldie H, et al. (2016) Iodine deficiency and associated factors among school children: a cross-sectional study in Ethiopia. *Archives of Public Health* **74**. BioMed Central Ltd.

49. Hall A, Kassa T, Demissie T, et al. (2008) National survey of the health and nutrition of schoolchildren in Ethiopia. *Tropical Medicine and International Health* **13**, 1518–1526.

50. Harika R, Faber M, Samuel F, et al. (2017) Are low intakes and deficiencies in iron, vitamin A, zinc, and iodine of public health concern in Ethiopian, Kenyan, Nigerian, and South African children and adolescents? *Food and Nutrition Bulletin* **38**, 405–427. SAGE Publications Inc.

51. Harika R, Faber M, Samuel F, et al. (2017) Micronutrient status and dietary intake of iron, vitamin A, iodine, folate and zinc in women of reproductive age and pregnant women in Ethiopia, Kenya, Nigeria and South Africa: a systematic review of data from 2005 to 2015. *Nutrients* **9**.

52. Hassen HY, Beyene M & Ali JH (2019) Dietary pattern and its association with iodine deficiency among school children in southwest Ethiopia; a cross-sectional study. *PLoS ONE* **14**. Public Library of Science.

53. Hassen K, Gizaw G & Belachew T (2017) Dual burden of malnutrition among adolescents of smallholder coffee farming households of Jimma Zone, southwest Ethiopia. *Food and Nutrition Bulletin* **38**, 196–208. SAGE Publications Inc.

54. Hauta-Alus HH, Korkalo L, Freese R, et al. (2018) Urban and rural dietary patterns are associated with anthropometric and biochemical indicators of nutritional status of adolescent Mozambican girls. *Public Health Nutrition* **21**, 1057–1064. Cambridge University Press.

55. Herrador Z, Perez-Formigo J, Sordo L, et al. (2015) Low dietary diversity and intake of animal source foods among school aged children in libo kemkem and fogera districts, Ethiopia. *PLoS ONE* **10**. Public Library of Science.

56. Horjus P, Aguayo VM, Roley JA, et al. (2005) School-based iron and folic acid supplementation for adolescent girls: findings from Manica Province, Mozambique. *Food and Nutrition Bulletin* **26**, 281–286. United Nations University Press.

57. Idamokoro M, Goon DT & Lyoka P (2019) Anthropometric and motor development characteristics of rural children in Nkonkobe municipality, South Africa. *Medicina dello Sport* **72**, 112–126. Edizioni Minerva Medica.

58. Irenso AA, Dessie Y, Berhane Y, et al. (2020) Prevalence and predictors of adolescent linear growth and stunting across the urban–rural gradient in eastern Ethiopia. *Tropical Medicine and International Health* **25**, 101–110. Blackwell Publishing Ltd.

59. Isabirye N, Bukenya JN, Nakafeero M, et al. (2020) Dietary diversity and associated factors among adolescents in eastern Uganda: a cross-sectional study. *BMC Public Health* **20**. BioMed Central Ltd.

60. Ismail A, Darling AM, Mosha D, et al. (2020) Prevalence and risk factors associated with malnutrition among adolescents in rural Tanzania. *Tropical Medicine and International Health* **25**, 89–100. Blackwell Publishing Ltd.

61. Jinabhai CC, Reddy P, Taylor M, et al. (2007) Sex differences in under and over nutrition among school-going black teenagers in South Africa: an uneven nutrition trajectory. *Tropical Medicine and International Health* **12**, 944–952.

62. Katungwe P, Mwangwela A & Geresomo N (2015) Dietary adequacy of rural school children among bambara groundnut growing farmers in Ntchisi district of Malawi. *African Journal of Food, Agriculture, Nutrition and Development* **15**, 9620–9634.

63. Kimani-Murage EW, Kahn K, Pettifor JM, et al. (2010) The prevalence of stunting, overweight and obesity, and metabolic disease risk in rural South African children. *BMC Public Health* **10**, 158.

64. Kiwanuka SN, Åstrøm AN & Trovik TA (2006) Sugar snack consumption in Ugandan schoolchildren; validity and reliability of a food frequency questionnaire. *Community Dentistry and Oral Epidemiology* **34**, 372–380.

65. Korkalo L, Freese R, Alfthan G, et al. (2015) Poor micronutrient intake and status is a public health problem among adolescent Mozambican girls. *Nutrition Research* **35**, 664–673. Elsevier Inc.

66. Kruger HS, Pretorius R & Schutte AE (2010) Stunting, adiposity, and low-grade inflammation in African adolescents from a township high school. *Nutrition* **26**, 90–99.

67. Kruger R, Kruger H & MacIntyre U (2006) The determinants of overweight and obesity among 10- to 15-year-old schoolchildren in the North West Province, South Africa – the THUSA BANA (Transition and Health during Urbanisation of South Africans; BANA, children) study. *Public Health Nutrition* **9**, 351–358. Cambridge University Press (CUP).

68. Leenstra T, Petersen LT, Kariuki SK, et al. (2005) Prevalence and severity of malnutrition and age at menarche; cross-sectional studies in adolescent schoolgirls in western Kenya. *European Journal of Clinical Nutrition* **59**, 41–48.

69. Mamabolo RL, Kruger HS, Lennox A, et al. (2007) Habitual physical activity and body composition of black township adolescents residing in the North West Province, South Africa. *Public Health Nutrition* **10**, 1047–1056.

70. Manyanga T, Barnes JD, Chaput JP, et al. (2020) Prevalence and correlates of objectively measured weight status among urban and rural Mozambican primary schoolchildren: a cross-sectional study. *PLoS ONE* **15**. Public Library of Science.

71. Manyanga T, El-Sayed H, Doku DT, et al. (2014) The prevalence of underweight, overweight, obesity and associated risk factors among school-going adolescents in seven African countries. *BMC Public Health* **14**, 887.

72. Maradzika J, Chapoterera B, Marume A, et al. (2017) Determinants of dietary patterns and obesity among secondary school adolescents in Harare, Zimbabwe, 2016. *International Journal of Child Health and Nutrition* **6**, 144–158. Lifescience Global.

73. Megersa DG, Mekonnen Abebe S, Abebe F, et al. (2017) Prevalence and associated factors of low serum zinc concentration in adolescents of Gambella city, southwest Ethiopia. *Nutrition and Dietary Supplements* **Volume 9**, 1–8. Dove Medical Press Ltd.

74. Meko LNM, Slabber-Stretch M, Walsh CM, et al. (2015) School environment, socioeconomic status and weight of children in Bloemfontein, South Africa. *Afr J Prim Health Care Fam Med* **7**.

75. Mekonnen T, Tariku A & Abebe SM (2018) Overweight/obesity among school aged children in Bahir Dar City: cross sectional study. *Italian Journal of Pediatrics* **44**. BioMed Central Ltd.

76. Melaku YA, Zello GA, Gill TK, et al. (2015) Prevalence and factors associated with stunting and thinness among adolescent students in Northern Ethiopia: A comparison to World Health Organization standards. *Archives of Public Health* **73**. BioMed Central Ltd.

77. Mitiku H, Admassu D, Teklemariam Z, et al. (2019) Nutritional status of school children in eastern Hararghe administrative zone, eastern Ethiopia. *Journal of Public Health (Germany)* **27**, 111–118. Springer Verlag.

78. Monyeki MA, Neetens R, Moss SJ, et al. (2012) The relationship between body composition and physical fitness in 14 year old adolescents residing within the Tlokwe local municipality, South Africa: the PAHL study. *BMC Public Health* **12**, 374.

79. Motswagole BS, Kruger HS, Faber M, et al. (2012) Body composition in stunted, compared to non-stunted, black South African children, from two rural communities. *South African Journal of Clinical Nutrition* **25**, 62–66.

80. Mpembeni RNM, Muhihi AJ, Muhihi AJ, et al. (2014) Overweight, obesity and perceptions about body weight among primary schoolchildren in dar es salaam, Tanzania. *Tanzania Journal of Health Research* **16**. National Institute for Medical Research.

81. Mphekgwana PM, Makgopa HM, Monyeki KD, et al. (2019) Ellisras longitudinal Study 2017: childhood underweight and blood pressure status in a rural black population of South Africa (ELS 26). *Cardiovascular Journal of Africa* **30**, 146–150. Clinics Cardive Publishing (PTY)Ltd.

82. Muktar M, Teji Roba K, Mengistie B, et al. (2018) Iodine deficiency and its associated factors among primary school children in Anchar district, eastern Ethiopia. *Pediatric Health, Medicine and Therapeutics* **Volume 9**, 89–95. Dove Medical Press Ltd.

83. Mulugeta A, Hagos F, Stoecker B, et al. (2009) Nutritional status of adolescent girls from rural communities of Tigray, northern Ethiopia. *Ethiopian Journal of Health Development* **23**.

84. Mushonga NGT, Kujinga P, Chagwena DT, et al. (2014) A restrospective study of the nutritional status of primary school children in Harare. *African Journal of Food, Agriculture, Nutrition and Development* **14**.

85. Muthuri SK, Wachira LJM, Onywera VO, et al. (2014) Correlates of objectively measured overweight/obesity and physical activity in Kenyan school children: Results from ISCOLE-Kenya. *BMC Public Health* **14**. BioMed Central Ltd.

86. Mwaikambo SA, Leyna GH, Killewo J, et al. (2015) Why are primary school children overweight and obese? A cross sectional study undertaken in Kinondoni district, Dar-es-salaam. *BMC Public Health* **15**. BioMed Central Ltd.

87. Napier C & Oldewage-Theron W (2015) Dietary intake and nutritional status of adolescent girls and young women in Durban, South Africa. *Journal of Consumer Sciences* **43**.

88. Negash S, Agyemang C, Matsha TE, et al. (2017) Differential prevalence and associations of overweight and obesity by gender and population group among school learners in South Africa: a cross-sectional study. *BMC Obesity* **4**. BioMed Central Ltd.

89. Neumann CG, Murphy SP, Gewa C, et al. (2007) Meat supplementation improves growth, cognitive, and behavioral outcomes in Kenyan children. *J Nutr* **137**, 1119–1123.

90. Ngwenya NA & Ramukumba TS (2017) Prevalence of adolescent obesity at a high school in the City of Tshwane. *Curationis* **40**.

91. Nicholaus C, Martin HD, Kassim N, et al. (2020) Dietary practices, nutrient adequacy, and nutrition status among adolescents in boarding high schools in the Kilimanjaro region, Tanzania. *Journal of Nutrition and Metabolism* **2020**. Hindawi Limited.

92. Ohnishi M, Leshabari S, Ambikile JS, et al. (2017) Associations among anthropometric measures, food consumption, and quality of life in school-age children in Tanzania. *Journal of Rural Medicine* **12**, 38–45. Japanese Association of Rural Medicine.

93. Okeyo AP, Seekoe E, de Villiers A, et al. (2020) Dietary practices and adolescent obesity in secondary school learners at disadvantaged schools in south africa: urban–rural and gender differences. *International Journal of Environmental Research and Public Health* **17**, 1–18. MDPI AG.

94. Otitoola O, Oldewage-Theron W & Egal A (2020) Prevalence of overweight and obesity among selected schoolchildren and adolescents in Cofimvaba, South Africa. *South African Journal of Clinical Nutrition*. Taylor and Francis Ltd.

95. Pangani IN, Kiplamai FK, Kamau JW, et al. (2016) Prevalence of overweight and obesity among primary school children aged 8–13 years in Dar es Salaam City, Tanzania. *Advances in Preventive Medicine* **2016**, 1–5. Hindawi Limited.

96. Parker ME, Mosites E, Reider K, et al. (2015) A blinded, cluster-randomized, placebo-controlled school feedingtrial in burundi using rice fortified with Iron, zinc, thiamine, and folic acid. *Food and Nutrition Bulletin* **36**, 481–492. SAGE Publications Inc.

97. Pienaar AE (2015) Prevalence of overweight and obesity among primary school children in a developing country: NW-CHILD longitudinal data of 6-9-yr-old children in South Africa. *BMC Obesity* **2**. BioMed Central Ltd.

98. Poopedi MA, Norris SA & Pettifor JM (2011) Factors influencing the vitamin D status of 10-year-old urban South African children. *Public Health Nutr* **14**, 334–339.

99. Pradeilles R, Griffiths PL, Norris SA, et al. (2015) Socio-economic influences on anthropometric status in urban South African adolescents: sex differences in the Birth to Twenty Plus cohort. *Public Health Nutr* **18**, 2998–3012.

100. Regasa RT & Haidar JA (2019) Anemia and its determinant of in-school adolescent girls from rural Ethiopia: a school based cross-sectional study. *BMC Women’s Health* **19**. BioMed Central Ltd.

101. Samuel FO, Egal AA, Oldewage-Theron WH, et al. (2010) Prevalence of zinc deficiency among primary school children in a poor peri-urban informal settlement in South Africa. *Health SA Gesondheid* **15**, 6.

102. Seal A, Kafwembe E, Kassim IAR, et al. (2008) Maize meal fortification is associated with improved vitamin a and iron status in adolescents and reduced childhood anaemia in a food aid-dependent refugee population. *Public Health Nutrition* **11**, 720–728.

103. Sebotsa MLD, Dannhauser A, Jooste PL, et al. (2005) Iodine status as determined by urinary iodine excretion in Lesotho two years after introducing legislation on universal salt iodization. *Nutrition* **21**, 20–24. Elsevier Inc.

104. Sedibe HM, Kahn K, Edin K, et al. (2014) Qualitative study exploring healthy eating practices and physical activity among adolescent girls in rural South Africa. *BMC Pediatr* **14**, 211.

105. Seyoum Y, Humblot C, Nicolas G, et al. (2019) Iron deficiency and anemia in adolescent girls consuming predominantly plant-based diets in rural Ethiopia. *Scientific Reports* **9**. Nature Research.

106. Shaka MF & Wondimagegne YA (2018) Anemia, a moderate public health concern among adolescents in south Ethiopia. *PLoS ONE* **13**. Public Library of Science.

107. Silva ABT, Capingana DP, Magalhães P, et al. (2016) Cardiovascular risk factors in pre-pubertal schoolchildren in Angola. *Cardiovascular Journal of Africa* **27**, 315–321. Clinics Cardive Publishing (PTY)Ltd.

108. Taklual W, Baye S, Mekie M, et al. (2020) Double burden of malnutrition among female adolescent students in Bahir Dar City, Amhara, Ethiopia. *BioMed Research International* **2020**. Hindawi Limited.

109. Talsma EF, Borgonjen-Van Den Berg KJ, Melse-Boonstra A, et al. (2018) The potential contribution of yellow cassava to dietary nutrient adequacy of primary-school children in Eastern Kenya; The use of linear programming. *Public Health Nutrition* **21**, 365–376. Cambridge University Press.

110. Tariku A, Belew AK, Gonete KA, et al. (2019) Stunting and its determinants among adolescent girls: findings from the Nutrition Surveillance Project, northwest Ethiopia. *Ecology of Food and Nutrition* **58**, 481–494. Taylor and Francis Inc.

111. Tariku EZ, Abebe GA, Melketsedik ZA, et al. (2018) Prevalence and factors associated with stunting and thinness among school-age children in Arba Minch Health and Demographic Surveillance Site, Southern Ethiopia. *PLoS One* **13**, e0206659.

112. Tatala SR, Kihamia CM, Kyungu LH, et al. (2008) Risk factors for anaemia in schoolchildren in Tanga Region, Tanzania. *Tanzania journal of health research* **10**, 189–202.

113. Tathiah N, Moodley I, Mubaiwa V, et al. (2013) South Africa’s nutritional transition: overweight, obesity, underweight and stunting in female primary school learners in rural KwaZulu-Natal, South Africa. *South African Medical Journal* **103**, 718–723.

114. Teferi DY, Atomssa GE & Mekonnen TC (2018) Overweight and undernutrition in the cases of school-going adolescents in Wolaita Sodo Town, southern Ethiopia: cross-sectional study. *Journal of Nutrition and Metabolism* **2018**. Hindawi Limited.

115. Teji K, Dessie Y, Assebe T, et al. (2016) Anaemia and nutritional status of adolescent girls in Babile District, Eastern Ethiopia. *Pan African Medical Journal* **24**. African Field Epidemiology Network.

116. Temple NJ, Steyn NP, Myburgh NG, et al. (2006) Food items consumed by students attending schools in different socioeconomic areas in Cape Town, South Africa. *Nutrition* **22**, 252–258.

117. Toriola OO, Monyeki MA & Toriola AL (2015) Two-year longitudinal health-related fitness, anthropometry and body composition status amongst adolescents in Tlokwe Municipality: The PAHL Study. *Afr J Prim Health Care Fam Med* **7**, 896.

118. Turyashemererwa FM, Kikafunda J, Annan R, et al. (2013) Dietary patterns, anthropometric status, prevalence and risk factors for anaemia among school children aged 5-11 years in central Uganda. *Journal of Human Nutrition and Dietetics* **26**, 73–81.

119. van den Berg VL, Seheri L & Raubenheimer J (2014) Body mass index of 16-year olds in urban Maseru, Lesotho. *African Journal of Primary Health Care and Family Medicine* **6**. AOSIS OpenJournals Publishing AOSIS (Pty) Ltd.

120. Van Der Hoeven M, Faber M, Osei J, et al. (2016) Effect of African leafy vegetables on the micronutrient status of mildly deficient farm-school children in South Africa: a randomized controlled study. *Public Health Nutrition* **19**, 935–945. Cambridge University Press.

121. Visser M, Van Zyl T, Hanekom SM, et al. (2019) Nutrient patterns and their relation to anemia and iron status in 5- to 12-y-old children in South Africa. *Nutrition* **62**, 194–200. Elsevier Inc.

122. Voorend CGN, Norris SA, Griffiths PL, et al. (2013) ‘We eat together; today she buys, tomorrow I will buy the food’: adolescent best friends’ food choices and dietary practices in Soweto, South Africa. *Public Health Nutr* **16**, 559–567.

123. Wakayo T, Belachew T, Vatanparast H, et al. (2015) Vitamin D deficiency and its predictors in a country with thirteen months of sunshine: the case of school children in Central Ethiopia. *PLoS ONE* **10**. Public Library of Science.

124. Wei D, Brigell R, Khadka A, et al. (2019) Comprehensive school-based health programs to improve child and adolescent health: evidence from Zambia. *PLoS ONE* **14**. Public Library of Science.

125. White Z, White S, Dalvie T, et al. (2019) Bone health, body composition, and vitamin D status of black preadolescent children in South Africa. *Nutrients* **11**. MDPI AG.

126. Wolde T & Belachew T (2019) Predictors of thinness and improved dietary diversity among school aged children in Southern Ethiopia. *Nutrition and Dietary Supplements* **Volume 11**, 49–58. Informa UK Limited.

127. Woodruff BA, Blanck HM, Slutsker L, et al. (2006) Anaemia, iron status and vitamin A deficiency among adolescent refugees in Kenya and Nepal. *Public Health Nutrition* **9**, 26–34. Cambridge University Press (CUP).

128. Wrottesley SV, Bosire EN, Mukoma G, et al. (2019) Age and gender influence healthy eating and physical activity behaviours in South African adolescents and their caregivers: Transforming Adolescent Lives through Nutrition Initiative (TALENT). *Public Health Nutr*, 1–20.

129. Wrottesley SV, Pedro TM, Fall CH, et al. (2019) A review of adolescent nutrition in South Africa: transforming adolescent lives through nutrition initiative. *South African Journal of Clinical Nutrition* **0**, 1–39.

130. Zemene MA, Engidaw MT, Gebremariam AD, et al. (2019) Nutritional status and associated factors among high school adolescents in Debre Tabor Town, South Gondar Zone, northcentral Ethiopia. *BMC Nutrition* **5**. Springer Science and Business Media LLC.

**Europe and Central Asia**

1. Agirbasli M, Tanrikulu B, Arikan S, et al. (2008) Trends in body mass index, blood pressure and parental smoking habits in middle socio-economic level Turkish adolescents. *Journal of human hypertension* **22**, 2007/07/06 ed., 12–7.

2. Ahluwalia N, Dalmasso P, Rasmussen M, et al. (2015) Trends in overweight prevalence among 11-, 13- and 15-year-olds in 25 countries in Europe, Canada and USA from 2002 to 2010. *European journal of public health* **25 Suppl 2**, 2015/03/26 ed., 28–32.

3. Akbulut G, Yildirim M, Sanlier N, et al. (2014) Comparison of energy balance-related behaviours and measures of body composition between Turkish adolescents in Turkey and Turkish immigrant adolescents in the Netherlands. *Public health nutrition* **17**, 2014/01/31 ed., 2692–9.

4. Akdemir M, Donmez L & Polat H (2017) The effect of nutritional and physical activity interventions on nutritional status and obesity in primary school children: a cluster randomized controlled study. *The Kuwait medical journal* **49**, 105‐113.

5. Akis N, Pala K, Meric-Utku A, et al. (2009) Hypertension in children (12-14 years) - a case-control study in Bursa, Turkey. *The Turkish journal of pediatrics* **51**, 2010/02/02 ed., 437–43.

6. Akman AO, Tumer L, Hasanoglu A, et al. (2011) Frequency of vitamin D insufficiency in healthy children between 1 and 16 years of age in Turkey. *Pediatrics international : official journal of the Japan Pediatric Society* **53**, 2011/10/13 ed., 968–73.

7. Akman M, Akan H, Izbirak G, et al. (2010) Eating patterns of Turkish adolescents: a cross-sectional survey. *Nutrition journal* **9**, 2010/12/21 ed., 67.

8. Alper Z, Ercan I & Uncu Y (2018) A meta-analysis and an evaluation of trends in obesity prevalence among children and adolescents in Turkey: 1990 through 2015. *Journal of clinical research in pediatric endocrinology* **10**, 2017/09/14 ed., 59–67.

9. Andiran N, Celik N, Akca H, et al. (2012) Vitamin D deficiency in children and adolescents. *Journal of clinical research in pediatric endocrinology* **4**, 2012/03/08 ed., 25–9.

10. Ardic A & Erdogan S (2017) The effectiveness of the COPE healthy lifestyles TEEN program: a school-based intervention in middle school adolescents with 12-month follow-up. *Journal of advanced nursing* **73**, 2016/11/24 ed., 1377–1389.

11. Aypak C, Turedi O & Yuce A (2014) The association of vitamin D status with cardiometabolic risk factors, obesity and puberty in children. *European journal of pediatrics* **173**, 2013/10/18 ed., 367–73.

12. Ayranci U, Erenoglu N & Son O (2010) Eating habits, lifestyle factors, and body weight status among Turkish private educational institution students. *Nutrition (Burbank, Los Angeles County, Calif.)* **26**, 2009/11/17 ed., 772–8.

13. Bas M, Altan T, Dincer D, et al. (2005) Determination of dietary habits as a risk factor of cardiovascular heart disease in Turkish adolescents. *European journal of nutrition* **44**, 2004/08/17 ed., 174–82.

14. Bas M & Kiziltan G (2007) Relations among weight control behaviors and eating attitudes, social physique anxiety, and fruit and vegetable consumption in Turkish adolescents. *Adolescence* **42**, 2007/06/01 ed., 167–78.

15. Bilic-Kirin V, Burazin J, Buljan V, et al. (2013) Influence of health education regarding correct diet on anthropometric indexes in children. *Collegium antropologicum* **37**, 2014/03/13 ed., 1089–94.

16. Bodzsar EB & Zsakai A (2014) Recent trends in childhood obesity and overweight in the transition countries of eastern and central Europe. *Annals of human biology* **41**, 2014/04/08 ed., 263–70.

17. Bojadzieva Stojanoska B, Nakeva Janevska N, Matveeva N, et al. (2014) Growth parameters and nutritional status in relation to socioeconomic status of Macedonian adolescents. *Prilozi (Makedonska akademija na naukite i umetnostite. Oddelenie za medicinski nauki)* **35**, 2014/05/07 ed., 189–97.

18. Budak N, Bayram F, Gunay O, et al. (2007) Iodine deficiency: an important and severe public health problem in Kayseri, Central Anatolia. *Journal of endocrinological investigation* **30**, 2008/02/06 ed., 920–4.

19. Budak N, Ozturk A, Mazicioglu M, et al. (2010) Decreased high-density lipoprotein cholesterol and insulin resistance were the most common criteria in 12- to 19-year-old adolescents. *European journal of nutrition* **49**, 2009/10/31 ed., 219–25.

20. Can HO, Ceber E, Sogukpinar N, et al. (2008) Eating habits, knowledge about cancer prevention and the HPLP scale in Turkish adolescents. *Asian Pacific journal of cancer prevention : APJCP* **9**, 2009/03/05 ed., 569–74.

21. Canpolat BI, Orsel S, Akdemir A, et al. (2005) The relationship between dieting and body image, body ideal, self-perception, and body mass index in Turkish adolescents. *The International journal of eating disorders* **37**, 2005/02/26 ed., 150–5.

22. Chirita-Emandi A, Barbu CG, Cinteza EE, et al. (2016) Overweight and underweight prevalence trends in children from Romania - pooled analysis of cross-sectional studies between 2006 and 2015. *Obesity facts* **9**, 2016/06/20 ed., 206–20.

23. Chirita-Emandi A, Puiu M, Gafencu M, et al. (2013) Arterial hypertension in school-aged children in western Romania. *Cardiology in the young* **23**, 2012/07/17 ed., 189–96.

24. Chirita-Emandi A, Socolov D, Haivas C, et al. (2015) Vitamin D status: a different story in the very young versus the very old Romanian patients. *PloS one* **10**, 2015/05/30 ed., e0128010.

25. Cicek B, Ozturk A, Mazicioglu MM, et al. (2009) The risk analysis of arm fat area in Turkish children and adolescents. *Annals of human biology* **36**, 2008/12/03 ed., 28–37.

26. Cinar AB & Murtomaa H (2011) Interrelation between obesity, oral health and life-style factors among Turkish school children. *Clinical oral investigations* **15**, 2010/01/08 ed., 177–84.

27. Cinar B & Murtomaa H (2008) Clustering of obesity and dental health with lifestyle factors among Turkish and Finnish pre-adolescents. *Obesity facts* **1**, 2008/01/01 ed., 196–202.

28. Crncevic-Orlic Z, Ruzic A, Rajkovic K, et al. (2005) The effectiveness of a 40-year long iodine prophylaxis in endemic goitre region of Grobnik, Croatia. *Collegium antropologicum* **29**, 2006/01/19 ed., 509–13.

29. Demirci H, Nuhoglu C, Ursavas IS, et al. (2013) Obesity and asymptomatic hypertension among children aged 6-13 years living in Bursa, Turkey. *Family practice* **30**, 2013/09/27 ed., 629–33.

30. Dinc G, Saatli G, Baydur H, et al. (2009) Hypertension and overweight among Turkish adolescents in a city in Aegean region of Turkey: a strong relationship in a population with a relatively low prevalence of overweight. *Anadolu kardiyoloji dergisi : AKD = the Anatolian journal of cardiology* **9**, 2009/12/08 ed., 450–6.

31. Discigil G, Tekin N & Soylemez A (2009) Obesity in Turkish children and adolescents: prevalence and non-nutritional correlates in an urban sample. *Child: care, health and development* **35**, 2009/02/21 ed., 153–8.

32. Djordjevic-Nikic M & Dopsaj M (2013) Characteristics of eating habits and physical activity in relation to body mass index among adolescents. *Journal of the American College of Nutrition* **32**, 2013/09/13 ed., 224–33.

33. Djordjevic-Nikic M, Dopsaj M & Veskovic A (2013) Nutritional and physical activity behaviours and habits in adolescent population of Belgrade. *Vojnosanitetski pregled* **70**, 2013/07/28 ed., 548–54.

34. Djordjic V, Radisavljevic S, Milanovic I, et al. (2016) WHO European Childhood Obesity Surveillance Initiative in Serbia: a prevalence of overweight and obesity among 6-9-year-old school children. *Journal of pediatric endocrinology & metabolism : JPEM* **29**, 2016/08/22 ed., 1025–30.

35. Due P, Damsgaard MT, Rasmussen M, et al. (2009) Socioeconomic position, macroeconomic environment and overweight among adolescents in 35 countries. *International journal of obesity (2005)* **33**, 2009/07/22 ed., 1084–93.

36. Dundar C & Oz H (2012) Obesity-related factors in Turkish school children. *TheScientificWorldJournal* **2012**, 2012/05/02 ed., 353485.

37. Duyar I & Ozener B (2005) Growth and nutritional status of male adolescent laborers in Ankara, Turkey. *American journal of physical anthropology* **128**, 2005/05/17 ed., 693–8.

38. Ercan S, Dallar YB, Onen S, et al. (2012) Prevalence of obesity and associated risk factors among adolescents in Ankara, Turkey. *Journal of clinical research in pediatric endocrinology* **4**, 2012/11/15 ed., 204–7.

39. Erenoglu N, Ayranci U & Son O (2006) Eating habits reported by secondary school students in a city of west Turkey. *Eating behaviors* **7**, 2006/10/24 ed., 348–54.

40. Erol M, Yigit O, Kucuk SH, et al. (2015) Vitamin D deficiency in children and adolescents in Bagcilar, Istanbul. *Journal of clinical research in pediatric endocrinology* **7**, 2015/09/01 ed., 134–9.

41. Ersoy B, Gunes HS, Gunay T, et al. (2006) Interaction of two public health problems in Turkish schoolchildren: nutritional deficiencies and goitre. *Public health nutrition* **9**, 2006/11/28 ed., 1001–6.

42. Etiler N, Cizmecioglu FM, Hatun S, et al. (2011) Nutritional status of students in Kocaeli, Turkey: a population-based study. *Pediatrics international : official journal of the Japan Pediatric Society* **53**, 2010/07/16 ed., 231–5.

43. Facchini F, Fiori G, Bedogni G, et al. (2007) Prevalence of overweight and cardiovascular risk factors in rural and urban children from central Asia: the Kazakhstan health and nutrition examination survey. *American journal of human biology : the official journal of the Human Biology Council* **19**, 2007/08/19 ed., 809–20.

44. Franzellin F, Hyska J, Bushi E, et al. (2009) A national study of iodine status in Albania. *Journal of endocrinological investigation* **32**, 2009/05/29 ed., 533–7.

45. Garipagaoglu M, Budak N, Sut N, et al. (2009) Obesity risk factors in Turkish children. *Journal of pediatric nursing* **24**, 2009/07/28 ed., 332–7.

46. Garipagaoglu M, Oner N, Vatansever U, et al. (2008) Dietary intakes of adolescents living in Edirne, Turkey. *Journal of the American College of Nutrition* **27**, 2008/10/08 ed., 394–400.

47. Gaspar T, de Matos MG, Luszczynska A, et al. (2014) The impact of a rural or urban context in eating awareness and self-regulation strategies in children and adolescents from eight European countries. *International journal of psychology : Journal international de psychologie* **49**, 2014/05/14 ed., 158–66.

48. Gatseva PD & Argirova MD (2009) Benefits and risks from the national strategy for improvement of iodine nutrition in Bulgaria: study on schoolchildren living in a rural area. *Public health* **123**, 2009/05/19 ed., 456–8.

49. Gatseva P, Vladeva S & Argirova M (2007) Evaluation of endemic goiter prevalence in Bulgarian schoolchildren: results from national strategies for prevention and control of iodine-deficiency disorders. *Biological trace element research* **116**, 2007/08/22 ed., 273–8.

50. Gerasimov GA, Haar F van der & Lazarus JH (2018) Overview of iodine deficiency prevention strategies in the south-eastern Europe and central Asia region: 2009–2016. *Clinical and experimental thyroidology* **13**, 16–22.

51. Giannopoulou D, Grammatikopoulou MG, Poulimeneas D, et al. (2017) Nutritional surveillance of Christian Orthodox minority adolescents in Istanbul. *Journal of immigrant and minority health* **19**, 2016/04/09 ed., 333–340.

52. Giese H, Konig LM, Taut D, et al. (2015) Exploring the association between television advertising of healthy and unhealthy foods, self-control, and food intake in three European countries. *Applied psychology. Health and well-being* **7**, 2014/11/05 ed., 41–62.

53. Gontarev S, Kalac R, Velickovska L, et al. (2018) Health-related physical fitness of normal, stunted and overweight children aged 6-14 years in Macedonia. *Nutricion hospitalaria* **35**, 2018/10/12 ed., 1208–1214.

54. Gontarev S, Kalac R, Zivkovic V, et al. (2017) The association between high blood pressure, physical fitness and fatness in adolescents. *Nutricion hospitalaria* **34**, 2017/03/01 ed., 35–40.

55. Gundogdu Z (2008) Relationship between BMI and blood pressure in girls and boys. *Public health nutrition* **11**, 2008/04/23 ed., 1085–8.

56. Gur E, Can G, Akkus S, et al. (2006) Is undernutrition a problem among Turkish school children? Which factors have an influence on it? *Journal of tropical pediatrics* **52**, 2006/07/28 ed., 421–6.

57. Gur E, Yildiz I, Celkan T, et al. (2005) Prevalence of anemia and the risk factors among schoolchildren in Istanbul. *Journal of tropical pediatrics* **51**, 2005/08/30 ed., 346–50.

58. Gurinović M, Kadvan A, Vukotić M, et al. (2011) The quality of nutrition in schoolchildren and adult members of families. In *Yugoslav study of atherosclerosis precursors in schoolchildren in Serbia: twenty years follow-up*, pp. 343–368 [Nedeljković S, editor]. Belgrade, Serbia: Medical Faculty University of Belgrade.

59. Hasanbegovic S, Mesihovic-Dinarevic S, Cuplov M, et al. (2010) Epidemiology and etiology of obesity in children and youth of Sarajevo Canton. *Bosnian journal of basic medical sciences* **10**, 2010/05/29 ed., 140–6.

60. Hashizume M, Chiba M, Shinohara A, et al. (2005) Anaemia, iron deficiency and vitamin A status among school-aged children in rural Kazakhstan. *Public health nutrition* **8**, 2005/10/21 ed., 564–71.

61. Hatipoglu N, Kurtoglu S, Ozturk A, et al. (2009) The weight and height percentiles in 6-18 year old children in Kayseri and comparison with Istanbul data. *Journal of clinical research in pediatric endocrinology* **1**, 2009/03/01 ed., 129–35.

62. Hatun S, Islam O, Cizmecioglu F, et al. (2005) Subclinical vitamin D deficiency is increased in adolescent girls who wear concealing clothing. *The Journal of nutrition* **135**, 2005/01/27 ed., 218–22.

63. Janevic T, Petrovic O, Bjelic I, et al. (2010) Risk factors for childhood malnutrition in Roma settlements in Serbia. *BMC public health* **10**, 2010/08/24 ed., 509.

64. Jukic T, Zimmermann MB, Granic R, et al. (2015) Sufficient iodine intake in schoolchildren from the Zagreb area: assessment with dried blood spot thyroglobulin as a new functional biomarker for iodine deficiency. *Acta clinica Croatica* **54**, 2016/03/29 ed., 424–31.

65. Kaya M, Sayan A, Birinci M, et al. (2014) The obesity prevalence among students between the ages of 5 and 19 in Kutahya. *Turk J Med Sci* **44**, 10–15.

66. Keskin Y, Moschonis G, Dimitriou M, et al. (2005) Prevalence of iron deficiency among schoolchildren of different socio-economic status in urban Turkey. *Eur J Clin Nutr* **59**, 64–71.

67. Kherkheulidze M, Kavlashvili N, Kandelaki E, et al. (2012) Evaluation of nutritional knowledge of second grade school children and assessment of their dietary intake. *Georgian Med News*, 58–64.

68. Koca T, Akcam M, Serdaroglu F, et al. (2017) Breakfast habits, dairy product consumption, physical activity, and their associations with body mass index in children aged 6-18. *European journal of pediatrics* **176**, 2017/08/12 ed., 1251–1257.

69. Kocaoglu B, Moschonis G, Dimitriou M, et al. (2005) Parental educational level and cardiovascular disease risk factors in schoolchildren in large urban areas of Turkey: directions for public health policy. *BMC public health* **5**, 2005/02/08 ed., 13.

70. Koksal E, Tekcicek M, Yalcin SS, et al. (2011) Association between anthropometric measurements and dental caries in Turkish school children. *Central European journal of public health* **19**, 2011/10/27 ed., 147–51.

71. Kukulu K, Sarvan S, Muslu L, et al. (2010) Dietary habits, economic status, academic performance and body mass index in school children: a comparative study. *Journal of child health care : for professionals working with children in the hospital and community* **14**, 2010/11/17 ed., 355–66.

72. Kurdak H, Bozdemir N, Saatci E, et al. (2010) Self-perceived body weight status and weight-control behaviors of high school students in a southern city of Turkey. *Collegium antropologicum* **34**, 2011/08/31 ed., 1295–302.

73. Kurtoglu S, Mazicioglu MM, Ozturk A, et al. (2010) Body fat reference curves for healthy Turkish children and adolescents. *European journal of pediatrics* **169**, 2010/05/28 ed., 1329–35.

74. Kutlu R, Karakose S, Gungor K, et al. (2011) The goiter prevalence and urinary iodine levels among adolescents. *The Turkish journal of pediatrics* **53**, 2011/08/23 ed., 161–8.

75. Lazzeri G, Ahluwalia N, Niclasen B, et al. (2016) Trends from 2002 to 2010 in daily breakfast consumption and its socio-demographic correlates in adolescents across 31 countries participating in the HBSC study. *PloS one* **11**, 2016/03/31 ed., e0151052.

76. Limnili G, Ozcakar N & Kartal M (2016) Health promotion lifestyle profile scores are not associatedwith obesity in high school students. *Turkish journal of medical sciences* **46**, 2016/08/12 ed., 1018–24.

77. Lotrean LM & Tutui I (2015) Individual and familial factors associated with fruit and vegetable intake among 11- to 14-year-old Romanian school children. *Health & social care in the community* **23**, 2014/10/18 ed., 541–9.

78. Manios Y, Kolotourou M, Moschonis G, et al. (2005) Macronutrient intake, physical activity, serum lipids and increased body weight in primary schoolchildren in Istanbul. *Pediatrics international : official journal of the Japan Pediatric Society* **47**, 2005/03/18 ed., 159–66.

79. Manios Y, Moschonis G, Kolotourou M, et al. (2007) Iron deficiency prevalence and dietary patterns by school district in Istanbul. *Journal of human nutrition and dietetics : the official journal of the British Dietetic Association* **20**, 2007/11/16 ed., 549–57.

80. Martinovic M, Belojevic G, Evans GW, et al. (2015) Prevalence of and contributing factors for overweight and obesity among Montenegrin schoolchildren. *European journal of public health* **25**, 2015/04/07 ed., 833–9.

81. Mazicioglu MM, Yalcin BM, Ozturk A, et al. (2010) Anthropometric risk factors for elevated blood pressure in adolescents in Turkey aged 11-17. *Pediatric nephrology (Berlin, Germany)* **25**, 2010/08/20 ed., 2327–34.

82. Melkevik O, Haug E, Rasmussen M, et al. (2015) Are associations between electronic media use and BMI different across levels of physical activity? *BMC public health* **15**, 2015/05/20 ed., 497.

83. Milanovic I, Radisavljevic-Janic S, Zivkovic MZ, et al. (2019) Health-related physical fitness levels and prevalence of obesity in Serbian elementary schoolchildren. *Nutr Hosp* **36**, 253–260.

84. Mladenova S & Andreenko E (2015) Prevalence of underweight, overweight, general and central obesity among 8-15-years old Bulgarian children and adolescents (Smolyan region, 2012-2014). *Nutricion hospitalaria* **31**, 2015/06/05 ed., 2419–27.

85. Mocanu V (2013) Prevalence of overweight and obesity in urban elementary school children in northeastern Romania: its relationship with socioeconomic status and associated dietary and lifestyle factors. *BioMed research international* **2013**, 2013/08/13 ed., 537451.

86. Ng M, Fleming T, Robinson M, et al. (2014) Global, regional, and national prevalence of overweight and obesity in children and adults during 1980-2013: a systematic analysis for the Global Burden of Disease Study 2013. *Lancet (London, England)* **384**, 2014/06/02 ed., 766–81.

87. Nisanci Kilinc F & Cagdas DN (2013) Diet and physical activity interventions do have effects on body composition and metabolic syndrome parameters in overweight and obese adolescents and their mothers. *The Turkish journal of pediatrics* **55**, 2013/11/13 ed., 292–9.

88. Olaya B, Moneta MV, Pez O, et al. (2015) Country-level and individual correlates of overweight and obesity among primary school children: a cross-sectional study in seven European countries. *BMC public health* **15**, 2015/05/09 ed., 475.

89. Olmez D, Bober E, Buyukgebiz A, et al. (2006) The frequency of vitamin D insufficiency in healthy female adolescents. *Acta paediatrica (Oslo, Norway : 1992)* **95**, 2006/09/20 ed., 1266–9.

90. Ozguven I, Ersoy B, Ozguven AA, et al. (2010) Evaluation of nutritional status in Turkish adolescents as related to gender and socioeconomic status. *Journal of clinical research in pediatric endocrinology* **2**, 2011/01/29 ed., 111–6.

91. Pirincci E, Durmus B, Gundogdu C, et al. (2010) Prevalence and risk factors of overweight and obesity among urban school children in Elazig city, Eastern Turkey, 2007. *Annals of human biology* **37**, 2009/12/09 ed., 44–56.

92. Pokrajac-Bulian A, Zivcić-Becirević I, Calugi S, et al. (2006) School prevention program for eating disorders in Croatia: a controlled study with six months of follow-up. *Eating and weight disorders* **11**, 171‐178.

93. Radisavljevi S, Milanovi I, Živkovi M, et al. (2013) Prevalence of overweight and obesity among Belgrade youth: a study in a representative sample of 9–14-year-old children and adolescents. *Anthropological Notebooks*, 10.

94. Radmanovic-Burgic M, Gavric Z & Burgic S (2011) Eating attitudes in adolescent girls. *Psychiatria Danubina* **23**, 2011/03/31 ed., 64–8.

95. Rakic R, Pavlica T & Jovicic D (2016) Overweight and obesity in children and adolescents from Serbia in the period 2001-2004 and 2011-2014. *Anthropologischer Anzeiger; Bericht uber die biologisch-anthropologische Literatur* **73**, 2016/03/24 ed.

96. Sahingoz SA & Sanlier N (2011) Compliance with Mediterranean Diet Quality Index (KIDMED) and nutrition knowledge levels in adolescents. A case study from Turkey. *Appetite* **57**, 2011/06/01 ed., 272–7.

97. Senol V, Unalan D, Bayat M, et al. (2014) Change in reference body mass index percentiles and deviation in overweight and obesity over 3 years in Turkish children and adolescents. *Journal of pediatric endocrinology & metabolism : JPEM* **27**, 2014/07/11 ed., 1121–9.

98. Simsek E, Akpinar S, Bahcebasi T, et al. (2008) The prevalence of overweight and obese children aged 6-17 years in the West Black Sea region of Turkey. *International journal of clinical practice* **62**, 2007/11/21 ed., 1033–8.

99. Sokolovic S, Alimanovic-Alagic R, Dzananovic L, et al. (2017) Vitamin D status in Bosnia and Herzegovina: the cross-sectional epidemiological analysis. *Osteoporosis international : a journal established as result of cooperation between the European Foundation for Osteoporosis and the National Osteoporosis Foundation of the USA* **28**, 2016/11/28 ed., 1021–1025.

100. Soric M, Jembrek Gostovic M, Gostovic M, et al. (2014) Tracking of BMI, fatness and cardiorespiratory fitness from adolescence to middle adulthood: the Zagreb Growth and Development Longitudinal Study. *Annals of human biology* **41**, 2013/11/10 ed., 238–43.

101. Soyer MT, Ergin I & Gursoy ST (2008) Effects of social determinants on food choice and skipping meals among Turkish adolescents. *Asia Pacific journal of clinical nutrition* **17**, 2008/07/01 ed., 208–15.

102. Spiroski I, Dimitrovska Z, Gjorgjev D, et al. (2011) Nutritional status and growth parameters of school-age Roma children in the Republic of Macedonia. *Central European journal of public health* **19**, 2011/07/12 ed., 102–7.

103. Sujoldzic A & De Lucia A (2007) A cross-cultural study of adolescents--BMI, body image and psychological well-being. *Collegium antropologicum* **31**, 2007/06/30 ed., 123–30.

104. Tahirovic H, Imsiragic-Zovko S, Toromanovic A, et al. (2007) Assessment of the success of implementation of new rule book on salt iodination in Federation of Bosnia and Herzegovina. *Journal of endocrinological investigation* **30**, 2007/02/24 ed., 9–12.

105. Tazhibayev S, Dolmatova O, Ganiyeva G, et al. (2008) Evaluation of the potential effectiveness of wheat flour and salt fortification programs in five Central Asian countries and Mongolia, 2002-2007. *Food and nutrition bulletin* **29**, 2009/02/21 ed., 255–65.

106. Tishukaj F, Shalaj I, Gjaka M, et al. (2017) Physical fitness and anthropometric characteristics among adolescents living in urban or rural areas of Kosovo. *BMC public health* **17**, 2017/09/17 ed., 711.

107. Topcu S, Simsek Orhon F, Ulukol B, et al. (2017) Secular trends in height, weight and body mass index of primary school children in Turkey between 1993 and 2016. *Journal of pediatric endocrinology & metabolism : JPEM* **30**, 2017/10/19 ed., 1177–1186.

108. Toruner EK & Savaser S (2010) A controlled evaluation of a school-based obesity prevention in Turkish school children. *The Journal of school nursing : the official publication of the National Association of School Nurses* **26**, 2010/09/25 ed., 473–82.

109. Tsitsika AK, Andrie EK, Psaltopoulou T, et al. (2016) Association between problematic internet use, socio-demographic variables and obesity among European adolescents. *European journal of public health* **26**, 2016/04/27 ed., 617–22.

110. Turan S, Bereket A, Furman A, et al. (2007) The effect of economic status on height, insulin-like growth factor (IGF)-I and IGF binding protein-3 concentrations in healthy Turkish children. *European journal of clinical nutrition* **61**, 2006/12/14 ed., 752–8.

111. Turkkahraman D, Bircan I, Tosun O, et al. (2006) Prevalence and risk factors of obesity in school children in Antalya, Turkey. *Saudi medical journal* **27**, 2006/07/11 ed., 1028–33.

112. Ucar B, Kilic Z, Dinleyici EC, et al. (2005) Body mass index values in Turkish schoolchildren aged 7-18 years. *Journal of tropical pediatrics* **51**, 2005/06/17 ed., 258–60.

113. Unalan D, Senol V, Bayat M, et al. (2013) Change in waist circumference over 3 years in Turkish children and adolescents. *Annals of human biology* **40**, 2013/05/23 ed., 419–25.

114. Ursoniu S, Putnoky S & Vlaicu B (2011) Body weight perception among high school students and its influence on weight management behaviors in normal weight students: a cross-sectional study. *Wiener klinische Wochenschrift* **123**, 2011/05/19 ed., 327–33.

115. Uzun H, Gozkaya S, Yesildal N, et al. (2014) The prevalence of goiter and hypothyroidism among school children 6 years after introduction of a mandatory salt iodination program in a severely iodine-deficient area of the West Black Sea region of Turkey. *Journal of tropical pediatrics* **60**, 2014/02/13 ed., 318–21.

116. Vereecken CA, Todd J, Roberts C, et al. (2006) Television viewing behaviour and associations with food habits in different countries. *Public health nutrition* **9**, 2006/03/31 ed., 244–50.

117. Vuralli D, Tumer L, Hasanoglu A, et al. (2014) Vitamin A status and factors associated in healthy school-age children. *Clinical nutrition (Edinburgh, Scotland)* **33**, 2013/08/07 ed., 509–12.

118. Vuralli D, Tumer L & Hasanoglu A (2017) Zinc deficiency in the pediatric age group is common but underevaluated. *World journal of pediatrics : WJP* **13**, 2017/01/20 ed., 360–366.

119. Wirth JP, Rajabov T, Petry N, et al. (2018) Micronutrient deficiencies, over- and undernutrition, and their contribution to anemia in Azerbaijani preschool children and non-pregnant women of reproductive age. *Nutrients* **10**, 2018/10/14 ed.

120. Yuca SA, Yilmaz C, Cesur Y, et al. (2010) Prevalence of overweight and obesity in children and adolescents in eastern Turkey. *Journal of clinical research in pediatric endocrinology* **2**, 2011/01/29 ed., 159–63.

121. Zaborskis A, Petronyte G, Sumskas L, et al. (2008) Body image and weight control among adolescents in Lithuania, Croatia, and the United States in the context of global obesity. *Croatian medical journal* **49**, 2008/05/08 ed., 233–42.

122. Zajc Petranovic M, Tomas Z, Smolej Narancic N, et al. (2014) A six decades long follow-up on body size in adolescents from Zagreb, Croatia (1951-2010). *Economics and human biology* **13**, 2013/11/10 ed., 155–64.

**Latin America and Caribbean**

1. Alvirde-García U, Rodríguez-Guerrero AJ, Henao-Morán S, et al. (2013) Results of a community-based life style intervention program for children. *Salud publica de mexico* **55 Suppl 3**, 406‐414.

2. Andrade S, Lachat C, Cardon G, et al. (2016) Two years of school-based intervention program could improve the physical fitness among Ecuadorian adolescents at health risk: subgroups analysis from a cluster-randomized trial. *BMC pediatrics* **16**, 51.

3. Aparco JP, Bautista-Olórtegui W & Pillaca J (2017) Impact evaluation of educational-motivational intervention ‘Como Jugando’ to prevent obesity in school children of Cercado de Lima: results in the first year. *Revista peruana de medicina experimental y salud publica* **34**. Rev Peru Med Exp Salud Publica.

4. Aparco JP, Bautista-Olórtegui W, Astete-Robilliard L, et al. (2016) Assessment of the nutritional status, physical activity, and eating habits of schoolchildren in Cercado de Lima. *Rev Peru Med Exp Salud Publica* **33**, 633–639.

5. Bacardí-Gascon M, Pérez-Morales ME & Jiménez-Cruz A (2012) A six month randomized school intervention and an 18-month follow-up intervention to prevent childhood obesity in Mexican elementary schools. *Nutricion hospitalaria* **27**, 755‐762.

6. Barbosa Filho VC, Bandeira ADS, Minatto G, et al. (2019) Effect of a multicomponent intervention on lifestyle factors among Brazilian adolescents from low human development index areas: a cluster-randomized controlled trial. *International journal of environmental research and public health* **16**.

7. Bui VQ, Marcinkevage J, Ramakrishnan U, et al. (2013) Associations among dietary zinc intakes and biomarkers of zinc status before and after a zinc supplementation program in Guatemalan schoolchildren. *Food and nutrition bulletin* **34**, 143‐150.

8. Caixeta HCV & Amato AA (2020) Factors associated with overweight and abdominal obesity in Brazilian school-aged children: a comprehensive approach. *Archives of endocrinology and metabolism*.

9. Campos V, Dan M & Eldridge A (2018) Nutritional state, food consumption and physical activity in a representative sample of young children from Sao Paulo, Brazil. *Journal of Pediatric Gastroenterology and Nutrition* **66**, 1128.

10. Cesar JA, Santos IS, Black RE, et al. (2020) Iodine status of Brazilian school-age children: a national cross-sectional survey. *Nutrients* **12**.

11. Chagas C, Melo G-S, Botelho RBA, et al. (2020) Effects of the Rango Cards game intervention on food consumption, nutritional knowledge and self-efficacy in the adoption of healthy eating practices of high school students: a cluster randomised controlled trial. *Public health nutrition*, 1‐10.

12. Cunha DB, de Souza Bda S, Pereira RA, et al. (2013) Effectiveness of a randomized school-based intervention involving families and teachers to prevent excessive weight gain among adolescents in Brazil. *PloS one* **8**, e57498.

13. da Silva Ferreira H, de Assunção Bezerra MK, Lopes de Assunção M, et al. (2016) Prevalence of and factors associated with anemia in school children from Maceió, northeastern Brazil. *BMC Public Health* **16**, 380.

14. da Silva KBB, Fiaccone RL, Couto RD, et al. (2015) Evaluation of the effects of a programme promoting adequate and healthy eating on adolescent health markers: an interventional study. *Nutricion hospitalaria* **32**. Nutr Hosp.

15. da Silva KBB, Ortelan N, Murta SG, et al. (2019) Evaluation of the computer-based intervention program Stayingfit Brazil to promote healthy eating habits: the results from a school cluster-randomized controlled trial. *International Journal of Environmental Research and Public Health* **16**. Multidisciplinary Digital Publishing Institute (MDPI).

16. da Silva LSM, Fisberg M, de Souza Pires MM, et al. (2013) The effectiveness of a physical activity and nutrition education program in the prevention of overweight in schoolchildren in Criciúma, Brazil. *Eur J Clin Nutr* **67**, 1200–1204.

17. de Fátima Guimarães R, Pereira da Silva M, Contiero San Martini M, et al. (2017) The effects of an after-school intervention program on physical activity level, sedentary time, and cardiovascular risk factors in adolescents. *Motriz: revista de educação física* **23**, e101769.

18. De la Cruz-Góngora V, Villalpando S & Shamah-Levy T (2018) Prevalence of anemia and consumption of iron-rich food groups in Mexican children and adolescents: Ensanut MC 2016. *Salud Publica Mex* **60**, 291–300.

19. Dekker LH, Mora-Plazas M, Marin C, et al. (2010) Stunting associated with poor socioeconomic and maternal nutrition status and respiratory morbidity in Colombian schoolchildren. *Food & Nutrition Bulletin* **31**, 242–50.

20. Di Gianfilippo M, Calvo MJ, Chavez M, et al. (2013) Overweight and obesity among elementary school students from Coquivacoa parish from Maracaibo - Venezuela. *Revista latinoamericana de hipertension* **8**, 68‐76.

21. Elizondo-Montemayor L, Moreno-Sànchez D, Gutierrez NG, et al. (2014) Individualized tailor-made dietetic intervention program at schools enhances eating behaviors and dietary habits in obese Hispanic children of low socioeconomic status. *TheScientificWorldJournal* **2014**, 484905.

22. Finkelstein JL, Mehta S, Villalpando S, et al. (2019) A randomized feeding trial of iron-biofortified beans on school children in Mexico. *Nutrients* **11**.

23. Fisberg M, Kovalskys I, Gomez G, et al. (2018) Total and added sugar intake: assessment in eight Latin American countries. *Nutrients* **10**, 22.

24. Francis M, Nichols SS & Dalrymple N (2010) The effects of a school-based intervention programme on dietary intakes and physical activity among primary-school children in Trinidad and Tobago. *Public health nutrition* **13**, 738‐747.

25. Galiano LP, Abril FM, Ernert A, et al. (2012) The double burden of malnutrition and its risk factors in school children in Tunja. *Arch Latinoam Nutr* **62**, 119–126.

26. Gandy J, Martinez H, Carmuega E, et al. (2018) Fluid intake of Latin American children and adolescents: results of four 2016 LIQ.IN 7 National Cross-Sectional Surveys. *European Journal of Nutrition* **57**, 53–63.

27. Gaskin PS, Hall RV, Chami P, et al. (2015) Associations of blood pressure with body composition among Afro-Caribbean children in Barbados. *PLoS ONE [Electronic Resource]* **10**, e0121107.

28. Gomez G, Fisberg RM, Nogueira Previdelli A, et al. (2019) Diet quality and diet diversity in eight Latin American countries: results from the Latin American Study of Nutrition and Health (ELANS). *Nutrients* **11**, 15.

29. Gómez Salas G, Ramírez Sanabria A, Sheik Oreamuno A, et al. (2020) Micronutrients inadequacy in urban population of Costa Rica. *ALAN* **69**, 221–232.

30. González-Rosendo G, Polo J, Rodríguez-Jerez JJ, et al. (2010) Bioavailability of a heme-iron concentrate product added to chocolate biscuit filling in adolescent girls living in a rural area of Mexico. *Journal of food science* **75**, H73‐8.

31. Guajardo VB, Favieri AL, Nogueira Previdelli A, et al. (2017) Reported dietary intake: analysis of calcium usual intake among argentine population. Results from ELANS study. *Annals of nutrition & metabolism* **71**, 1137‐.

32. Guevara D, Reyes S, Anarumba D, et al. (2016) Impact of milk based micronutrient supplementation on zinc, iron, and vitamin A deficiencies in school children in Quito-Ecuador. *FASEB journal. Conference: experimental biology 2016, EB. San diego, CA united states. Conference start: 20160402. Conference end: 20160406. Conference publication: (var.pagings)* **30**.

33. Iannotti L, Dulience S-L, Joseph S, et al. (2016) Fortified snack reduced anemia in rural school-aged children of Haiti: a cluster-randomized, controlled trial. *Plos one* **11**.

34. Iannotti LL, Henretty NM, Delnatus JR, et al. (2015) Ready-to-use supplementary food increases fat mass and BMI in Haitian school-aged children. *Journal of nutrition* **145**, 813‐822.

35. Iannotti LL, Delnatus JR, Odom AR, et al. (2015) Determinants of anemia and hemoglobin concentration in Haitian school-aged children. *Am J Trop Med Hyg* **93**, 1092–1098.

36. Jimenez-Mora MA, Nieves-Barreto LD, Montano-Rodriguez A, et al. (2020) Association of overweight, obesity and abdominal obesity with socioeconomic status and educational level in Colombia. *Diabetes, Metabolic Syndrome and Obesity: Targets and Therapy* **13**, 1887–1898.

37. Kain J, Concha F, Moreno L, et al. (2014) School-based obesity prevention intervention in Chilean children: effective in controlling, but not reducing obesity. *Journal of Obesity* **2014**, e618293. Hindawi.

38. Kong AS, Sussman AL, Yahne C, et al. (2013) School-based health center intervention improves body mass index in overweight and obese adolescents. *Journal of obesity* **2013**, 575016.

39. Kovalskys I, Fisberg M, Gomez G, et al. (2018) Energy intake and food sources of eight Latin American countries: results from the Latin American Study of Nutrition and Health (ELANS). *Public health nutrition* **21**, 2535–2547.

40. Lázaro Cuesta L, Rearte A, Rodríguez S, et al. (2018) Anthropometric and biochemical assessment of nutritional status and dietary intake in school children aged 6-14 years, Province of Buenos Aires, Argentina. *Arch Argent Pediatr* **116**, e34–e46.

41. Leme AC, Lubans DR, Guerra PH, et al. (2016) Preventing obesity among Brazilian adolescent girls: six-month outcomes of the Healthy Habits, Healthy Girls-Brazil school-based randomized controlled trial. *Preventive medicine* **86**, 77‐83.

42. Maitland T, Malcolm S & Handfield S (2015) Nutritional knowledge and practices, lifestyle characteristics and anthropometric status of Turks and Caicos Islands elementary school children. *West Indian Med J* **64**, 29–36.

43. Masuet-Aumatell C, Ramon-Torrell JM, Banqué-Navarro M, et al. (2015) Nutritional status of children from Cochabamba, Bolivia: a cross-sectional study. *Rev Panam Salud Publica* **38**, 487–494.

44. Mendes M, de Melo ME, Fernandes AE, et al. (2016) Effects of two diet techniques and delivery mode on weight loss, metabolic profile and food intake of obese adolescents: a fixed diet plan and a calorie-counting diet. *European journal of clinical nutrition* **(no pagination)**.

45. Miranda M, Olivares M, Brito A, et al. (2014) Reducing iron deficiency anemia in Bolivian school children: calcium and iron combined versus iron supplementation alone. *Nutrition (burbank, los angeles county, calif.)* **30**, 771‐775.

46. Morales Pernalete AR, Gordillo Gutierrez CA, Pérez Alvarado CJ, et al. (2014) Risk factors for binge eating disorders and its association with obesity in adolescents. *Gac Med Mex* **150 Suppl 1**, 125–131.

47. Morshed AB, Becker HV, Delnatus JR, et al. (2016) Early nutrition transition in Haiti: linking food purchasing and availability to overweight status in school-aged children. *Public Health Nutr* **19**, 3378–3385.

48. Naqvi A, Solomons NW, Campos R, et al. (2017) Vitamin D status among indigenous Mayan (Kekchi) and Afro-Caribe (Garifuna) adolescents from Guatemala: a comparative description between two ethnic groups residing on the Rio Dulce at the Caribbean coast in Izabal Province, Guatemala. *Public Health Nutrition* **20**, 1729–1737.

49. Ochoa-Avilés A, Verstraeten R, Huybregts L, et al. (2017) A school-based intervention improved dietary intake outcomes and reduced waist circumference in adolescents: a cluster randomized controlled trial. *Nutrition journal* **16**, 79.

50. Ortega P, Leal J, Amaya D, et al. (2010) Nutritional evaluation, micronutrient deficiencies and anemia among female adolescents in an urban and a rural zone from Zulia state, Venezuela. *Investigacion Clinica* **51**, 37–52.

51. Ortiz A & Pereyra I (2015) Study of food characteristics of Uruguayan adolescents. *Archivos Latinoamericanos de Nutricion* **65**, 97–103.

52. Passos MAZ, Cintra I de P, Branco LM, et al. (2010) Body mass index percentiles in adolescents of the city of São Paulo, Brazil, and their comparison with international parameters. *Arq Bras Endocrinol Metabol* **54**, 295–302.

53. Pehlke EL, Letona P, Hurley K, et al. (2016) Guatemalan school food environment: impact on schoolchildren’s risk of both undernutrition and overweight/obesity. *Health Promotion International* **31**, 542–50.

54. Pereira FN, Oliveira JR de, Zöllner CC, et al. (2013) Body weight perception and associated factors in students. *J. Hum. Growth Dev.* **23**, 296–302.

55. Quizán-Plata T, Villarreal Meneses L, Esparza Romero J, et al. (2014) Educational program had a positive effect on the intake of fat, fruits and vegetables and physical activity in students attending public elementary schools of Mexico. *Nutricion hospitalaria* **30**, 552‐561.

56. Rauber F, Hoffman DJ & Vitolo MR (2014) Diet quality from pre-school to school age in Brazilian children: a 4-year follow-up in a randomised control study. *British journal of nutrition* **111**, 499‐505.

57. Rebolledo N, Reyes M, Corvalan C, et al. (2019) Dietary intake by food source and eating location in low- and middle-income chilean preschool children and adolescents from southeast Santiago. *Nutrients* **11**.

58. Safdie M, Jennings-Aburto N, Lévesque L, et al. (2013) Impact of a school-based intervention program on obesity risk factors in Mexican children. *Salud publica de Mexico* **55 Suppl 3**, 374‐387.

59. Safdie M, Lévesque L, González-Casanova I, et al. (2013) Promoting healthful diet and physical activity in the Mexican school system for the prevention of obesity in children. *Salud publica de Mexico* **55 Suppl 3**, 357‐373.

60. Sarmiento OL, Parra DC, González SA, et al. (2014) The dual burden of malnutrition in Colombia. *Am J Clin Nutr* **100**, 1628S–35S.

61. Schwiebbe L, van Rest J, Verhagen E, et al. (2011) Childhood obesity in the Caribbean. *West Indian Med J* **60**, 442–445.

62. Shamah Levy T, Morales Ruán C, Amaya Castellanos C, et al. (2012) Effectiveness of a diet and physical activity promotion strategy on the prevention of obesity in Mexican school children. *BMC public health* **12**, 152.

63. Taillie LS, Afeiche MC, Eldridge AL, et al. (2015) Increased snacking and eating occasions are associated with higher energy intake among Mexican children aged 2-13 years. *Journal of Nutrition* **145**, 2570–2577.

64. Toral N & Slater B (2012) Intervention based exclusively on stage-matched printed educational materials regarding healthy eating does not result in changes to adolescents’ dietary behavior. *Thescientificworldjournal* **2012**, 174640.

65. Valero P, Prieto C, García D, et al. Consumption of snacks and its relationship with lipid profile in school children and adolescents of the municipality of Maracaibo, estado Zulia. *Revista Latinoamericana de Hipertensión* **13**, 194–201.

66. Vilchis-Gil J, Klünder-Klünder M & Flores-Huerta S (2018) Effect on the metabolic biomarkers in schoolchildren after a comprehensive intervention using electronic media and in-person sessions to change lifestyles: community trial. *Journal of Medical Internet Research* **20**. JMIR Publications Inc.

67. Villalobos D, Bravo A, Duque M, et al. (2013) Nutritional status and dietary patterns in schoolchildren of the wayuu ethnic group from Maracaibo, Venezuela. *Annals of Nutrition and Metabolism* **1)**, 1078.

**Middle East and North Africa**

1. Abd El-Shaheed A, Mahfouz NN, Moustafa RSI, et al. (2019) Alarming eating behaviours among adolescents in Egypt. *Open Access Maced J Med Sci* **7**, 2189–2193.

2. Abd-El Wahed MA, Mohamed MH, Ibrahim SS, et al. (2014) Iron profile and dietary pattern of primary school obese Egyptian children. *J Egypt Public Health Assoc* **89**, 53–59.

3. Abdel Wahed WY, Hassan SK & Eldessouki R (2017) Malnutrition and its associated factors among rural school children in Fayoum Governorate, Egypt. *Journal of Environmental and Public Health* **2017**. Hindawi Limited.

4. Abduelkarem AR, Sharif SI, Bankessli FG, et al. (2020) Obesity and its associated risk factors among school-aged children in Sharjah, UAE. *PLoS ONE* **15**. Public Library of Science.

5. Abiri B, Sarbakhsh P & Vafa M (2019) Prevalence of overweight, obesity, and associated risk factors in healthy female adolescents in Tehran, Iran. *Central Asian Journal of Global Health* **8**. University Library System, University of Pittsburgh.

6. Abudayya AH, Stigum H, Shi Z, et al. (2009) Sociodemographic correlates of food habits among school adolescents (12-15 year) in north Gaza Strip. *BMC Public Health* **9**.

7. AbuKishk N, Turki Y, Saleh S, et al. (2020) Anaemia prevalence in children newly registered at UNRWA schools: a cross-sectional study. *BMJ open* **10**, e034705–e034705. NLM (Medline).

8. Ahadi Z, Qorbani M, Kelishadi R, et al. (2015) Association between breakfast intake with anthropometric measurements, blood pressure and food consumption behaviors among Iranian children and adolescents: the CASPIAN-IV study. *Public Health* **129**, 740–747. Elsevier.

9. Akbari-Sedigh A, Asghari G, Yuzbashian E, et al. (2019) Association of dietary pattern with carotid intima media thickness among children with overweight or obesity. *Diabetology and Metabolic Syndrome* **11**. BioMed Central Ltd.

10. Al Dahi AS, Al Balawi FA, Al Balawi SS, et al. (2016) Prevalence of overweight and obesity among Saudi primary school students in Tabuk, Saudi Arabia 2015. *Bangladesh Journal of Medical Science* **15**, 329–334. Ibn Sina Trust.

11. Al Sabbah H, Vereecken C, Abdeen Z, et al. (2009) Associations of overweight and of weight dissatisfaction among Palestinian adolescents: findings from the national study of Palestinian schoolchildren (HBSC-WBG2004). *Journal of Human Nutrition and Dietetics* **22**, 40–49.

12. Al Sabbah H, Vereecken C, Kolsteren P, et al. (2007) Food habits and physical activity patterns among Palestinian adolescents: findings from the national study of Palestinian schoolchildren (HBSC-WBG2004). *Public Health Nutrition* **10**, 739–746.

13. Al-Daghri NM, Al-Saleh Y, Aljohani N, et al. (2015) Vitamin D deficiency and cardiometabolic risks: a juxtaposition of arab adolescents and adults. *PLoS ONE* **10**. Public Library of Science.

14. Al-Domi HA, Faqih A, Jaradat Z, et al. (2019) Physical activity, sedentary behaviors and dietary patterns as risk factors of obesity among jordanian schoolchildren. *Diabetes and Metabolic Syndrome: Clinical Research and Reviews* **13**, 189–194. Elsevier Ltd.

15. Al-Ghannami SS, Sedlak E, Hussein IS, et al. (2016) Lipid-soluble nutrient status of healthy Omani school children before and after intervention with oily fish meal or re-esterified triacylglycerol fish oil. *Nutrition* **32**, 73–78. Elsevier Inc.

16. Al-Haifi AR, Al-Fayez MA, Al-Athari BI, et al. (2013) Relative contribution of physical activity, sedentary behaviors, and dietary habits to the prevalence of obesity among Kuwaiti adolescents. *Food and Nutrition Bulletin* **34**, 6–13. United Nations University Press.

17. Al-Hazzaa HM, Abahussain NA, Al-Sobayel HI, et al. (2011) Physical activity, sedentary behaviors and dietary habits among Saudi adolescents relative to age, gender and region. *International Journal of Behavioral Nutrition and Physical Activity* **8**.

18. Al-Hazzaa HM, Al-Nakeeb Y, Duncan MJ, et al. (2013) A cross-cultural comparison of health behaviors between Saudi and British adolescents living in urban areas: gender by country analyses. *International Journal of Environmental Research and Public Health* **10**, 6701–6720.

19. Al-Kloub MI, Al-Khawaldeh OA, ALBashtawy M, et al. (2019) Disordered eating in Jordanian adolescents. *International Journal of Nursing Practice* **25**. Wiley-Blackwell.

20. Al-Kutbe R, Payne A, De Looy A, et al. (2017) A comparison of nutritional intake and daily physical activity of girls aged 8-11 years old in Makkah, Saudi Arabia according to weight status. *BMC Public Health* **17**. BioMed Central Ltd.

21. Al-Mohaimeed A, Ahmed S, Dandash K, et al. (2015) Concordance of obesity classification between body mass index and percent body fat among school children in Saudi Arabia. *BMC Pediatrics* **15**. BioMed Central Ltd.

22. Al-Musharaf S, Al-Othman A, Al-Daghri NM, et al. (2012) Vitamin D deficiency and calcium intake in reference to increased body mass index in children and adolescents. *European Journal of Pediatrics* **171**, 1081–1086.

23. Al-Raddadi R, Bahijri S, Borai A, et al. (2018) Prevalence of lifestyle practices that might affect bone health in relation to vitamin D status among female Saudi adolescents. *Nutrition* **45**, 108–113. Elsevier Inc.

24. Al-Saleh Y, Al-Daghri NM, Khan N, et al. (2015) Vitamin D status in Saudi school children based on knowledge. *BMC Pediatrics* **15**, 1–1. BioMed Central Ltd.

25. Al-Taiar A, Rahman A, Al-Sabah R, et al. (2018) Vitamin D status among adolescents in Kuwait: a cross-sectional study. *BMJ Open* **8**. BMJ Publishing Group.

26. Al-Yateem N & Rossiter R (2017) Nutritional knowledge and habits of adolescents aged 9 to 13 years in Sharjah, United Arab emirates: a cross-sectional study. *Eastern Mediterranean Health Journal* **23**, 551–558. World Health Organization.

27. Alazzeh A, AlShammari E, Smadi M, et al. (2018) Some socioeconomic factors and lifestyle habits influencing the prevalence of obesity among adolescent male students in the Hail region of Saudi Arabia. *Children* **5**, 39–39. MDPI AG.

28. AlFaris NA, Al-Tamimi JZ, Al-Jobair MO, et al. (2015) Trends of fast food consumption among adolescent and young adult Saudi girls living in Riyadh. *Food and Nutrition Research* **59**. Co-Action Publishing.

29. Ali HI, Ng SW, Zaghloul S, et al. (2013) High proportion of 6 to 18-year-old children and adolescents in the United Arab Emirates are not meeting dietary recommendations. *Nutrition Research* **33**, 447–456.

30. Alizadeh M, Didarloo A & Esmaillzadeh A (2015) Dietary patterns of young females and their association with waist circumference as a health index in northwest of Iran, 2007. *Iranian Red Crescent Medical Journal* **17**. Kowsar Medical Publishing Company.

31. Alkazemi DUZ & Saleh A (2019) Adequacy of dairy product intake among children in Kuwait using a short dietary assessment questionnaire. *Nutrition and Food Science* **49**, 112–128. Emerald Group Publishing Ltd.

32. Allafi A, Al-Haifi AR, Al-Fayez MA, et al. (2014) Physical activity, sedentary behaviours and dietary habits among Kuwaiti adolescents: gender differences. *Public Health Nutrition* **17**, 2045–2052. Cambridge University Press.

33. Almuhlafi M, Jamilah KA, Almutairi AF, et al. (2018) Relationship between early menarche, obesity, and disordered eating behaviors: A school-based cross-sectional survey in Northern Saudi Arabia. *Diabetes, Metabolic Syndrome and Obesity: Targets and Therapy* **11**, 743–751. Dove Medical Press Ltd.

34. Alquaiz AJM, Khoja TAM, Alsharif A, et al. (2015) Prevalence and correlates of anaemia in adolescents in Riyadh city, Kingdom of Saudi Arabia. *Public Health Nutrition* **18**, 3192–3200. Cambridge University Press.

35. Alshammari E, Suneetha E, Adnan M, et al. (2017) Growth profile and its association with nutrient intake and dietary patterns among children and adolescents in Hail Region of Saudi Arabia. *BioMed Research International* **2017**. Hindawi Limited.

36. Alsubaie ASR (2018) Intake of fruit, vegetables and milk products and correlates among school boys in Saudi Arabia. *International Journal of Adolescent Medicine and Health*. Walter de Gruyter GmbH.

37. Alyahya KO (2017) Vitamin D levels in schoolchildren: a cross-sectional study in Kuwait. *BMC Pediatrics* **17**. BioMed Central Ltd.

38. Anzid K, Baali A, Vimard P, et al. (2014) Inadequacy of vitamins and minerals among high-school pupils in Ouarzazate, Morocco. *Public Health Nutrition* **17**, 1786–1795. Cambridge University Press.

39. Aounallah-Skhiri H, Romdhane HB, Traissac P, et al. (2008) Nutritional status of Tunisian adolescents: associated gender, environmental and socio-economic factors. *Public Health Nutrition* **11**, 1306–1317.

40. Aounallah-Skhiri H, Traissac P, El Ati J, et al. (2011) Nutrition transition among adolescents of a south-Mediterranean country: dietary patterns, association with socio-economic factors, overweight and blood pressure. A cross-sectional study in Tunisia. *Nutrition Journal* **10**.

41. Assaad S, Anouti S, Naja F, et al. (2018) Adolescents’ self-perceived and actual weight: which plays a dominant role in weight loss behaviour in Lebanon? *Child: Care, Health and Development* **44**, 124–130. Blackwell Publishing Ltd.

42. Azadbakht L, Akbari F, Qorbani M, et al. (2019) Dinner consumption and cardiovascular disease risk factors among a nationally representative sample of Iranian adolescents: the CASPIAN-III Study. *Journal of Cardiovascular and Thoracic Research* **11**, 138–146. Maad Rayan Publishing Company.

43. Azadbakht L, Haghighatdoost F & Esmaillzadeh A (2016) White rice consumption, body mass index, and waist circumference among Iranian female adolescents. *Journal of the American College of Nutrition* **35**, 491–499. Routledge.

44. Azadbakht L, Hajishafiee M, Golshahi J, et al. (2016) Snacking behavior and obesity among female adolescents in Isfahan, Iran. *Journal of the American College of Nutrition* **35**, 405–412. Routledge.

45. Azekour K, Idir I, Lahrach N, et al. (2020) Prevalence of obesity and overweight within the school environment in tafilalet oasis, south-east of Morocco. *Pan African Medical Journal* **35**. African Field Epidemiology Network.

46. Bader Z, Musaiger AO, Al-Roomi K, et al. (2008) Overweight and obesity among adolescent in Bahrain. *Anthropologischer Anzeiger* **66**, 401–408. E. Schweizerbart’sche Verlagsbuchhandlung.

47. Bagherniya M, Darani FM, Sharma M, et al. (2019) Qualitative study to determine stressors influencing dietary and physical activity behaviors of overweight and obese adolescents in Iran. *International Journal of Preventive Medicine* **10**. Wolters Kluwer Medknow Publications.

48. Bagherniya M, Sharma M, Mostafavi Darani F, et al. (2017) School-based nutrition education intervention using social cognitive theory for overweight and obese Iranian adolescent girls: a cluster randomized controlled trial. *International Quarterly of Community Health Education* **38**, 37–45. SAGE Publications Inc.

49. Bastami F, Zamani-Alavijeh F & Mostafavi F (2019) Factors behind healthy snack consumption at school among high-school students: a qualitative study. *BMC Public Health* **19**. BioMed Central Ltd.

50. Baygi F, Heshmat R, Kelishadi R, et al. (2015) Regional disparities in sedentary behaviors and meal frequency in Iranian adolescents: the CASPIAN-III study. *Iranian Journal of Pediatrics* **25**. Tehran University of Medical Sciences (TUMS).

51. Bener A (2006) Prevalence of obesity, overweight, and underweight in Qatari adolescents. *Food and Nutrition Bulletin* **27**, 39–45. United Nations University Press.

52. Bener A, Al-Ali M & Hoffmann GF (2009) Vitamin D deficiency in healthy children in a sunny country: associated factors. *International Journal of Food Sciences and Nutrition* **60**, 60–70.

53. Bezrati I, Fradj MKB, Ouerghi N, et al. (2016) Vitamin D inadequacy is widespread in Tunisian active boys and is related to diet but not to adiposity or insulin resistance. *Libyan Journal of Medicine* **11**. Co-Action Publishing.

54. Chakar H & Salameh PR (2006) Adolescent obesity in Lebanese private schools. *European Journal of Public Health* **16**, 648–651.

55. Cherkaoui Dekkaki I, Mouane N, Ettair S, et al. (2011) Prevalence of obesity and overweight in children: a study in government primary schools in rabat, Morocco. *Archives of Medical Research* **42**, 703–708.

56. Collison KS, Zaidi MZ, Subhani SN, et al. (2010) Sugar-sweetened carbonated beverage consumption correlates with BMI, waist circumference, and poor dietary choices in school children. *BMC Public Health* **10**.

57. Dahifar H, Faraji A, Ghorbani A, et al. (2006) Impact of dietary and lifestyle on vitamin D in healthy student girls aged 11-15 years. *Journal of Medical Investigation* **53**, 204–208.

58. Eftekhari MH, Mozaffari-Khosravi H & Shidfar F (2009) The relationship between BMI and iron status in iron-deficient adolescent Iranian girls. *Public Health Nutrition* **12**, 2377–2381.

59. El Harake MD, Kharroubi S, Hamadeh SK, et al. (2018) Impact of a pilot school-based nutrition intervention on dietary knowledge, attitudes, behavior and nutritional status of syrian refugee children in the bekaa, lebanon. *Nutrients* **10**. MDPI AG.

60. El Kabbaoui M, Chda A, Bousfiha A, et al. (2018) Prevalence of and risk factors for overweight and obesity among adolescents in Morocco. *Eastern Mediterranean Health Journal* **24**, 512–521. World Health Organization.

61. El Khoury R, Sleilaty G & Gannagé-Yared MH (2020) Prevalence of Iron deficiency in Lebanese schoolchildren. *European Journal of Clinical Nutrition* **74**, 1157–1163. Springer Nature.

62. El-Kassas G & Ziade F (2017) Exploration of the risk factors of generalized and central obesity among adolescents in north Lebanon. *Journal of Environmental and Public Health* **2017**. Hindawi Limited.

63. El-Kassas G & Ziade F (2017) The dual burden of malnutrition and associated dietary and lifestyle habits among Lebanese school age children living in orphanages in north Lebanon. *Journal of Nutrition and Metabolism* **2017**. Hindawi Limited.

64. El-Shafie AM, Kasemy ZA, Omar ZA, et al. (2020) Prevalence of short stature and malnutrition among Egyptian primary school children and their coexistence with Anemia. *Italian Journal of Pediatrics* **46**. BioMed Central.

65. Elseifi OS, Abdelrahman DM & Mortada EM (2020) Effect of a nutritional education intervention on breakfast consumption among preparatory school students in Egypt. *International Journal of Public Health* **65**, 893–903. Springer.

66. Emamian MH, Hashemi H & Fotouhi A (2019) Obesity and underweight: serious health problems in Iranian primary school children. *Pediatrics International* **61**, 1030–1035. Blackwell Publishing.

67. Esmaili H, Bahreynian M, Qorbani M, et al. (2015) Prevalence of general and abdominal obesity in a nationally representative sample of Iranian children and adolescents: the CASPIAN-IV study. *Iranian Journal of Pediatrics* **25**. Tehran University of Medical Sciences (TUMS).

68. Ghadimi R, Asgharzadeh E & Sajjadi P (2015) Obesity among elementary schoolchildren: a growing concern in the north of Iran, 2012. *International Journal of Preventive Medicine* **2015**. Isfahan University of Medical Sciences(IUMS).

69. Gharib N & Rasheed P (2011) Energy and macronutrient intake and dietary pattern among school children in Bahrain: a cross-sectional study. *Nutrition Journal* **10**.

70. Ghasemi A, Zahediasl S, Hosseini-Esfahani F, et al. (2012) Pediatric reference values for serum zinc concentration in Iranian subjects and an assessment of their dietary zinc intakes. *Clinical Biochemistry* **45**, 1254–1256.

71. Ghazi HF, Md. Isa Z, Shah SA, et al. (2014) The relationship between the neighborhood safety and nutritional status of children in Baghdad City, Iraq. *Journal of Nutrition and Metabolism* **2014**. Hindawi Publishing Corporation.

72. Ghobadi S, Totosy De Zepetnek JO, Hemmatdar Z, et al. (2018) Association between overweight/obesity and eating habits while watching television among primary-school children in the city of Shiraz, Iran. *Public Health Nutrition* **21**, 571–579. Cambridge University Press.

73. Haghani S, Shahnazi H & Hassanzadeh A (2017) Effects of tailored health education program on overweight elementary school students’ obesity-related lifestyle: a school-based interventional study. *Oman Medical Journal* **32**, 140–147. Oman Medical Specialty Board.

74. Hatami M, Taib MNM, Jamaluddin R, et al. (2014) Dietary factors as the major determinants of overweight and obesity among Iranian adolescents. A cross-sectional study. *Appetite* **82**, 194–201. Academic Press.

75. Hojhabrimanesh A, Akhlaghi M, Rahmani E, et al. (2017) A Western dietary pattern is associated with higher blood pressure in Iranian adolescents. *European Journal of Nutrition* **56**, 399–408. Dr. Dietrich Steinkopff Verlag GmbH and Co. KG.

76. Jafari S, Fouladgar M, Naeeni MM, et al. (2014) Body mass index, weight-for-age, and stature-for-age indices in Iranian school children in relation to weight and growth disorders: a population-based survey. *International Journal of Preventive Medicine* **5**, S133–S138. Isfahan University of Medical Sciences(IUMS).

77. Jamalikandazi F, Ranjbar E, Gholami-Parizad E, et al. (2016) Nutritional status and anthropometric indices in high school girls in Ilam, west Iran. *Scientifica* **2016**. Hindawi Limited.

78. Jildeh C, Papandreou C, Mourad TA, et al. (2011) Assessing the nutritional status of Palestinian adolescents from east Jerusalem: a school-based study 2002-03. *Journal of Tropical Pediatrics* **57**, 51–58.

79. Kelishadi R, Ardalan G, Gheiratmand R, et al. (2008) Thinness, overweight and obesity in a national sample of Iranian children and adolescents: CASPIAN Study. *Child: Care, Health and Development* **34**, 44–54.

80. Maddah M, Mohtasham-Amiri Z, Rashidi A, et al. (2007) Height and weight of urban preschool children in relation to their mothers’ educational levels and employment status in Rasht City, northern Iran. *Matern Child Nutr* **3**, 52–57.

81. Maddah M, Rashidi A, Mohammadpour B, et al. (2009) In-school snacking, breakfast consumption, and sleeping patterns of normal and overweight Iranian high school girls: a study in urban and rural areas in Guilan, Iran. *Journal of Nutrition Education and Behavior* **41**, 27–31.

82. Mahfouz A, Shatoor A, Khan M, et al. (2011) Nutrition, physical activity, and gender risks for adolescent obesity in southwestern Saudi Arabia. *Saudi Journal of Gastroenterology* **17**, 318–322.

83. Malik M & Bakir A (2007) Prevalence of overweight and obesity among children in the United Arab Emirates. *Obesity Reviews* **8**, 15–20.

84. Massad S, Deckelbaum RJ, Gebre-Medhin M, et al. (2016) Double burden of undernutrition and obesity in Palestinian schoolchildren. *Food and Nutrition Bulletin* **37**, 144–152. SAGE Publications Inc.

85. Mikki N, Abdul-Rahim HF, Awartani F, et al. (2009) Prevalence and sociodemographic correlates of stunting, underweight, and overweight among Palestinian school adolescents (13-15 years) in two major governorates in the West Bank. *BMC Public Health* **9**.

86. Mirmiran P, Azadbakht L & Azizi F (2007) Dietary behaviour of Tehranian adolescents does not accord with their nutritional knowledge. *Public Health Nutrition* **10**, 897–901.

87. Moayeri H, Bidad K, Aghamohammadi A, et al. (2006) Overweight and obesity and their associated factors in adolescents in Tehran, Iran, 2004-2005. *European Journal of Pediatrics* **165**, 489–493.

88. Motlagh ME, Shirvani S, Hassanzadeh-Rostami Z, et al. (2018) Assessment of overweight and obesity in Iranian adolescents: optimal cut-off values of anthropometric indices. *Eastern Mediterranean Health Journal* **24**, 975–987. World Health Organization.

89. Moukhyer ME, Mukhayer A, Elfaki FA, et al. (2019) Body mass index, haemoglobin status and eating behaviours among adolescents in Jazan, Saudi Arabia: a cross-sectional study. *Mediterranean Journal of Nutrition and Metabolism* **12**, 283–292. IOS Press.

90. Mozaffari-Khosravi H, Karandish M, Hadianfard AM, et al. (2020) The relationship between sleep quality and breakfast, mid-morning snack, and dinner and physical activity habits among adolescents: a cross-sectional study in Yazd, Iran. *Sleep and Biological Rhythms*. Springer Japan.

91. Musaiger AO, Al-Mannai M & Al-Marzog Q (2014) Overweight and obesity among children (10-13 years) in bahrain: a comparison between two international standards. *Pakistan Journal of Medical Sciences* **30**. Professional Medical Publications.

92. Musaiger AO, Al-Mannai M, Tayyem R, et al. (2012) Prevalence of overweight and obesity among adolescents in seven Arab Countries: a cross-cultural study. *Journal of Obesity* **2012**.

93. Musaiger AO, Al-Mannai M & Tayyem R (2012) Prevalence of overweight and obesity among female adolescents in Jordan: a comparison between two international reference standards. *Pakistan Journal of Medical Sciences* **29**. Professional Medical Publications.

94. Musaiger AO, Al-Mufty BA & Al-Hazzaa HM (2014) Eating habits, inactivity, and sedentary behavior among adolescents in Iraq: sex differences in the hidden risks of noncommunicable diseases. *Food and Nutrition Bulletin* **35**, 12–19. United Nations University Press.

95. Musaiger AO, Al-Roomi K & Bader Z (2014) Social, dietary and lifestyle factors associated with obesity among Bahraini adolescents. *Appetite* **73**, 197–204.

96. Musaiger AO, Bader Z, Al-Roomi K, et al. (2011) Dietary and lifestyle habits amongst adolescents in Bahrain. *Food and Nutrition Research* **55**. Swedish Nutrition Foundation.

97. Nabhani-Zeidan M, Naja F & Nasreddine L (2011) Dietary intake and nutrition-related knowledge in a sample of Lebanese adolescents of contrasting socioeconomic status. *Food and Nutrition Bulletin* **32**, 75–83. United Nations University Press.

98. Naja F, Hwalla N, Itani L, et al. (2015) A Western dietary pattern is associated with overweight and obesity in a national sample of Lebanese adolescents (13-19 years): a cross-sectional study. *British Journal of Nutrition* **114**, 1909–1919. Cambridge University Press.

99. Nasiri-babadi P, Sadeghian M, Sadeghi O, et al. (2020) The association of serum levels of zinc and vitamin D with wasting among Iranian pre-school children. *Eating and Weight Disorders*. Springer.

100. Nasreddine L, Mehio-Sibai A, Mrayati M, et al. (2010) Adolescent obesity in Syria: prevalence and associated factors. *Child: Care, Health and Development* **36**, 404–413.

101. Nasreddine L, Naja F, Akl C, et al. (2014) Dietary, lifestyle and socio-economic correlates of overweight, obesity and central adiposity in Lebanese children and adolescents. *Nutrients* **6**, 1038–1062. MDPI AG.

102. Neyestani TR, Nikooyeh B, Hajifaraji M, et al. (2016) The prevalence of zinc deficiency and its correlation with iron status and economical living area in 9-12-year-old children. *International Journal for Vitamin and Nutrition Research* **86**, 18–26. Verlag Hans Huber AG.

103. Nouayti H, Bouanani NH, Hammoudi J, et al. (2020) Overweight and obesity in Eastern Morocco: prevalence and associated risk factors among high school students. *Revue d’Epidemiologie et de Sante Publique* **68**, 295–301. Elsevier Masson s.r.l.

104. Oulamara H, Agli AN & Frelut ML (2009) Changes in the prevalence of overweight, obesity and thinness in Algerian children between 2001 and 2006. *International Journal of Pediatric Obesity* **4**, 411–413.

105. Oulamara H, Allam O, Tebbani F, et al. (2020) Prevalence of overweight and underweight in schoolchildren in Constantine, Algeria: comparison of four reference cut-off points for body mass index. *Eastern Mediterranean Health Journal* **26**, 349–355. World Health Organization.

106. Rahmanian M, Kelishadi R, Qorbani M, et al. (2014) Dual burden of body weight among Iranian children and adolescents in 2003 and 2010: the CASPIAN-III study. *Archives of Medical Science* **10**, 96–103.

107. Said L, Gubbels JS & Kremers SPJ (2020) Dietary knowledge, dietary adherence, and bmi of lebanese adolescents and their parents. *Nutrients* **12**, 1–14. MDPI AG.

108. Salamoun MM, Kizirian AS, Tannous RI, et al. (2005) Low calcium and vitamin D intake in healthy children and adolescents and their correlates. *European Journal of Clinical Nutrition* **59**, 177–184.

109. Salazar-Martinez E, Allen B, Fernandez-Ortega C, et al. (2006) Overweight and obesity status among adolescents from Mexico and Egypt. *Archives of Medical Research* **37**, 535–542.

110. Sedaghat F, Naja F, Darand M, et al. (2019) Adherence to a Mediterranean dietary pattern and overweight and obesity among female adolescents in Iran. *International Journal of Adolescent Medicine and Health* **31**. De Gruyter.

111. Shokrvash B, Salehi L, Hariri Akbari M, et al. (2015) Social support and dairy products intake among adolescents: a study from Iran. *BMC Public Health* **15**, 1078.

112. Siddiqui AM & Kamfar HZ (2007) Prevalence of vitamin D deficiency rickets in adolescent school girls in Western region, Saudi Arabia. *Saudi Medical Journal* **28**, 441–444.

113. Soheilipour F, Salehiniya H, Farajpour.kh M, et al. (2019) Breakfast habits, nutritional status and their relationship with academic performance in elementary school students of Tehran, Iran. *Medicine and Pharmacy Reports* **92**, 52–58.

114. Subih HS, Abu-Shquier Y, Bawadi H, et al. (2018) Assessment of body weight, maternal dietary knowledge and lifestyle practices among children and adolescents in north Jordan. *Public Health Nutrition* **21**, 2803–2810. Cambridge University Press.

115. Sulimani RA, Mohammed AG, Alfadda AA, et al. (2016) Vitamin D deficiency and biochemical variations among urban Saudi adolescent girls according to season. *Saudi Medical Journal* **37**, 1002–1008. Saudi Arabian Armed Forces Hospital.

116. Talaie-Zanjani A, Faraji F, Rafie M, et al. (2014) A comparative study of nutritional status and foodstuffs in adolescent girls in iran. *Ann Med Health Sci Res* **4**, 38–43.

117. Tayyem RF, Bawadi HA, AbuMweis SS, et al. (2016) Association between mass media and body weight concern among Jordanian adolescents’ residents of Amman: the role of gender and obesity. *Environmental Health and Preventive Medicine* **21**, 430–438. Springer Tokyo.

118. Vakili M, Abedi P, Sharifi M, et al. (2013) Dietary diversity and its related factors among adolescents: a survey in Ahvaz-Iran. *Global journal of health science* **5**, 181–186.

119. Washi SA & Ageib MB (2010) Poor diet quality and food habits are related to impaired nutritional status in 13- to 18-year-old adolescents in Jeddah. *Nutrition Research* **30**, 527–534.

120. Zarrati M, Hojaji E, Razmpoosh E, et al. (2016) Is high waist circumference and body weight associated with high blood pressure in Iranian primary school children? *Eating and Weight Disorders* **21**, 687–693. Springer International Publishing.

**South Asia**

1. Acharya B, Chauhan HS, Bala I, et al. (2016) Body image satisfaction, weight perception and knowledge of obesity among adolescents in Kaski district, Nepal. *TAF Preventive Medicine Bulletin* **15**, 396–400.

2. Adams AM, Ahmed R, Latif AH, et al. (2017) Impact of fortified biscuits on micronutrient deficiencies among primary school children in Bangladesh. *PLoS ONE* **12**, e0174673.

3. Adhikari RP, Yogi S, Acharya A, et al. (2020) Intimate partner violence and nutritional status among nepalese women: an investigation of associations. *BMC Women’s Health* **20**.

4. Afridi IUK, Afridi H, Riaz B, et al. (2017) Frequency of iron deficiency anemia in children: cross-sectional survey of outpatients at Akhtar Saeed Trust Teaching Hospital, Lahore from 2016-17. *Pakistan Journal of Medical and Health Sciences* **11**, 1365–1368.

5. Ahankari AS, Dixit JV, Fogarty AW, et al. (2016) Comparison of the NBM 200 non-invasive haemoglobin sensor with Sahli’s haemometer among adolescent girls in rural India. *BMJ Innovations* **2**, 144–148.

6. Ahankari AS, Kabra P, Tata LJ, et al. (2020) Two measures of systemic inflammation are positively associated with haemoglobin levels in adolescent girls living in rural India: a cross-sectional study. *Tropical medicine & international health : TM & IH.* **09**.

7. Ahankari AS, Myles PR, Fogarty AW, et al. (2017) Prevalence of iron-deficiency anaemia and risk factors in 1010 adolescent girls from rural Maharashtra, India: a cross-sectional survey. *Public Health* **142**, 159–166.

8. Ahankari AS, Tata LJ & Fogarty AW (2020) Weight, height, and midupper arm circumference are associated with haemoglobin levels in adolescent girls living in rural India: a cross-sectional study. *Maternal and Child Nutrition* **16**.

9. Ahmad MS, Zaidi SAH, Medhat N, et al. (2018) Frequency of underweight and stunting among children entering school in a small urban locality and their association with academic performance. *Journal of the Pakistan Medical Association* **68**, 28–32.

10. Ahmed D (2017) Risk factors for cardiovascular diseases among urban schoolchildren from affluent families. *Bangladesh Medical Research Council Bulletin* **43**, 26–30.

11. Akram S, Khan MA, Usman HB, et al. (2017) Assessment of the nutritional status of primary school children in Shangla. *Pakistan Paediatric Journal* **41**, 9–13.

12. Al Ani MF, Al Subhi LK & Bose S (2016) Consumption of fruits and vegetables among adolescents: a multi-national comparison of eleven countries in the Eastern Mediterranean Region. *Br J Nutr* **115**, 1092–9.

13. Allen A, Allen S, Rodrigo R, et al. (2017) Iron status and anaemia in Sri Lankan secondary school children: a cross-sectional survey. *PLoS ONE* **12**, e0188110.

14. Amarasinghe GS, Naottunna NPGCR, Agampodi TC, et al. (2017) Factors associated with anemia among Sri Lankan primary school children in rural North Central Province. *BMC Pediatrics* **17**.

15. Anitha S, Kane-Potaka J, Tsusaka TW, et al. (2019) Acceptance and impact of millet-based mid-day meal on the nutritional status of adolescent school going children in a peri urban region of Karnataka State in India. *Nutrients* **11**, 03.

16. Ansari MA & Khan Z (2017) Biochemical iodine deficiency in selected schools of aligarh. *Indian J Public Health* **61**, 309–311.

17. Anusha AS, Gopalakrishnan S, Savitha AK, et al. (2018) Evaluation of goitre and its sociodemographic risk factors among rural school children of Kancheepuram, Tamil Nadu, India. *Journal of Clinical and Diagnostic Research* **12**, LC10–LC14.

18. Arora B, Patel SS & Saboo B (2019) Risk factors associated with overweight and obesity among population of Ahmedabad, India. *Indian Journal of Public Health Research and Development* **10**, 168–172.

19. Ashraf H, Shamsi NI & Ashraf R (2017) Parental perception and childhood obesity: contributors to incorrect perception. *JPMA J Pak Med Assoc* **67**, 214–219.

20. Ashtekar SV, Padhyegurjar MS & Powar J (2019) Protein calorie intakes and growth profiles in ashram school students in Nashik district in Maharashtra. *Indian J Public Health* **63**, 341–347.

21. Asif M, Aslam M & Altaf S (2018) Mid-upper-arm circumference as a screening measure for identifying children with elevated body mass index: a study for Pakistan. *Korean Journal of Pediatrics* **61**, 6–11.

22. Asif M, Aslam M & Altaf S (2018) Use of mid-upper arm circumference in evaluation of overweight and obesity in the Pakistani children and adolescent, aged 12-18 years. *Pakistan Paediatric Journal* **42**, 43–48.

23. Asif M, Aslam M, Wyszynska J, et al. (2020) Diagnostic performance of neck circumference and cut-off values for identifying overweight and obese Pakistani children: a receiver operating characteristic analysis. *Journal of clinical research in pediatric endocrinology.* **16**.

24. Aslami AN, Ansari MA, Khalique N, et al. (2016) Iodine deficiency in school children in Aligarh district, India. *Indian Pediatr* **53**, 742–3.

25. Aurino E (2017) Do boys eat better than girls in India? Longitudinal evidence on dietary diversity and food consumption disparities among children and adolescents. *Econ Hum Biol* **25**, 99–111.

26. Aurino E, Fernandes M & Penny ME (2017) The nutrition transition and adolescents’ diets in low- and middle-income countries: a cross-cohort comparison. *Public Health Nutr* **20**, 72–81.

27. Aziz A, Pervaiz M, Khalid A, et al. (2018) Dietary practices of school children in Sindh, Pakistan. *Nutr Health* **24**, 231–240.

28. Azizi S & Tariq TM (2019) Vitamin D deficiency among Afghan adolescents in Kabul. *J Coll Physicians Surg Pak* **29**, 1072–1077.

29. Balaram S & Dipayan C (2017) Nutritional status of adolescent of Tripura-a North eastern state of India. *Biomedicine (India)* **37**, 274–278.

30. Bali S, Singh AR & Nayak PK (2018) Iodine deficiency and toxicity among school children in Damoh district, Madhya Pradesh, India. *Indian Pediatr* **55**, 579–581.

31. Bali S, Tomar A, Nayak PK, et al. (2019) Goitre is no longer prevalent and urinary iodine excretion is above normal among school going children in Jabalpur, India: is this major health problem already solved? *J Trop Pediatr* **65**, 457–462.

32. Banik R, Naher S, Pervez S, et al. (2020) Fast food consumption and obesity among urban college going adolescents in Bangladesh: A cross-sectional study. *Obesity Medicine* **17 (no pagination)**.

33. Bansal PG, Toteja GS, Bhatia N, et al. (2016) Impact of weekly iron folic acid supplementation with and without vitamin B12 on anaemic adolescent girls: a randomised clinical trial. *Eur J Clin Nutr* **70**, 730–7.

34. Bansal PG, Toteja GS, Bhatia N, et al. (2016) Comparison of haemoglobin estimates using direct & indirect cyanmethaemoglobin methods. *Indian J Med Res* **144**, 566–571.

35. Bawaskar HS & Bawaksar PH (2020) Profile of Vitamin B12 and Vitamin D in Rural Schoolchildren in Raigad, India. *Indian Pediatrics* **57**, 871.

36. Bellizzi S, Pichierri G, Panu Napodano CM, et al. (2020) Iron deficiency anemia and low Body Mass Index among adolescent girls in India, the transition from 2005 to 2015. *Public health nutrition*, 1–19.

37. Bhargava M, Bhargava A, Ghate SD, et al. (2020) Nutritional status of Indian adolescents (15-19 years) from National Family Health Surveys 3 and 4: revised estimates using WHO 2007 Growth reference. *PLoS ONE* **15**, e0234570.

38. Bhargava M, Kandpal SD, Aggarwal P, et al. (2016) Overweight and obesity in school children of a hill state in north India: is the dichotomy urban-rural or socio-economic? Results from a cross-sectional survey. *PLoS ONE* **11**, e0156283.

39. Bhattacharya U & Chandra AK (2019) Assessment of iodine nutritional status of school-age children in Kolkata district of West Bengal State in post-iodation scenario. *J Trop Pediatr* **65**, 55–62.

40. Bhattacharyya H, Nath CK, Pala S, et al. (2020) Iodine deficiency disorders in children in East Khasi Hills district of Meghalaya, India. *Indian Pediatrics* **57**, 811–814.

41. Bhuvaneswari B & Parameshwari S (2020) Potential factors related to bmi among school going adolescents of Madurai district, Tamil Nadu, India. *International Journal of Research in Pharmaceutical Sciences* **11**, 5448–5452.

42. Bhuvaneswari G (2020) A study to assess the effectiveness of honey dates amla mix on biochemical markers among adolescent girls with iron deiciency anaemia. *International journal of research in pharmaceutical sciences* **11**, 2288‐2293.

43. Biswas T, Islam A, Islam MS, et al. (2017) Overweight and obesity among children and adolescents in Bangladesh: a systematic review and meta-analysis. *Public Health* **142**, 94–101.

44. Biswas T, Magalhaes RJS, Townsend N, et al. (2020) Double burden of underweight and overweight among women in south and southeast Asia: a systematic review and meta-analysis. *Advances in nutrition (Bethesda, Md.)* **11**, 128–143.

45. Caleyachetty R, Thomas GN, Kengne AP, et al. (2018) The double burden of malnutrition among adolescents: analysis of data from the Global School-Based Student Health and Health Behavior in School-Aged Children surveys in 57 low- and middle-income countries. *American Journal of Clinical Nutrition* **108**, 414–424.

46. Campbell RK, Aguayo VM, Kang Y, et al. (2018) Epidemiology of anaemia in children, adolescent girls, and women in Bhutan. *Matern Child Nutr* **14 Suppl 4**, e12740.

47. Campisi SC, Humayun KN, Soder O, et al. (2020) Later puberty onset among chronically undernourished adolescents living in a Karachi slum, Pakistan. *Journal of Adolescent Health* **66 (2 Supplement)**, S65.

48. Campisi SC, Humayun KN, Wasan Y, et al. (2020) Self-assessed puberty is reliable in a low-income setting in rural Pakistan. *Journal of Pediatric Endocrinology and Metabolism* **33**, 1191–1196.

49. Chakraborty S, Chopra M, Mani K, et al. (2018) Prevalence of vitamin B12 deficiency in healthy Indian school-going adolescents from rural and urban localities and its relationship with various anthropometric indices: a cross-sectional study. *Journal of human nutrition and dietetics : the official journal of the British Dietetic Association* **31**, 513–522.

50. Chakraborty S, Prasad G, Marwaha RK, et al. (2020) Comparison of plasma adipocytokines & C-reactive protein levels in healthy schoolgoing adolescents from private & government-funded schools of Delhi, India. *Indian Journal of Medical Research* **151**, 47–58.

51. Chalise B, Aryal KK, Mehta RK, et al. (2018) Prevalence and correlates of anemia among adolescents in Nepal: findings from a nationally representative cross-sectional survey. *PLoS ONE* **13**, e0208878.

52. Chaput JP, Barnes JD, Tremblay MS, et al. (2018) Thresholds of physical activity associated with obesity by level of sedentary behaviour in children. *Pediatric Obesity* **13**, 450–457.

53. Chattopadhyay A, Sethi V, Nagargoje VP, et al. (2019) WASH practices and its association with nutritional status of adolescent girls in poverty pockets of eastern India. *BMC Womens Health* **19**, 89.

54. Chiplonkar S, Kajale N, Ekbote V, et al. (2017) Reference centile curves for body fat percentage, fat-free mass, muscle mass and bone mass measured by bioelectrical impedance in Asian Indian children and adolescents. *Indian Pediatrics* **54**, 1005–1011.

55. Choudhary R, Sharma R, Bhat M, et al. (2017) Prevalence of dental caries in overweight school going children of 12-15 years in and around Jaipur city, Rajasthan, India. *Przegl Epidemiol* **71**, 623–628.

56. Choudhary S, Khichar S, Dabi D, et al. (2016) Urban rural comparison of anthropometry and menarcheal status of adolescent school going girls of Jodhpur, Rajasthan, India. *Journal of Clinical and Diagnostic Research* **10**, SC08-SC12.

57. Choudhuri D & Balaram S (2020) Factors associated with nutritional status of adolescent schoolchildren in Tripura. *Indian Pediatrics* **57**, 177–178.

58. Cunningham K, Pries A, Erichsen D, et al. (2020) Adolescent girls’ nutritional status and knowledge, beliefs, practices, and access to services: an assessment to guide intervention design in Nepal. *Current Developments in Nutrition* **4**.

59. Darling AM, Fawzi WW, Barik A, et al. (2020) Double burden of malnutrition among adolescents in rural West Bengal, India. *Nutrition* **79-80 (no pagination)**.

60. Debnath M, Tigga PL, Mondal N, et al. (2016) Birth order, father’s occupation and family size are strongly associated with thinness among bengalee adolescent girls of Darjeeling district, West Bengal (India). *Journal of Nepal Paediatric Society* **36**, 115–120.

61. Devara R & Deshmukh D (2017) Impact of nutritious meals on the nutritional status of the tribal students: a comparison between centralized kitchens (Annapurna) and regular kitchens in government tribal residential schools from two Districts of Maharashtra, India. *Indian J Public Health* **61**, 233–238.

62. Dhungana RR, Bista B, Pandey AR, et al. (2019) Prevalence, clustering and sociodemographic distributions of non-communicable disease risk factors in Nepalese adolescents: secondary analysis of a nationwide school survey. *BMJ Open* **9**, e028263.

63. Didzun O, De Neve JW, Awasthi A, et al. (2019) Anaemia among men in India: a nationally representative cross-sectional study. *Lancet Glob Health* **7**, e1685–e1694.

64. Din JU, Yousafzai AM, Khan RA, et al. (2019) Iron deficiency anaemia in school age children of district Tank Khyber, Pakhtunkhwa province, Pakistan. *JPMA J Pak Med Assoc* **69**, 1543–1546.

65. Dolkar T, Mehta BVK & Wangdi JT (2019) A study on the nutritional status of the school going adolescents of East Sikkim, north east India. *Indian Journal of Public Health Research and Development* **10**, 232–237.

66. Dutta A, Mohapatra MK, Rath M, et al. (2020) Effect of caste on health, independent of economic disparity: evidence from school children of two rural districts of India. *Sociology of health & illness* **42**, 1259–1276.

67. Dutta M, Selvamani Y, Singh P, et al. (2019) The double burden of malnutrition among adults in India: evidence from the National Family Health Survey-4 (2015-16). *Epidemiol Health* **41**, e2019050.

68. Ekbote VH, Khadilkar AV, Khadilkar VV, et al. (2017) Dietary patterns with special reference to calcium intake in 2-16-year-old urban western Indian children. *Indian J Public Health* **61**, 188–193.

69. Eroglu A, Schulze KJ, Yager J, et al. (2018) Plasma proteins associated with circulating carotenoids in Nepalese school-aged children. *Arch Biochem Biophys* **646**, 153–160.

70. Eshwar TK, Chudasama RK, Eshwar ST, et al. (2017) Prevalence of obesity and overweight and their comparison by three growth standards among affluent school students aged 8-18 years in Rajkot. *Indian J Public Health* **61**, 51–54.

71. Faizi N, Khan Z, Khan IM, et al. (2017) A study on nutritional status of school-going adolescents in Aligarh, India. *Tropical Doctor* **47**, 212–216.

72. Fan H & Zhang X (2020) Clustering of poor dietary habits among adolescents aged 12 to 15 years in 52 low-income and middle-income countries. *International Journal of Environmental Research and Public Health* **17**, 1–12.

73. Ferdous F, Raqib R, Ahmed S, et al. (2020) Early childhood malnutrition trajectory and lung function at preadolescence. *Public health nutrition*, 1–12.

74. Field MS, Mithra P, Estevez D, et al. (2020) Wheat flour fortification with iron for reducing anaemia and improving iron status in populations. *Cochrane Database of Systematic Reviews*.

75. Ford ND, Bichha RP, Parajuli KR, et al. (2020) Factors associated with anaemia among adolescent boys and girls 10-19 years old in Nepal. *Maternal and Child Nutrition.*

76. Galgamuwa LS, Iddawela D, Dharmaratne SD, et al. (2017) Nutritional status and correlated socio-economic factors among preschool and school children in plantation communities, Sri Lanka. *BMC Public Health* **17**, 377.

77. Gamage AU & Jayawardana PL (2017) Knowledge of non-communicable diseases and practices related to healthy lifestyles among adolescents, in state schools of a selected educational division in Sri Lanka. *BMC Public Health* **18**, 64.

78. Ganesan S, Chacko TV & Muhammad GM (2019) Are our rural adolescents eating healthy? Implications for redesigning school health interventions - a cross sectional study in rural Coimbatore. *Indian J Public Health* **63**, 293–297.

79. Ganie MA, Bhat GA, Wani IA, et al. (2017) Prevalence, risk factors and consequences of overweight and obesity among schoolchildren: a cross-sectional study in Kashmir, India. *J Pediatr Endocrinol Metab* **30**, 203–209.

80. Gausman J, Kim R & Subramanian SV (2019) Stunting trajectories from post-infancy to adolescence in Ethiopia, India, Peru, and Vietnam. *Matern Child Nutr* **15**, e12835.

81. George N, Johnson AR, Lobo A, et al. (2018) Health problems and health seeking behavior among school-going adolescents in a rural area in south Karnataka. *Journal of Indian Association for Child and Adolescent Mental Health* **14**, 50–65.

82. Goel M, Pal P, Agrawal A, et al. (2016) Relationship of body mass index and other life style factors with hypertension in adolescents. *Annals of Pediatric Cardiology* **9**, 29–34.

83. Goonapienuwala BL, Agampodi SB, Kalupahana NS, et al. (2019) Body image perception and body dissatisfaction among rural Sri Lankan adolescents; do they have a better understanding about their weight? *Ceylon Med J* **64**, 82–90.

84. Greene-Cramer B, Harrell MB, Hoelscher DM, et al. (2018) Association between parent and child weight status among private school children in Delhi, India. *Glob Health Promot* **25**, 67–74.

85. Greksa LP, Islam ABMR, Okamoto R, et al. (2017) Dietary patterns and dietary adequacy of street children in Dhaka, Bangladesh. *Ecology of food and nutrition* **56**, 479–492.

86. Gupta A, Kapil U & Singh G (2018) Consumption of junk foods by school-aged children in rural Himachal Pradesh, India. *Indian J Public Health* **62**, 65–67.

87. Gupta P, Raizada N, Giri S, et al. (2020) Goiter prevalence and thyroid autoimmunity in school children of Delhi. *Indian Journal of Endocrinology and Metabolism* **24**, 202–205.

88. Gupta S, Gupta A, Raina B, et al. (2017) Prevalence and pattern of anaemias in children at ASCOMS & hospital Jammu. *JK Science* **19**, 76–80.

89. Habib N, Abbasi SURS & Aziz W (2020) An analysis of societal determinant of anemia among adolescent girls in Azad Jammu and Kashmir, Pakistan. *Anemia* **2020 (no pagination)**.

90. Hambidge KM, Krebs NF, Garces A, et al. (2017) Anthropometric indices for non-pregnant women of childbearing age differ widely among four low-middle income populations. *BMC Public Health* **18**, 45.

91. Harding KL, Aguayo VM & Webb P (2019) Trends and correlates of overweight among pre-school age children, adolescent girls, and adult women in south Asia: an analysis of data from twelve national surveys in six countries over twenty years. *Nutrients* **11**, 14.

92. Hassan F, Asim M, Salim S, et al. (2017) House ownership, frequency of illness, fathers’ education: the most significant socio-demographic determinants of poor nutritional status in adolescent girls from low income households of Lahore, Pakistan. *Intern* **16**, 122.

93. Hassan MT, Das H & Banik S (2020) A cross-sectional study to determine the prevalence of overweight and obesity among Bangladeshi adolescents based on WHO, IOTF, and CDC cut-points. *Obesity Medicine* **19 (no pagination)**.

94. Hemamalini K & Lalitha Kumari B (2016) Assessment of nutritional status of boys and girls in government and corporate school children in Nambur mandal, Guntur (Dt.) Andhra Pradesh, India. *Research Journal of Pharmaceutical, Biological and Chemical Sciences* **7**, 1054–1062.

95. Higgins-Steele A, Mustaphi P, Varkey S, et al. (2016) Stop stunting: situation and way forward to improve maternal, child and adolescent nutrition in Afghanistan. *Matern Child Nutr* **12 Suppl 1**, 237–41.

96. Humphries DL, Dearden KA, Crookston BT, et al. (2017) Household food group expenditure patterns are associated with child anthropometry at ages 5, 8 and 12 years in Ethiopia, India, Peru and Vietnam. *Economics and Human Biology* **26**, 30–41.

97. Iqbal M, Fatmi Z, Khan K, et al. (2020) Malnutrition and food insecurity in child labourers in Sindh, Pakistan: a cross-sectional study. *Eastern Mediterranean Health Journal* **26**, 1087–1096.

98. Islam MR, Rahman SM, Tarafder C, et al. (2020) Exploring rural adolescents’ dietary diversity and its socioeconomic correlates: a cross-sectional study from Matlab, Bangladesh. *Nutrients* **12**, 1–16.

99. Islam TMM, Banik PC, Barua L, et al. (2020) Cardiovascular disease risk factors among school children of Bangladesh: a cross-sectional study. *BMJ Open* **10**, e038077.

100. Jayalakshmi R & Jissa VT (2017) Nutritional status of mid-day meal programme beneficiaries: a cross-sectional study among primary schoolchildren in Kottayam district, Kerala, India. *Indian J Public Health* **61**, 86–91.

101. Jayatissa R, Gorstein J, Okosieme OE, et al. (2020) Stable iodine nutrition during two decades of continuous universal salt iodisation in Sri Lanka. *Nutrients* **12**.

102. Jayatissa R, Lekamwasam S, Ranbanda JM, et al. (2019) Vitamin D deficiency among children aged 10-18 years in Sri Lanka. *Ceylon Med J* **64**, 146–154.

103. Jayawardena R, Ranasinghe P, Wijayabandara M, et al. (2017) Nutrition transition and obesity among teenagers and young adults in south Asia. *Curr Diabetes Rev* **13**, 444–451.

104. Jeevanandam S & Prathibha KM (2019) Measurement of 2D:4D ratio and neck circumference in adolescents: sexual dimorphism and its implications in obesity-a cross sectional study. *Indian Journal of Endocrinology and Metabolism* **22**, 724–727.

105. Jeyakumar A & Ghugre P (2017) Is lack of breakfast contributing to nutrient deficits and poor nutritional indicators among adolescent girls? *Nutr Health* **23**, 177–184.

106. Jeyakumar A & Shinde V (2019) A systematic review and meta-analysis of prevalence of vitamin D deficiency among adolescent girls in selected Indian states. *Nutr Health* **25**, 61–70.

107. Kajale NA, Mughal Z, Khadilkar V, et al. (2016) Association of dietary calcium intake and body fat with hypertension in Indian adolescents. *Indian Pediatr* **53**, 434–5.

108. Kapil U, Pandey R, Goswami R, et al. (2017) Prevalence of Vitamin D deficiency and associated risk factors among children residing at high altitude in Shimla district, Himachal Pradesh, India. *Indian Journal of Endocrinology and Metabolism* **21**, 178–183.

109. Kapil U, Pandey RM, Sharma B, et al. (2018) Prevalence of vitamin D deficiency in children (6-18 years) residing in Kullu and Kangra districts of Himachal Pradesh, India. *Indian J Pediatr* **85**, 344–350.

110. Kapil U, Sareen N, Nambiar VS, et al. (2016) Iodine nutritional status among adolescent girls in Uttarakhand, India. *J Trop Pediatr* **62**, 81–2.

111. Kapoor A, Channa NA, Soomro AM, et al. (2018) Malnutrition and clinical manifestations in school going children at district Tharparkar, Sindh, Pakistan. *Rawal Medical Journal* **43**, 115–119.

112. Karageorgou D, Imamura F, Zhang J, et al. (2018) Assessing dietary intakes from household budget surveys: a national analysis in Bangladesh. *PLoS ONE* **13**, e0202831.

113. Karki A, Shrestha A & Subedi N (2019) Prevalence and associated factors of childhood overweight/obesity among primary school children in urban Nepal. *BMC Public Health* **19**, 1055.

114. Karki S, Pakkila J, Ryhanen T, et al. (2019) Body mass index and dental caries experience in Nepalese schoolchildren. *Community Dent Oral Epidemiol* **47**, 346–357.

115. Khadgawat R, Marwaha RK, Mehan N, et al. (2016) Age of onset of puberty in apparently healthy school girls from northern India. *Indian Pediatr* **53**, 383–7.

116. Khan A, Khan SR & Burton NW (2019) Missing breakfast is associated with overweight and obesity in Bangladeshi adolescents. *Acta Paediatr* **108**, 178–179.

117. Khan MMA, Karim M, Islam AZ, et al. (2019) Prevalence of overweight and obesity among adolescents in Bangladesh: do eating habits and physical activity have a gender differential effect? *J Biosoc Sci* **51**, 843–856.

118. Khan SA, Pokharel B, Bhagat T, et al. (2020) Prevalence of vitamin D insufficiency in school going children of eastern Nepal: a cross-sectional study. *International Medicine* **2**, 214–221.

119. Khatiwada S, Gelal B, Baral N, et al. (2016) Association between iron status and thyroid function in Nepalese children. *Thyroid Research* **9**.

120. Khongrangjem T, Dsouza SM, Prabhu P, et al. (2018) A study to assess the knowledge and practice of fast food consumption among pre-university students in Udupi Taluk, Karnataka, India. *Clinical Epidemiology and Global Health* **6**, 172–175.

121. Krishna C, Sagar MK, Iyengar K, et al. (2020) Food habits and physical activity among adolescent medical students of a medical college in Tumkur, Karnataka, India. *Indian Journal of Public Health Research and Development* **11**, 180–185.

122. Krishna C, Venkatesh P, Iyengar K, et al. (2020) Anaemia and its determinants among adolescent medical students of a health university in Tumkur, Karnataka, India. *Indian Journal of Public Health Research and Development* **11**, 186–191.

123. Kujur A, Kumar D, Kumar C, et al. (2020) Socio-demographic differentials of cognitive development and nutrition among school children: evidence from the tribal areas of Jharkhand, India. *International Journal of Adolescent Medicine and Health* **(no pagination)**.

124. Kumar KJ, Saldanha K, Sushma K, et al. (2017) A prospective study of homocysteine and its relation to body mass index and lipid profile in school children. *Indian Pediatr* **54**, 935–937.

125. Kumar MV & Erhardt J (2020) Improving the iron status of school children through a school noon meal programme with meals prepared using a multiple micronutrient-fortified salt in Tamil Nadu, India. *Asia Pacific journal of clinical nutrition* **29**, 577–583.

126. Kumar P, Kumar D, Ranjan A, et al. (2017) Prevalence of hypertension and its risk factors among school going adolescents of Patna, India. *Journal of Clinical and Diagnostic Research* **11**, SC01–SC04.

127. Kumar P, Singh RK, Mahalingam K, et al. (2020) Anthropometric and biochemical analysis of normal and obese subjects on an Indian population. *International Journal of Pharmaceutical Sciences and Research* **11**, 1152–1160.

128. Kumar S, Kroon J, Lalloo R, et al. (2017) Relationship between body mass index and dental caries in children, and the influence of socio-economic status. *Int Dent J* **67**, 91–97.

129. Kumar S, Ray S, Roy D, et al. (2017) Exercise and eating habits among urban adolescents: a cross-sectional study in Kolkata, India. *BMC Public Health* **17**, 468.

130. Kumari R, Bharti RK, Singh K, et al. (2017) Prevalence of iron deficiency and iron deficiency anaemia in adolescent girls in a tertiary care hospital. *Journal of Clinical and Diagnostic Research* **11**, BC04–BC06.

131. Kunwar R, Minhas S & Mangla V (2018) Is obesity a problem among school children? *Indian J Public Health* **62**, 153–155.

132. Lahiri A, Chakraborty A, Dasgupta U, et al. (2019) Effect of dietary habit and physical activity on overnutrition of schoolgoing adolescents: a longitudinal assessment in a rural block of West Bengal. *Indian J Public Health* **63**, 171–177.

133. Leyvraz M, Laillou A, Rahman S, et al. (2016) An assessment of the potential impact of fortification of staples and condiments on micronutrient intake of young children and women of reproductive age in Bangladesh. *Nutrients* **8**, 02.

134. Li L, Sun N, Zhang L, et al. (2020) Fast food consumption among young adolescents aged 12-15 years in 54 low- and middle-income countries. *Global health action* **13**, 1795438.

135. Li W, Liu E & BeLue R (2018) Household water treatment and the nutritional status of primary-aged children in India: findings from the India human development survey. *Global health* **14**, 37.

136. MacWana JI, Mehta KG & Baxi RK (2017) Predictors of overweight and obesity among school going adolescents of Vadodara city in western India. *International Journal of Adolescent Medicine and Health* **29**.

137. Majid H, Khawaja S, Khan AH, et al. (2017) Burden of zinc (Zn) deficiency: a high volume clinical laboratory data analysis. *JPMA J Pak Med Assoc* **67**, 1593–1595.

138. Manandhar N, Pathak P, Lama P, et al. (2020) Morbidity of school children in Panauti municipality of Kavrepalanchowk: a descriptive cross-sectional study. *Journal of the Nepal Medical Association* **58**, 230–233.

139. Manandhar S, Suksaroj TT & Rattanapan C (2019) The association between green space and the prevalence of overweight/obesity among primary school children. *Int* **10**, 1–10.

140. Mandlik R, Kajale N, Ekbote V, et al. (2018) Determinants of vitamin D status in Indian school-children. *Indian Journal of Endocrinology and Metabolism* **22**, 244–248.

141. Mandlik R, Khadilkar A, Kajale N, et al. (2018) Response of serum 25(OH)D to vitamin D and calcium supplementation in school-children from a semi-rural setting in India. *J Steroid Biochem Mol Biol* **180**, 35–40.

142. Mansoori N, Nisar N, Shahid N, et al. (2018) Prevalence of obesity and its risk factors among school children in Karachi, Pakistan. *Trop Doct* **48**, 266–269.

143. Marwaha RK, Garg MK, Mithal A, et al. (2019) Effect of Vitamin D supplementation on bone turnover markers in children and adolescents from North India. *Indian Journal of Endocrinology and Metabolism* **23**, 27–34.

144. Marwaha RK, Garg MK, Sethuraman G, et al. (2019) Impact of three different daily doses of vitamin D3 supplementation in healthy schoolchildren and adolescents from North India: a single-blind prospective randomised clinical trial. *British Journal of Nutrition* **121**, 538–548.

145. Mia MN, Rahman MS & Roy PK (2018) Sociodemographic and geographical inequalities in under- and overnutrition among children and mothers in Bangladesh: a spatial modelling approach to a nationally representative survey. *Public Health Nutr* **21**, 2471–2481.

146. Mohan B, Verma A, Singh K, et al. (2019) Prevalence of sustained hypertension and obesity among urban and rural adolescents: a school-based, cross-sectional study in north India. *BMJ Open* **9**.

147. Mohsin SN, Fatima M & Aasim M (2016) Outcome of iron deficiency on the global school performance of the children: a case study from Nowshera, Pakistan. *Pakistan Journal of Medical and Health Sciences* **10**, 1116–1121.

148. Moonajilin MS, Rahman ME & Islam MS (2020) Relationship between overweight/obesity and mental health disorders among Bangladeshi adolescents: a cross-sectional survey. *Obesity Medicine* **18 (no pagination)**.

149. Muhammad GM, Ganesan S & Chacko TV (2019) Effectiveness of multi-strategic health screening cum educational intervention model in promoting health of school children in rural Coimbatore. *Indian J Public Health* **63**, 133–138.

150. Naotunna NP, Dayarathna M, Maheshi H, et al. (2017) Nutritional status among primary school children in rural Sri Lanka; a public health challenge for a country with high child health standards. *BMC Public Health* **17**, 57.

151. Nasreddine L, Ayoub JJ & Al Jawaldeh A (2018) Review of the nutrition situation in the eastern Mediterranean region. *East Mediterr Health J* **24**, 77–91.

152. Nayak BS & Bhat VH (2016) School based multicomponent intervention for obese children in Udupi district, south India - a randomized controlled trial. *Journal of clinical and diagnostic research* **10**, SC24‐SC28.

153. Nayani AA, Iqbal R, Azam SI, et al. (2018) Association between environmental tobacco smoke and dental caries amongst 5-14 years old children in Karachi, Pakistan. *JPMA J Pak Med Assoc* **68**, 203–209.

154. NCR Risk Factor Collaboration (2017) Worldwide trends in body-mass index, underweight, overweight, and obesity from 1975 to 2016: a pooled analysis of 2416 population-based measurement studies in 128.9 million children, adolescents, and adults. *Lancet* **390**, 2627–2642.

155. Nithya DJ & Bhavani RV (2018) Dietary diversity and its relationship with nutritional status among adolescents and adults in rural India. *J Biosoc Sci* **50**, 397–413.

156. Nunn RL, Kehoe SH, Chopra H, et al. (2019) Dietary micronutrient intakes among women of reproductive age in Mumbai slums. *Eur J Clin Nutr* **73**, 1536–1545.

157. Pal A, Pari AK, Sinha A, et al. (2017) Prevalence of undernutrition and associated factors: a cross-sectional study among rural adolescents in West Bengal, India. *International Journal of Pediatrics and Adolescent Medicine* **4**, 9–18.

158. Pandey A & Sapkota S (2018) Prevalence and knowledge on obesity among school going adolescents of Kaski, Nepal. *Journal of Nepal Paediatric Society* **38**, 63–68.

159. Pareek R & Ojha NK (2018) Study of prevalence of iron deficiency anemia in adolescent girls in Jaipur district, India. *International Journal of Research in Ayurveda and Pharmacy* **9**, 181–185.

160. Parray IA, Parry MA & Latief M (2016) Prevalence of dyslipidemia in school children of Kashmir valley. *Diabetes Metab Syndr* **10**, S47-54.

161. Pawar PV, Kanetkar SR, Deo MG, et al. (2020) Anemia-tribe-specific study and it’s sociodemographic association in six dominant tribal adolescents of Maharashtra, India. *International Journal of Pharmaceutical Research* **12**, 510–519.

162. Pawar SV, Choksey AS, Jain SS, et al. (2016) Prevalence of overweight and obesity in 4 schools of south Mumbai. *Journal of Clinical and Diagnostic Research* **10**, OC01–OC02.

163. Pawar SV, Zanwar VG, Choksey AS, et al. (2016) Most overweight and obese Indian children have nonalcoholic fatty liver disease. *Ann Hepatol* **15**, 853–861.

164. Pinni J, Avula JSS & Bandi S (2019) Association of dental caries with socio-demographic and nutritional factors among school children in Guntur district of Andhra Pradesh, India. *Pediatric Dental Journal* **29**, 111–115.

165. Piryani S, Baral KP, Pradhan B, et al. (2016) Overweight and its associated risk factors among urban school adolescents in Nepal: a cross-sectional study. *BMJ Open* **6**, e010335.

166. Pramod JB & Narayan TD (2019) Prevalence of lifestyle related risk factors for non-communicable diseases among adolescents of an urban community in Mumbai. *Indian Journal of Public Health Research and Development* **10**, 242–247.

167. Prasad R, Bazroy J & Singh Z (2016) Prevalence of overweight and obesity among adolescent students in Pondicherry, south India. *International Journal of Nutrition, Pharmacology, Neurological Diseases* **6**, 72–75.

168. Radhika MS, Swetha B, Kumar BN, et al. (2018) Dietary and nondietary determinants of nutritional status among adolescent girls and adult women in India. *Annals of the New York Academy of Sciences* **1416**, 5–17.

169. Rahman AS, Ahmed T, Ahmed F, et al. (2015) Double-blind cluster randomised controlled trial of wheat flour chapatti fortified with micronutrients on the status of vitamin A and iron in school-aged children in rural Bangladesh. *Maternal & child nutrition* **11 Suppl 4**, 120‐131.

170. Rahman MA, Rahman MM, Rahman MM, et al. (2019) The double burden of under- and overnutrition among Bangladeshi women: socioeconomic and community-level inequalities. *PLoS ONE* **14**, e0219968.

171. Rahman M & Mistry S (2017) Anemia and its socio-demographic correlates among adolescent girls in Bangladesh. *Annals of Global Health* **83 (1)**, 133.

172. Rahman S, Ahmed T, Rahman AS, et al. (2016) Determinants of iron status and Hb in the Bangladesh population: the role of groundwater iron. *Public Health Nutr* **19**, 1862–74.

173. Rahman S, Rahman AS, Alam N, et al. (2017) Vitamin A deficiency and determinants of vitamin A status in Bangladeshi children and women: findings of a national survey. *Public Health Nutr* **20**, 1114–1125.

174. Rai A, Gurung S, Thapa S, et al. (2019) Correlates and inequality of underweight and overweight among women of reproductive age: evidence from the 2016 Nepal Demographic Health Survey. *PLoS ONE* **14**, e0216644.

175. Rai A, Khan MN & Thapa S (2020) Trends and determinants of anaemia in women of Nepal: a multilevel analysis. *Maternal and Child Nutrition* **16**.

176. Rakesh PS (2017) Prevalence of anaemia in Kerala state, southern India - a systematic review. *Journal of Clinical and Diagnostic Research* **11**, LE01–LE04.

177. Rakesh PS, George LS, Joy TM, et al. (2019) Anemia among school children in Ernakulam district, Kerala, India. *Indian Journal of Hematology and Blood Transfusion* **35**, 114–118.

178. Ramirez-Luzuriaga MJ, Larson LM, Mannar V, et al. (2018) Impact of double-fortified salt with iron and iodine on hemoglobin, anemia, and iron deficiency anemia: a systematic review and meta-analysis. *Adv Nutr (Bethesda)* **9**, 207–218.

179. Ramkumar S, Vijayalakshmi S, Kanagarajan P, et al. (2018) Z-score and CIAF-a descriptive measure to determine prevalence of under-nutrition in rural school children, Puducherry, India. *Journal of Clinical and Diagnostic Research* **12**, LC24–LC27.

180. Ranjani H, Mehreen TS, Pradeepa R, et al. (2016) Epidemiology of childhood overweight & obesity in India: a systematic review. *Indian J Med Res* **143**, 160–74.

181. Raqib R, Ahmed S, Ahsan KB, et al. (2017) Humoral immunity in arsenic-exposed children in rural Bangladesh: total immunoglobulins and vaccine-specific antibodies. *Environmental Health Perspectives* **125**.

182. Rathi N, Riddell L & Worsley A (2017) Food consumption patterns of adolescents aged 14-16 years in Kolkata, India. *Nutr J* **16**, 50.

183. Rathi N, Riddell L & Worsley A (2018) Indian adolescents’ perceptions of the home food environment. *BMC Public Health* **18**, 169.

184. Rauf A, Nadeem MS, Khalid M, et al. (2018) Low body mass index and trends of tuberculosis infection: a cohort study of orphan children in Azad Jammu and Kashmir Pakistan. *Open Public Health Journal* **11**, 384–392.

185. Resmi S, Latheef F, & Vijayaraghavan (2017) Effectiveness of amla, jaggery and pumpkin leaves extract on the level of haemoglobin, vitamin C and iron among adolescent girls with iron deficiency anemia. *International journal of pharmaceutical sciences and research* **8**, 4812‐4817.

186. Rodrigo R, Allen A, Manampreri A, et al. (2018) Haemoglobin variants, iron status and anaemia in Sri Lankan adolescents with low red cell indices: a cross sectional survey. *Blood Cells Mol Dis* **71**, 11–15.

187. Roman-Vinas B, Chaput JP, Katzmarzyk PT, et al. (2016) Proportion of children meeting recommendations for 24-hour movement guidelines and associations with adiposity in a 12-country study. *International Journal of Behavioral Nutrition and Physical Activity* **13**.

188. Rose-Clarke K, Pradhan H, Rath S, et al. (2019) Adolescent girls’ health, nutrition and wellbeing in rural eastern India: a descriptive, cross-sectional community-based study. *BMC Public Health* **19**, 673.

189. Rousham EK & Khandakar IU (2016) Reducing health inequalities among girls and adolescent women living in poverty: the success of Bangladesh. *Ann Hum Biol* **43**, 115–21.

190. Roy S, Barman S, Mondal N, et al. (2016) Prevalence of stunting and thinness among adolescent girls belonging to the rajbanshi population of West Bengal, India. *Journal of Nepal Paediatric Society* **36**, 147–155.

191. Sabharwal R & Mahajan P (2018) Age, sex and seasonal variations of vitamin D level in children of Jammu region. *Journal of Clinical and Diagnostic Research* **12**, BC09-BC11.

192. Saha M, Adhikary DK, Parvin I, et al. (2018) Obesity and its risk factors of among school children in Sylhet, Bangladesh. *J* **16**, 205–208.

193. Saikia D, Ahmed SJ, Saikia H, et al. (2016) Overweight and obesity in early adolescents and its relation to dietary habit and physical activity: a study in Dibrugarh town. *Clinical Epidemiology and Global Health* **4**, S22–S28.

194. Saikia D, Ahmed SJ, Saikia H, et al. (2018) Body mass index and body fat percentage in assessing obesity: an analytical study among the adolescents of Dibrugarh, Assam. *Indian J Public Health* **62**, 277–281.

195. Sarma D, Saikia UK & Baro A (2019) Vitamin D status of school children in and around Guwahati. *Indian Journal of Endocrinology and Metabolism* **23**, 81–85.

196. Sarna A, Porwal A, Ramesh S, et al. (2020) Characterisation of the types of anaemia prevalent among children and adolescents aged 1-19 years in India: a population-based study. *Lancet Child Adolesc Health* **4**, 515–525.

197. Savanur MS, Sathye A, Udawant A, et al. (2017) Nutritional status and physical fitness of tribal adolescents in Ahmednagar district of Maharashtra. *Ecol food nutr* **56**, 552–566.

198. Schott W, Aurino E, Penny ME, et al. (2019) The double burden of malnutrition among youth: trajectories and inequalities in four emerging economies. *Econ Hum Biol* **34**, 80–91.

199. Scott SP, Murray-Kolb LE, Wenger MJ, et al. (2018) Cognitive performance in Indian school-going adolescents is positively affected by consumption of iron-biofortified pearl millet: a 6-month randomized controlled efficacy trial. *J Nutr* **148**, 1462–1471.

200. Scott S, Pant A, Nguyen PH, et al. (2020) Demographic, nutritional, social and environmental predictors of learning skills and depression in 20,000 Indian adolescents: findings from the UDAYA survey. *PLoS ONE* **15**.

201. Sethi V, Dinachandra K, Murira Z, et al. (2019) Nutrition status of nulliparous married Indian women 15-24 years: decadal trends, predictors and program implications. *PLoS ONE* **14**, e0221125.

202. Sethi V, Gupta N, Pedgaonkar S, et al. (2019) Mid-upper arm circumference cut-offs for screening thinness and severe thinness in Indian adolescent girls aged 10-19 years in field settings. *Public Health Nutr* **22**, 2189–2199.

203. Sethi V, Sternin M, Sharma D, et al. (2017) Applying positive deviance for improving compliance to adolescent anemia control program in tribal communities of India. *Food Nutr Bull* **38**, 447–452.

204. Shahbaz U, Quadir F & Hosein T (2016) Determination of prevalence of dental erosion in 12 - 14 years school children and its relationship with dietary habits. *J Coll Physicians Surg Pak* **26**, 553–6.

205. Shahid B, Jalal MA, Waseem M, et al. (2017) Prevalence of obesity in school going adolescents and its association with hypertension. *Pakistan Journal of Medical and Health Sciences* **11**, 1082–1084.

206. Shaikh NI, Patil SS, Halli S, et al. (2016) Going global: Indian adolescents’ eating patterns. *Public Health Nutr* **19**, 2799–807.

207. Shakya S & Bajracharya S (2019) Hypertension and its determinants among school going adolescents: a cross sectional study in Nepal. *Journal of Nepal Paediatric Society* **39**, 87–94.

208. Sharma S, Akhtar F, Singh RK, et al. (2020) Dietary intake across reproductive life stages of women in India: a cross-sectional survey from 4 districts of India. *Journal of Nutrition and Metabolism* **2020 (no pagination)**.

209. Shetty A, Rao CR, Kamath A, et al. (2019) Goiter prevalence and interrelated components from coastal Karnataka. *Indian J Pediatr* **86**, 159–164.

210. Shinsugi C, Gunasekara D, Gunawardena NK, et al. (2019) Double burden of maternal and child malnutrition and socioeconomic status in urban Sri Lanka. *PLoS ONE* **14**, e0224222.

211. Shinsugi C, Gunasekara D & Takimoto H (2020) Use of mid-upper arm circumference (MUAC) to predict malnutrition among Sri Lankan schoolchildren. *Nutrients* **12**.

212. Shrestha A, Schindler C, Odermatt P, et al. (2018) Intestinal parasite infections and associated risk factors among schoolchildren in Dolakha and Ramechhap districts, Nepal: a cross-sectional study 11 Medical and Health Sciences 1117 Public Health and Health Services. *Parasites and Vectors* **11**.

213. Shrestha A, Schindler C, Odermatt P, et al. (2020) Nutritional and health status of children 15 months after integrated school garden, nutrition, and water, sanitation and hygiene interventions: a cluster-randomised controlled trial in Nepal. *BMC Public Health* **20**, 158.

214. Shridhar K, Millett C, Laverty AA, et al. (2016) Prevalence and correlates of achieving recommended physical activity levels among children living in rural south Asia-a multi-centre study. *BMC Public Health* **16**, 690.

215. Shrivastava P & Maliye C (2019) Status and effectiveness of implementation of weekly iron and folic acid supplementation scheme in schools in central India. *Indian Journal of Public Health Research and Development* **10**, 364–368.

216. Singh JK, Acharya D, Gautam S, et al. (2019) Socio-demographic and diet-related factors associated with insufficient fruit and vegetable consumption among adolescent girls in rural communities of southern Nepal. *Int J Environ Res Public Health* **16**, 17.

217. Siva PM, Sobha A & Manjula VD (2016) Prevalence of anaemia and its associated risk factors among adolescent girls of central Kerala. *Journal of Clinical and Diagnostic Research* **10**, LC19–LC23.

218. Skroder H, Kippler M, De Loma J, et al. (2018) Predictors of selenium biomarker kinetics in 4-9-year-old Bangladeshi children. *Environment International* **Part 1. 121**, 842–851.

219. Som N, Mishra SK & Mukhopadhyay S (2016) Weight concerns and food habits of adolescent girls in two contrasting ecological regions: a comparative study in India. *Eat* **20**, 21–6.

220. Sultana N, Afroz S, Tomalika N, et al. (2019) Prevalence of childhood obesity and undernutrition among urban school children in Bangladesh. *J Biosoc Sci* **51**, 244–253.

221. Talagala IA & Arambepola C (2016) Use of food labels by adolescents to make healthier choices on snacks: a cross-sectional study from Sri Lanka. *BMC Public Health* **16**, 739.

222. Tamang B, Khatiwada S, Gelal B, et al. (2019) Association of antithyroglobulin antibody with iodine nutrition and thyroid dysfunction in Nepalese children. *Thyroid Research* **12**.

223. Tamang MK, Gelal B, Tamang B, et al. (2019) Excess urinary iodine concentration and thyroid dysfunction among school age children of eastern Nepal: a matter of concern. *BMC Res Notes* **12**, 294.

224. Tanwi TS, Chakrabarty S & Hasanuzzaman S (2019) Double burden of malnutrition among ever-married women in Bangladesh: a pooled analysis. *BMC Women’s Health* **19**.

225. Tariq S, Tariq S & Tariq S (2019) Association of perceived stress with healthy and unhealthy food consumption among teenagers. *JPMA J Pak Med Assoc* **69**, 1817–1821.

226. Thakur JS, Bharti B, Tripathy JP, et al. (2016) Impact of 20 week lifestyle intervention package on anthropometric biochemical and behavioral characteristics of schoolchildren in north India. *Journal of Tropical Pediatrics* **62**, 368–376.

227. Thapa B, Powell J, Yi J, et al. (2017) Adolescent Health Risk and Behavior Survey: a school based survey in central Nepal. *Kathmandu Univ* **15**, 301–307.

228. Thapa R, Subedi RK, Regmi G, et al. (2020) Self-reported changes in risk behaviours of cardiovascular diseases among school adolescents in Nepal: application of an integrated experiential learning approach. *Global Heart* **15**.

229. Thorne-Lyman AL, Shaikh S, Mehra S, et al. (2020) Dietary patterns of >30,000 adolescents 9-15 years of age in rural Bangladesh. *Ann N Y Acad Sci* **1468**, 3–15.

230. Twinkle V, Das MC & Kumar VP (2020) Nutritional status of school age children in urban slum area in Vijayawada and Guntur. *Journal of Clinical and Diagnostic Research* **14**, OC01–OC04.

231. Unisa S, Saraswat A, Bhanot A, et al. (2020) Predictors of the diets consumed by adolescent girls, pregnant women and mothers with children under age two years in rural eastern India. *Journal of biosocial science*, 1–20.

232. van Tuijl CJW, Madjdian DS, Bras H, et al. (2020) Sociocultural and economic determinants of stunting and thinness among adolescent boys and girls in Nepal. *Journal of biosocial science*, 1–26.

233. Varkey A, Devi S, Mukhopadhyay A, et al. (2020) Metabolome and microbiome alterations related to short-term feeding of a micronutrient-fortified, high-quality legume protein-based food product to stunted school age children: a randomized controlled pilot trial. *Clinical nutrition (Edinburgh, Scotland)*.

234. Verma M, Sharma P, Khanna P, et al. (2020) Nutrition status of school children in Punjab, India: findings from school health surveys. *Journal of tropical pediatrics.* **01**.

235. Viswanathan VT, Patil SS, Durgawale PM, et al. (2020) Study of prevalence and lifestyle related correlates of overweight and obesity among rural adolescents of western Maharashtra. *International journal of nutrition, pharmacology, neurological diseases* **10**, 29‐34.

236. Viswanathan VT, Patil SS, Patil S, et al. (2020) Assessment of burden of overnutrition and its risk factors among adolescents of rural Maharashtra. *International Journal of Pharmaceutical Research* **12**, 2875–2882.

237. Warnakulasuriya LS, Fernando MAM, Adikaram AVN, et al. (2019) Assessment of nutritional status in Sri Lankan children: validity of current anthropometry cutoffs? *Asia-Pacific journal of public health* **31**, 633–642.

238. Wickramasinghe VP, Arambepola C, Bandara P, et al. (2017) Insulin resistance in a cohort of 5-15 year old children in urban Sri Lanka. *BMC Res Notes* **10**, 347.

239. William RF, Balaji R & Logaraj M (2016) Anaemia and associated factors among school going adolescent girls in Chidambaram, Tamilnadu - a cross sectional study. *Journal International Medical Sciences Academy* **29**, 11–13.

240. Williams J, Townsend N, Rayner M, et al. (2019) Diet quality of adolescents in rural Sri Lanka based on the Diet Quality Index-International: findings from the ‘Integrating Nutrition Promotion and Rural Development’ project. *Public Health Nutrition* **22**, 1735–1744. Cambridge University Press.

241. Wolf RM, Nagpal M & Magge SN (2020) Diabetes and cardiometabolic risk in south Asian youth: a review. *Pediatric Diabetes.*

242. Wray K, Allen A, Evans E, et al. (2017) Hepcidin detects iron deficiency in Sri Lankan adolescents with a high burden of hemoglobinopathy: a diagnostic test accuracy study. *Am J Hematol* **92**, 196–203.

243. Yadav DK, Sharma B, Shrestha N, et al. (2018) Prevalence of risk factors of major non-communicable diseases among adolescents of higher secondary schools of Kaski district. *J* **16**, 307–312.

244. Yang L, Bovet P, Ma C, et al. (2019) Prevalence of underweight and overweight among young adolescents aged 12-15 years in 58 low-income and middle-income countries. *Pediatr Obes* **14**, e12468.

245. Yang WC, Fu CM, Su BW, et al. (2020) Child growth curves in high-altitude Ladakh: results from a cohort study. *Int J Environ Res Public Health* **17**, 22.

246. Yaya S & Ghose B (2020) Change in nutritional status among women of childbearing age in India (1998-2016). *Obesity Science and Practice* **6**, 535–543.

247. Young MF, Nguyen P, Tran LM, et al. (2020) A double edged sword? Improvements in economic conditions over a decade in India led to declines in undernutrition as well as increases in overweight among adolescents and women. *J Nutr* **150**, 364–372.

248. Zainab S & Kadir M (2016) Nutritional status and physical abuse among the children involved in domestic labour in Karachi Pakistan: a cross-sectional survey. *JPMA J Pak Med Assoc* **66**, 1243–1248.

**West and Central Africa**

1. Aaron GJ, Kariger P, Aliyu R, et al. (2011) A multi-micronutrient beverage enhances the vitamin A and zinc status of Nigerian primary schoolchildren. *The Journal of nutrition* **141**, 1565–1572.

2. Abdulkarim AA, Otuneye AT, Ahmed P, et al. (2016) Factors associated with adolescent malnutrition among Nigerian students. *Bangladesh Journal of Medical Science* **15**, 243–248.

3. Abizari AR & Ali Z (2019) Dietary patterns and associated factors of schooling Ghanaian adolescents. *J Health Popul Nutr* **38**, 2019/02/08 ed., 5.

4. Abizari AR, Azupogo F, Nagasu M, et al. (2017) Seasonality affects dietary diversity of school-age children in northern Ghana. *PLoS One* **12**, 2017/08/15 ed., e0183206.

5. Abizari AR, Buxton C, Kwara L, et al. (2014) School feeding contributes to micronutrient adequacy of Ghanaian schoolchildren. *Br J Nutr* **112**, 2014/07/06 ed., 1019–33.

6. Abizari AR, Dold S, Kupka R, et al. (2017) More than two-thirds of dietary iodine in children in northern Ghana is obtained from bouillon cubes containing iodized salt. *Public Health Nutr* **20**, 2016/12/03 ed., 1107–1113.

7. Abizari AR, Moretti D, Zimmermann MB, et al. (2012) Whole cowpea meal fortified with NaFeEDTA reduces iron deficiency among Ghanaian school children in a malaria endemic area. *J Nutr* **142**, 2012/08/24 ed., 1836–42.

8. Adebimpe WO (2019) Prevalence and knowledge of risk factors of childhood obesity among school-going children in Osogbo, south-western Nigeria. *Malawi Med J* **31**, 2019/05/31 ed., 19–24.

9. Adedeji IA, Bashir MF, Shwe DD, et al. (2018) Prevalence and correlates of stunting among the school-age population in North-Central Nigeria. *Pan Afr Med J* **31**, 2019/05/16 ed., 170.

10. Adeniyi AA, Oyapero OA, Ekekezie OO, et al. (2016) Dental caries and nutritional status of school children in Lagos, Nigeria - a preliminary survey. *J West Afr Coll Surg* **6**, 2017/09/01 ed., 15–38.

11. Adeniyi OF, Fagbenro GT & Olatona FA (2019) Overweight and obesity among school-aged children and maternal preventive practices against childhood obesity in select local government areas of Lagos, southwest, Nigeria. *Int J MCH AIDS* **8**, 2019/07/20 ed., 70–83.

12. Adenuga WU, Obembe TA, Odebunmi KO, et al. (2017) Prevalence and determinants of stunting among primary school children in rural and urban communities in Obafemi Owode local government area, southwestern Nigeria. *Ann Ib Postgrad Med* **15**, 2017/10/04 ed., 7–15.

13. Adeomi AA, Adelusi IO, Adedeji PO, et al. (2019) Nutritional status and cardiometabolic health among adolescents; findings from southwestern Nigeria. *BMC Nutr* **5**, 2020/03/11 ed., 45.

14. Adesina AF, Peterside O, Anochie I, et al. (2012) Weight status of adolescents in secondary schools in Port Harcourt using Body Mass Index (BMI). *Ital J Pediatr* **38**, 2012/07/25 ed., 31.

15. Adetunji AE, Adeniran KA, Olomu SC, et al. (2019) Socio-demographic factors associated with overweight and obesity among primary school children in semi-urban areas of mid-western Nigeria. *PLoS One* **14**, 2019/04/04 ed., e0214570.

16. Adeyemo FO & Chukwurah JN (2012) Parental socio-economic status as a correlate of pupils’ nutrition in public and private primary schools in Ogbomoso, Oyo state. *Journal of Medicine and Biomedical Research* **11**, 80–89.

17. Adom T, De Villiers A, Puoane T, et al. (2019) Prevalence and correlates of overweight and obesity among school children in an urban district in Ghana. *BMC Obes* **6**, 2019/04/16 ed., 14.

18. Adom T, Kengne AP, De Villiers A, et al. (2019) Association between school-level attributes and weight status of Ghanaian primary school children. *BMC Public Health* **19**, 2019/05/17 ed., 577.

19. Adom T, Kengne AP, De Villiers A, et al. (2019) Diagnostic accuracy of body mass index in defining childhood obesity: analysis of cross-sectional data from Ghanaian children. *Int J Environ Res Public Health* **17**, 2019/12/22 ed.

20. Afrifa-Anane E, Agyemang C, Codjoe SN, et al. (2015) The association of physical activity, body mass index and the blood pressure levels among urban poor youth in Accra, Ghana. *BMC Public Health* **15**, 2015/04/17 ed., 269.

21. Agbozo F, Atitto P, Jahn A, et al. (2018) Nutrient composition and dietary diversity of on-site lunch meals, and anthropometry of beneficiary children in private and public primary schools in Ghana. *Nutr Health* **24**, 2018/08/11 ed., 241–249.

22. Akinbodewa AA, Adejumo AO, Lamidi OA, et al. (2020) Clustering of cardiometabolic risk factors among children and adolescents in a rural community in Ondo, southwest Nigeria. *J Trop Pediatr* **66**, 2019/10/31 ed., 366–376.

23. Akinola IJ, Jarrett OO, Oduwole AO, et al. (2016) Prevalence of overweight and obesity among secondary school adolescents in an urban area of Lagos, Nigeria. *African Journal of Diabetes Medicine* **24**, 1–3.

24. Ali Z & Abizari AR (2018) Ramadan fasting alters food patterns, dietary diversity and body weight among Ghanaian adolescents. *Nutr J* **17**, 2018/08/14 ed., 75.

25. Alicke M, Boakye-Appiah JK, Abdul-Jalil I, et al. (2017) Adolescent health in rural Ghana: a cross-sectional study on the co-occurrence of infectious diseases, malnutrition and cardio-metabolic risk factors. *PLoS One* **12**, 2017/07/21 ed., e0180436.

26. Amenyah SD & Michels N (2016) Body size ideals, beliefs and dissatisfaction in Ghanaian adolescents: sociodemographic determinants and intercorrelations. *Public Health* **139**, 2016/06/28 ed., 112–120.

27. Amidu N, Owiredu WKBA, Saaka M, et al. (2013) Determinants of childhood obesity among basic school children aged 6-12 years in Tamale metropolis. *Journal of Medical and Biomedical Sciences* **2**, 26–34.

28. Anetor GO, Ogundele BO & Oyewole OE (2012) Effect of nutrition education on the eating habits of undergraduates in south-west, Nigeria. *Asian Journal of Epidemiology* **5**, 32–41.

29. Annan RA, Apprey C, Asamoah-Boakye O, et al. (2019) The relationship between dietary micronutrients intake and cognition test performance among school-aged children in government-owned primary schools in Kumasi metropolis, Ghana. *Food Sci Nutr* **7**, 2019/10/02 ed., 3042–3051.

30. Annan RA, Sowah SA, Apprey C, et al. (2020) Relationship between breakfast consumption, BMI status and physical fitness of Ghanaian school-aged children. *BMC Nutr* **6**, 2020/04/09 ed., 19.

31. Antwi J, Ohemeng A, Boateng L, et al. (2020) Primary school-based nutrition education intervention on nutrition knowledge, attitude and practices among school-age children in Ghana. *Glob Health Promot*, 2020/08/14 ed., 1757975920945241.

32. Arsenault JE, Moursi M, Olney DK, et al. (2020) Validation of 24-h dietary recall for estimating nutrient intakes and adequacy in adolescents in Burkina Faso. *Matern Child Nutr* **16**, 2020/04/28 ed., e13014.

33. Aryeetey R, Lartey A, Marquis GS, et al. (2017) Prevalence and predictors of overweight and obesity among school-aged children in urban Ghana. *BMC Obes* **4**, 2017/12/08 ed., 38.

34. Asiegbu UV, Asiegbu OG, Onyire BN, et al. (2017) Assessment of gross malnutrition among primary school children using body mass index as an assessment tool in abakaliki metropolis of Ebonyi State, South-East Nigeria. *Niger J Clin Pract* **20**, 2017/06/29 ed., 693–699.

35. Atto V, Bleyere MN, Konan AB, et al. (2013) Depletion of iron stores and main associated parameters in adolescents of Cote d’ivoire. *Pakistan Journal of Nutrition* **12**, 188–196.

36. Ayeh-Kumi PF, Addo-Osafo K, Attah SK, et al. (2016) Malaria, helminths and malnutrition: a cross-sectional survey of school children in the South-Tongu district of Ghana. *BMC Res Notes* **9**, 2016/04/28 ed., 242.

37. Ayogu R (2019) Energy and nutrient intakes of rural Nigerian schoolchildren: relationship with dietary diversity. *Food Nutr Bull* **40**, 2019/05/09 ed., 241–253.

38. Ayogu RNB, Afiaenyi IC, Madukwe EU, et al. (2018) Prevalence and predictors of under-nutrition among school children in a rural South-eastern Nigerian community: a cross sectional study. *BMC Public Health* **18**, 2018/05/04 ed., 587.

39. Ayogu RN, Nnam NM, Ibemesi O, et al. (2016) Prevalence and factors associated with anthropometric failure, vitamin A and iron deficiency among adolescents in a Nigerian urban community. *Afr Health Sci* **16**, 2016/09/09 ed., 389–98.

40. Ayogu RN, Okafor AM & Ene-Obong HN (2015) Iron status of schoolchildren (6-15 years) and associated factors in rural Nigeria. *Food Nutr Res* **59**, 2015/05/09 ed., 26223.

41. Ayogu RNB & Onah TP (2018) Impact of cowpea fortified cookies on anthropometric and micronutrient status of primary school children: a randomized, single-blind controlled trial. *Niger J Clin Pract* **21**, 2018/10/10 ed., 1341–1348.

42. Azupogo F, Abizari AR, Aurino E, et al. (2020) Malnutrition, hypertension risk, and correlates: an analysis of the 2014 Ghana Demographic and Health Survey data for 15-19 years adolescent boys and girls. *Nutrients* **12**, 2020/09/12 ed.

43. Azupogo F, Aurino E, Gelli A, et al. (2019) Agro-ecological zone and farm diversity are factors associated with haemoglobin and anaemia among rural school-aged children and adolescents in Ghana. *Matern Child Nutr* **15**, 2018/07/27 ed., e12643.

44. Ba DM, Ssentongo P, Liao D, et al. (2020) Non-iodized salt consumption among women of reproductive age in sub-Saharan Africa: a population-based study. *Public health nutrition* **23**, 2759–2769.

45. Bamidele JO, Abodunrin OL, Olajide FO, et al. (2010) Prevalence and determinants of anemia among primary school pupils of a peri-urban community in Osun State, Nigeria. *Int J Adolesc Med Health* **22**, 2011/03/17 ed., 461–8.

46. Bamidele JO, Olarinmoye EOA, Olajide FO, et al. (2011) Prevalence and socio-demographic determinants of under-weight and pre-obesity among in-school adolescents in olorunda local government area, Osun State, Nigeria. *TAF Preventive Medicine Bulletin* **10**, 397–402.

47. Bationo JF, Zeba AN, Abbeddou S, et al. (2018) Serum carotenoids reveal poor fruit and vegetable intake among schoolchildren in Burkina Faso. *Nutrients* **10**, 2018/10/06 ed.

48. Bationo JF, Zeba AN, Coulibaly ND, et al. (2019) Liver retinol estimated by 13C-retinol isotope dilution at 7 versus 14 days in Burkinabe schoolchildren. *Experimental Biology and Medicine* **244**, 1430–1437.

49. Berhane Y, Canavan CR, Darling AM, et al. (2020) The age of opportunity: prevalence of key risk factors among adolescents 10-19 years of age in nine communities in sub-Saharan Africa. *Trop Med Int Health* **25**, 2019/11/08 ed., 15–32.

50. Bleyere MN, Kokore BA, Konan AB, et al. (2013) Prevalence of child malnutrition through their anthropometric indices in school canteens of Abidjan (Cote d’ivoire). *Pakistan Journal of Nutrition* **12**, 60–70.

51. Brabin L, Roberts SA, Tinto H, et al. (2020) Iron status of Burkinabé adolescent girls predicts malaria risk in the following rainy season. *Nutrients* **12**, 2020/05/21 ed.

52. Buhendwa RA, Roelants M, Thomis M, et al. (2017) Nutritional status and height, weight and BMI centiles of school-aged children and adolescents of 6-18-years from Kinshasa (DRC). *Ann Hum Biol* **44**, 2017/05/26 ed., 554–561.

53. Carvalho AC, Machado A, Embalo AR, et al. (2018) Endemic goiter and iodine deficiency status among Guinea-Bissau school-age children. *Eur J Clin Nutr* **72**, 2017/12/30 ed., 1576–1582.

54. Chelo D, Mah EM, Chiabi EN, et al. (2019) Prevalence and factors associated with hypertension in primary school children, in the centre region of Cameroon. *Transl Pediatr* **8**, 2020/01/30 ed., 391–397.

55. Chimhashu T, Malan L, Baumgartner J, et al. (2018) Sensitivity of fatty acid desaturation and elongation to plasma zinc concentration: a randomised controlled trial in Beninese children. *Br J Nutr* **119**, 2018/01/22 ed., 610–619.

56. Chinedu SN, Eboji OK & Emiloju OC (2012) Trends in weight abnormality of school children and adolescents in Nigeria. *Journal of Medical Sciences (Faisalabad)* **12**, 239–243.

57. Chinedu SN, Emiloju OC, Azuh DE, et al. (2017) Association between age, gender and body weight in educational institutions in Ota, Southwest Nigeria. *Asian Journal of Epidemiology* **10**, 144–149.

58. Choukem SP, Kamdeu-Chedeu J, Leary SD, et al. (2017) Overweight and obesity in children aged 3-13 years in urban Cameroon: a cross-sectional study of prevalence and association with socio-economic status. *BMC Obes* **4**, 2017/02/07 ed., 7.

59. Chukwunonso Ejike ECC, Chidi Ugwu E & Lawrence Ezeanyika US (2010) Physical growth and nutritional status of a cohort of semi-urban Nigerian adolescents. *Pakistan Journal of Nutrition* **9**, 392–397.

60. Clarke SE, Rouhani S, Diarra S, et al. (2017) Impact of a malaria intervention package in schools on Plasmodium infection, anaemia and cognitive function in schoolchildren in Mali: a pragmatic cluster-randomised trial. *BMJ Glob Health* **2**, 2017/10/31 ed., e000182.

61. Daboné C, Delisle HF & Receveur O (2011) Poor nutritional status of schoolchildren in urban and peri-urban areas of Ouagadougou (Burkina Faso). *Nutr J* **10**, 2011/04/21 ed., 34.

62. Dapare PPM, Adams Y, Djabuni EK, et al. (2017) Nutrient intake, physical activity and nutritional status among second cycle students in Tamale, Ghana. *Journal of Medical and Biomedical Sciences* **6**, 25–37.

63. Dapi LN, Hörnell A, Janlert U, et al. (2011) Energy and nutrient intakes in relation to sex and socio-economic status among school adolescents in urban Cameroon, Africa. *Public Health Nutr* **14**, 2010/12/07 ed., 904–13.

64. Darling AM, Sunguya B, Ismail A, et al. (2020) Gender differences in nutritional status, diet and physical activity among adolescents in eight countries in sub-Saharan Africa. *Trop Med Int Health* **25**, 2019/11/07 ed., 33–43.

65. Diouf A, Adom T, Aouidet A, et al. (2018) Body mass index vs deuterium dilution method for establishing childhood obesity prevalence, Ghana, Kenya, Mauritius, Morocco, Namibia, Senegal, Tunisia and United Republic of Tanzania. *Bulletin of the World Health Organization* **96**, 772–781.

66. Diouf A, Diongue O, Nde M, et al. (2018) Validity of bioelectrical impedance analysis in predicting total body water and adiposity among Senegalese school-aged children. *PLoS One* **13**, 2018/10/12 ed., e0204486.

67. Djadou KE, Sadzo-Hetsu K, Koffi KS, et al. (2010) Prevalence of obesity in urban scolar area (Togo). *Journal de Pediatrie et de Puericulture* **23**, 335–339.

68. Doku D, Koivusilta L, Raisamo S, et al. (2013) Socio-economic differences in adolescents’ breakfast eating, fruit and vegetable consumption and physical activity in Ghana. *Public Health Nutr* **16**, 2011/10/28 ed., 864–72.

69. Egbi G, Gbogbo S, Mensah GE, et al. (2018) Effect of green leafy vegetables powder on anaemia and vitamin-A status of Ghanaian school children. *BMC Nutr* **4**, 2018/06/08 ed., 27.

70. Egbi G, Glover-Amengor M, Tohouenou MM, et al. (2020) Contribution of Amaranthus cruentus and Solanum macrocarpon leaves flour to nutrient intake and effect on nutritional status of rural school children in Volta Region, Ghana. *J Nutr Metab* **2020**, 2020/06/23 ed., 1015280.

71. Egbi G, Steiner-Asiedu M, Kwesi FS, et al. (2014) Anaemia among school children older than five years in the Volta Region of Ghana. *Pan Afr Med J* **17 Suppl 1**, 2014/03/20 ed., 10.

72. Elkhouri Edde C, Delisle H, Dabone C, et al. (2020) Impact of the Nutrition-Friendly School Initiative: analysis of anthropometric and biochemical data among school-aged children in Ouagadougou. *Glob Health Promot* **27**, 2019/01/17 ed., 26–34.

73. Elusiyan JB, Ibekwe MU, Alkali YS, et al. (2016) Growth characteristics of contemporary school-age Nigerian children. *J Trop Pediatr* **62**, 2016/03/19 ed., 345–51.

74. Eme PE, Onuoha NO & Mbah OB (2016) Fat-related anthropometric variables and regional patterns of body size and adiposity of adolescents in Aba South LGA, Abia State, Nigeria. *Food Nutr Bull* **37**, 2016/05/06 ed., 401–408.

75. Ene-Obong H, Ibeanu V, Onuoha N, et al. (2012) Prevalence of overweight, obesity, and thinness among urban school-aged children and adolescents in southern Nigeria. *Food Nutr Bull* **33**, 2013/02/22 ed., 242–50.

76. Erismann S, Knoblauch AM, Diagbouga S, et al. (2017) Prevalence and risk factors of undernutrition among schoolchildren in the Plateau Central and Centre-Ouest regions of Burkina Faso. *Infect Dis Poverty* **6**, 2017/01/20 ed., 17.

77. Essien E, Haruna MJ & Emebu PK (2012) Prevalence of malnutrition and its effects on the academic performance of students in some selected secondary schools in Sokoto metropolis. *Pakistan Journal of Nutrition* **11**, 511–515.

78. Eze JN, Oguonu T, Ojinnaka NC, et al. (2017) Physical growth and nutritional status assessment of school children in Enugu, Nigeria. *Niger J Clin Pract* **20**, 2016/12/14 ed., 64–70.

79. Ezezika O, Oh J, Edeagu N, et al. (2018) Gamification of nutrition: a preliminary study on the impact of gamification on nutrition knowledge, attitude, and behaviour of adolescents in Nigeria. *Nutr Health* **24**, 2018/07/06 ed., 137–144.

80. Fall CH, Abera M, Chopra H, et al. (2020) Anthropometric nutritional status, and social and dietary characteristics of African and Indian adolescents taking part in the TALENT (Transforming Adolescent Lives through Nutrition) qualitative study. *Public Health Nutr*, 2020/08/06 ed., 1–12.

81. Faye J, Diop M, Gati Ouonkoye R, et al. (2011) Prevalence of child and teenage obesity in schools in Dakar. *Bull Soc Pathol Exot* **104**, 2010/12/22 ed., 49–52.

82. Fernandes M, Folson G, Aurino E, et al. (2017) A free lunch or a walk back home? The school food environment and dietary behaviours among children and adolescents in Ghana. *Food Secur* **9**, 2017/01/01 ed., 1073–1090.

83. Fetuga MB, Ogunlesi TA, Adekanmbi AF, et al. (2011) Growth pattern of schoolchildren in Sagamu, Nigeria using the CDC standards and 2007 WHO standards. *Indian Pediatr* **48**, 2010/12/21 ed., 523–8.

84. Fetuga MB, Ogunlesi TA, Adekanmbi AF, et al. (2011) Nutritional status of semi-urban Nigerian school children using the 2007 WHO reference population. *West Afr J Med* **30**, 2012/07/04 ed., 331–6.

85. Fiorentino M, Bastard G, Sembène M, et al. (2013) Anthropometric and micronutrient status of school-children in an urban West Africa setting: a cross-sectional study in Dakar (Senegal). *PLoS One* **8**, 2014/01/07 ed., e84328.

86. Fiorentino M, Landais E, Bastard G, et al. (2016) Nutrient intake is insufficient among senegalese urban school children and adolescents: results from two 24 h recalls in state primary schools in Dakar. *Nutrients* **8**, 2016/10/25 ed.

87. Francis A, Seline O & Angela O (2010) Nutritional status of newly enrolled primary school children in Jos-Plateau, Nigeria. *Pakistan Journal of Nutrition* **9**, 1166–1170.

88. Galetti V, Kujinga P, Mitchikpè CE, et al. (2015) Efficacy of highly bioavailable zinc from fortified water: a randomized controlled trial in rural Beninese children. *Am J Clin Nutr* **102**, 2015/10/16 ed., 1238–48.

89. Galetti V, Mitchikpè CE, Kujinga P, et al. (2016) Rural Beninese children are at risk of zinc deficiency according to stunting prevalence and plasma zinc concentration but not dietary zinc intakes. *J Nutr* **146**, 2015/11/27 ed., 114–23.

90. Ganle JK, Boakye PP & Baatiema L (2019) Childhood obesity in urban Ghana: evidence from a cross-sectional survey of in-school children aged 5-16 years. *BMC Public Health* **19**, 2019/11/28 ed., 1561.

91. Garbaand CMG & Mbofung CMF (2010) Relationship between malnutrition and parasitic infection among school children in the adamawa region of cameroon. *Pakistan Journal of Nutrition* **9**, 1094–1099.

92. Gelli A, Aurino E, Folson G, et al. (2019) A school meals program implemented at scale in Ghana increases height-for-age during midchildhood in girls and in children from poor households: a cluster randomized trial. *J Nutr* **149**, 2019/05/18 ed., 1434–1442.

93. Glozah FN & Pevalin DJ (2015) Perceived social support and parental education as determinants of adolescents’ physical activity and eating behaviour: a cross-sectional survey. *Int J Adolesc Med Health* **27**, 2014/08/26 ed., 253–9.

94. Goon DT, Toriola AL, Shaw BS, et al. (2011) Anthropometrically determined nutritional status of urban primary schoolchildren in Makurdi, Nigeria. *BMC Public Health* **11**, 2011/10/07 ed., 769.

95. Gosdin L, Tripp K, Mahama AB, et al. (2020) Predictors of anaemia among adolescent schoolchildren of Ghana. *J Nutr Sci* **9**, 2020/09/29 ed., e43.

96. Gyamfi D, Obirikorang C, Acheampong E, et al. (2019) Weight management among school-aged children and adolescents: a quantitative assessment in a Ghanaian municipality. *BMC Pediatr* **19**, 2019/10/28 ed., 376.

97. Gyamfi D, Wiafe YA, Ofori Awuah E, et al. (2020) Goitre prevalence and urinary iodine concentration in school-aged children in the Ashanti Region of Ghana. *Int J Endocrinol* **2020**, 2020/04/11 ed., 3759786.

98. Hassan A, Onabanjo OO & Oguntona CRB (2012) Nutritional assessment of school-age children attending conventional primary and integrated Qur’anic Schools in Kaduna. *Research Journal of Medical Sciences* **6**, 187–192.

99. Henry-Unaeze HN & Okonkwo CN (2011) Food consumption pattern and calcium status of adolescents in Nnewi, Nigeria. *Pakistan Journal of Nutrition* **10**, 317–321.

100. Hess SY, Ouédraogo CT, Young RR, et al. (2017) Urinary iodine concentration identifies pregnant women as iodine deficient yet school-aged children as iodine sufficient in rural Niger. *Public Health Nutr* **20**, 2016/12/16 ed., 1154–1161.

101. Igbokwe O, Adimorah G, Ikefuna A, et al. (2017) Socio-demographic determinants of malnutrition among primary school aged children in Enugu, Nigeria. *Pan Afr Med J* **28**, 2018/06/27 ed., 248.

102. Intiful FD & Lartey A (2014) Breakfast habits among school children in selected communities in the eastern region of Ghana. *Ghana Med J* **48**, 2015/02/11 ed., 71–7.

103. Izuora AN, Animasahun BA, Nwodo U, et al. (2013) Assessment of overweight and obesity among Nigerian children and adolescents using triceps skin-fold thickness and body mass index. *Clin Obes* **3**, 2013/06/01 ed., 103–11.

104. Janha RE, Hardy-Johnson P, Kehoe SH, et al. (2020) Exploring influences on adolescent diet and physical activity in rural Gambia, West Africa: food insecurity, culture and the natural environment. *Public Health Nutr*, 2020/08/29 ed., 1–11.

105. Jesson J, Kouakou EK, Hardy-Johnson P, et al. (2020) Adolescent nutrition and physical activity in low-income suburbs of Abidjan, Côte d’lvoire: the gap between knowledge, aspirations and possibilities. *Public Health Nutr*, 2020/07/24 ed., 1–11.

106. Jiwani SS, Gatica-Domínguez G, Crochemore-Silva I, et al. (2020) Trends and inequalities in the nutritional status of adolescent girls and adult women in sub-Saharan Africa since 2000: a cross-sectional series study. *BMJ global health* **5**, e002948.

107. Juwara A, Huang N, Chien LY, et al. (2016) Stunting and weight statuses of adolescents differ between public and private schools in urban Gambia. *Int J Public Health* **61**, 2016/05/26 ed., 717–726.

108. Kabongo MM, Linsuke S, Baloji S, et al. (2018) Schistosoma manson infection and its association with nutrition and health outcomes: A household survey in school-aged children living in Kasansa, Democratic Republic of the Congo. *Pan African Medical Journal* **31**, 197.

109. Kouéta F, Dao L, Dao F, et al. (2011) Factors associated with overweight and obesity in children in Ouagadougou (Burkina Faso). *Sante* **21**, 2012/03/13 ed., 227–31.

110. Kramoh KE, N’Goran Y N, Aké-Traboulsi E, et al. (2012) Prevalence of obesity in school children in Ivory Coast. *Ann Cardiol Angeiol (Paris)* **61**, 2012/06/09 ed., 145–9.

111. Kwabla MP, Gyan C & Zotor F (2018) Nutritional status of in-school children and its associated factors in Denkyembour District, eastern region, Ghana: comparing schools with feeding and non-school feeding policies. *Nutr J* **17**, 2018/01/14 ed., 8.

112. Larbi IA, Klipstein-Grobusch K, Amoah AS, et al. (2011) High body mass index is not associated with atopy in schoolchildren living in rural and urban areas of Ghana. *BMC Public Health* **11**, 2011/06/15 ed., 469.

113. Lateef OJ, Njogu E, Kiplamai F, et al. (2016) Breakfast, food consumption pattern and nutritional status of students in public secondary schools in Kwara state, Nigeria. *Pakistan Journal of Nutrition* **15**, 140–147.

114. Lion R, Arulogun O, Titiloye M, et al. (2018) The effect of the ‘Follow in my Green Food Steps’ programme on cooking behaviours for improved iron intake: a quasi-experimental randomized community study. *Int J Behav Nutr Phys Act* **15**, 2018/08/18 ed., 79.

115. M.M MO, Afrifa D, Asamoah MA, et al. (2020) ‘AMPE’ exercise programme has positive effects on anthropometric and physiological parameters of school children: a pilot study. *Ethiopian journal of health sciences* **30**, 143–146.

116. Madukwe EU, Ani PN & Maduabuchi M (2013) Iodine content of household salt and urinary iodine of primary school pupils in commercial towns in Nsukka senatorial zone, Enugu state, Nigeria. *Pakistan Journal of Nutrition* **12**, 587–593.

117. Manyanga T, El-Sayed H, Doku DT, et al. (2014) The prevalence of underweight, overweight, obesity and associated risk factors among school-going adolescents in seven African countries. *BMC Public Health* **14**, 2014/08/30 ed., 887.

118. Maruf FA, Aronu U, Chukwuegbu K, et al. (2013) Influence of gender on prevalence of overweight and obesity in Nigerian schoolchildren and adolescents. *Tanzan J Health Res* **15**, 2013/10/01 ed., 247–51.

119. Matangila JR, Doua JY, Linsuke S, et al. (2014) Malaria, schistosomiasis and soil transmitted helminth burden and their correlation with anemia in children attending primary schools in Kinshasa, Democratic Republic of Congo. *PLoS One* **9**, 2014/11/06 ed., e110789.

120. Mbuh JV & Nembu NE (2013) Malnutrition and intestinal helminth infections in schoolchildren from Dibanda, Cameroon. *J Helminthol* **87**, 2012/01/26 ed., 46–51.

121. Michels N & Amenyah SD (2017) Body size ideals and dissatisfaction in Ghanaian adolescents: role of media, lifestyle and well-being. *Public Health* **146**, 2017/04/14 ed., 65–74.

122. Mogre V, Aneyire ES & Gyamfi EK (2013) Physical activity and BMI status of school-age children in Tamale, Northern Ghana. *Pakistan Journal of Nutrition* **12**, 484–490.

123. Mohammed H & Vuvor F (2012) Prevalence of childhood overweight/obesity in basic school in Accra. *Ghana Med J* **46**, 2013/05/11 ed., 124–7.

124. Musa DI, Toriola AL, Monyeki MA, et al. (2012) Prevalence of childhood and adolescent overweight and obesity in Benue State, Nigeria. *Trop Med Int Health* **17**, 2012/09/15 ed., 1369–75.

125. Mustapha RA & Sanusi RA (2013) Overweight and obesity among in-school adolescents in Ondo state, Southwest Nigeria. *African Journal of Biomedical Research* **16**, 205–210.

126. Musung JM, Muyumba EK, Nkulu DN, et al. (2019) Prevalence of overweight and obesity among adolescents in school in Lubumbashi, Democratic Republic of Congo. *Pan Afr Med J* **32**, 2019/05/31 ed., 49.

127. Nago ES, Lachat CK, Huybregts L, et al. (2010) Food, energy and macronutrient contribution of out-of-home foods in school-going adolescents in Cotonou, Benin. *Br J Nutr* **103**, 2009/10/13 ed., 281–8.

128. Nago ES, Verstraeten R, Lachat CK, et al. (2012) Food safety is a key determinant of fruit and vegetable consumption in urban Beninese adolescents. *J Nutr Educ Behav* **44**, 2011/11/25 ed., 548–55.

129. Navti LK, Ferrari U, Tange E, et al. (2014) Contribution of socioeconomic status, stature and birth weight to obesity in Sub-Saharan Africa: cross-sectional data from primary school-age children in Cameroon. *BMC Public Health* **14**, 2014/04/09 ed., 320.

130. Ndiaye P, Leye MM & Tal Dia A (2016) Overweight, obesity and associated factors among public secondary school students in Dakar. *Sante Publique* **28**, 2017/02/06 ed., 687–694.

131. Ndukwu CI, Egbuonu I, Ulasi TO, et al. (2013) Determinants of undernutrition among primary school children residing in slum areas of a Nigerian city. *Niger J Clin Pract* **16**, 2013/04/09 ed., 178–83.

132. Ng M, Fleming T, Robinson M, et al. (2014) Global, regional, and national prevalence of overweight and obesity in children and adults during 1980-2013: a systematic analysis for the Global Burden of Disease Study 2013. *Lancet* **384**, 2014/06/02 ed., 766–81.

133. Niba LL, Itor PB, Aurelie YKS, et al. (2019) A median analysis of factors influencing body fatness in urban school-age children in Cameroon. *J Nutr Metab* **2019**, 2019/03/14 ed., 1856069.

134. Nwamarah JU, Otitoju Olawale OGTO & Emewulu CUD (2015) Iodine and nutritional status of primary school children in a Nigerian community Okpuje, in Nsukka LGA, Enugu State, Nigeria. *Der Pharmacia Lettre* **7**, 271–280.

135. Nwanneka AP, Nkechi EOH & Orji UP (2016) Eating habits, activity pattern and socioeconomic factors that affect the prevalence of overweight and obesity among adolescents in urban areas of Enugu state, Nigeria. *Pakistan Journal of Nutrition* **15**, 288–294.

136. Nwizu SE, Njokanma OF, Okoromah CA, et al. (2011) Relationship between bioelectrical impedance analysis and body mass index in adolescent urban Nigerians. *West Afr J Med* **30**, 2011/10/11 ed., 99–103.

137. Nyarko R, Torpey K & Ankomah A (2018) Schistosoma haematobium, Plasmodium falciparum infection and anaemia in children in Accra, Ghana. *Trop Dis Travel Med Vaccines* **4**, 2018/05/04 ed., 3.

138. Nyawornota VK, Aryeetey R, Bosomprah S, et al. (2013) An exploratory study of physical activity and over-weight in two senior high schools in the Accra Metropolis. *Ghana Med J* **47**, 2014/03/29 ed., 197–203.

139. Obembe TA, Adenuga WU & Asuzu MC (2018) Determinants of wasting among schoolchildren in a Southwestern state of Nigeria: Implications to strengthen the nutritional component of primary health-care model. *J Family Med Prim Care* **7**, 2018/09/21 ed., 671–677.

140. Odenigbo UM, Nkwoala CC & Okpala OC (2010) Impact of birth weight on the nutritional status and academic performance of school age children. *Pakistan Journal of Nutrition* **9**, 1157–1161.

141. Odunaiya NA, Louw QA & Grimmer KA (2015) Are lifestyle cardiovascular disease risk factors associated with pre-hypertension in 15-18 years rural Nigerian youth? A cross sectional study. *BMC Cardiovasc Disord* **15**, 2015/11/06 ed., 144.

142. Oduwole AA, Ladapo TA, Fajolu IB, et al. (2012) Obesity and elevated blood pressure among adolescents in Lagos, Nigeria: a cross-sectional study. *BMC Public Health* **12**, 2012/08/08 ed., 616.

143. Ogum Alangea D, Aryeetey RN, Gray HL, et al. (2018) Dietary patterns and associated risk factors among school age children in urban Ghana. *BMC Nutr* **4**, 2018/05/10 ed., 22.

144. Ogunkunle MO & Oludele AS (2013) Food intake and meal pattern of adolescents in school in Ila Orangun, south-west Nigeria. *South African Journal of Clinical Nutrition* **26**, 188–193.

145. Ogunsile SE & Ogundele BO (2016) Effect of game-enhanced nutrition education on knowledge, attitude and practice of healthy eating among adolescents in Ibadan, Nigeria. *International Journal of Health Promotion and Education* **54**, 207–216.

146. Ohuche IO, Ayuk AC, Ubesie AC, et al. (2020) Soil-transmitted helminthiasis: a neglected tropical disease among urban slum dwelling school-aged children of a sub-Saharan African city. *Niger Postgrad Med J* **27**, 2020/07/21 ed., 184–189.

147. Ojofeitimi EO, Olugbenga-Bello AI, Adekanle DA, et al. (2011) Pattern and determinants of obesity among adolescent females in private and public schools in the Olorunda Local Government Area of Osun State, Nigeria: a comparative study. *J Public Health Afr* **2**, 2011/02/11 ed., e11.

148. Okafor AM, Odo EO & Onodigbo EO (2019) Dietary diversity: Association with academic performance and anthropometric indices of rural Nigerian school children. *Pakistan Journal of Nutrition* **19**, 61–67.

149. Okoh BA & Alikor EA (2015) Prevalence of overweight and obesity among primary school children in Port Harcourt, Nigeria. *Niger Postgrad Med J* **22**, 2015/04/16 ed., 9–14.

150. Okoro EO, Ogunbiyi AO, George AO, et al. (2016) Association of diet with acne vulgaris among adolescents in Ibadan, southwest Nigeria. *Int J Dermatol* **55**, 2016/01/11 ed., 982–8.

151. Olatona FA, Aderibigbe IO, Aderibigbe SA, et al. (2018) Energy drinks consumption among football players in Lagos, Nigeria. *South African Journal of Clinical Nutrition* **31**, 84–88.

152. Olopade BO, Idowu CO, Oyelese AO, et al. (2018) Intestinal parasites, nutritional status and cognitive function among primary school pupils in Ile-Ife, Osun State, Nigeria. *Afr J Infect Dis* **12**, 2018/08/16 ed., 21–28.

153. Oludare OI (2015) Pattern of BMI percentiles among adolescents in a semi-urban community in South-Western Nigeria. *Pakistan Journal of Nutrition* **14**, 570–574.

154. Olumakaiye MF, Atinmo T & Olubayo-Fatiregun MA (2010) Food consumption patterns of Nigerian adolescents and effect on body weight. *J Nutr Educ Behav* **42**, 2010/01/20 ed., 144–51.

155. Olusanya JO & Omotayo OA (2011) Prevalence of obesity among undergraduate students of Tai Solarin University of Education, Ijagun, Ijebu-Ode. *Pakistan Journal of Nutrition* **10**, 940–946.

156. Omigbodun OO, Adediran KI, Akinyemi JO, et al. (2010) Gender and rural-urban differences in the nutritional status of in-school adolescents in south-western Nigeria. *J Biosoc Sci* **42**, 2010/06/10 ed., 653–76.

157. Omisore B, Omisore A & Abioye-Kuteyi E (2015) Original research: obesity prevalence and metabolic differences between obese and non-obese school adolescents in south-western Nigeria. *South African Family Practice* **57**.

158. Omobuwa O, Alebiosu CO, Olajide FO, et al. (2014) Assessment of nutritional status of in-school adolescents in Ibadan, Nigeria. *South African Family Practice* **56**, 246–250.

159. Omuemu VO & Oko-Oboh AG (2015) Meal pattern and soft drink consumption among in-school adolescents in Benin-city, Edo state, nigeria. *Journal of Medicine and Biomedical Research* **14**, 72–81.

160. Omuemu VO & Omuemu CE (2010) The prevalence of overweight and its risk factors among adolescents in an urban city in Edo State. *Niger J Clin Pract* **13**, 2010/05/27 ed., 128–33.

161. Onoja US, Iloeje MC, Onoja NC, et al. (2019) Nutritional status of adolescent schoolchildren in south east Nigeria. *Pakistan Journal of Nutrition* **18**, 845–851.

162. Onyiriuka AN, Umoru DD & Ibeawuchi AN (2013) Weight status and eating habits of adolescent Nigerian urban secondary school girls. *SAJCH South African Journal of Child Health* **7**, 108–112.

163. Opara DC, Ikpeme EE & Ekanem US (2010) Prevalence of stunting, underweight and obesity in school aged children in Uyo, Nigeria. *Pakistan Journal of Nutrition* **9**, 459–466.

164. Ouédraogo O, Garanet F, Compaoré E, et al. (2019) Prevalence and factors associated with stunting among schoolchildren in Dori in the Sahel region of Burkina Faso. *Sante Publique* **Vol. 31**, 2019/06/19 ed., 153–163.

165. Oumar Bâ H, Menta I, Camara Y, et al. (2014) Overweight and obesity in the general population of 5-19 years in urban Bamako (Mali). *Pan Afr Med J* **19**, 2014/01/01 ed., 351.

166. Oyesiji OV, Otemuyiwa IO, Falade OS, et al. (2019) Changing trend in nutrition and food consumption predisposes school children to overweight and obesity in Ile-Ife, Osun State, Nigeria. *Nutr Health* **25**, 2019/01/19 ed., 93–101.

167. Prentice A, Dibba B, Sawo Y, et al. (2012) The effect of prepubertal calcium carbonate supplementation on the age of peak height velocity in Gambian adolescents. *Am J Clin Nutr* **96**, 2012/09/20 ed., 1042–50.

168. Sabageh AO, Ogunfowokan AA & Ojofeitimi EO (2013) Obesity and body image discrepancy among school adolescents in Ile-Ife, Osun State, Nigeria. *Pakistan Journal of Nutrition* **12**, 377–381.

169. Sadoh WE, Israel-Aina YT, Sadoh AE, et al. (2017) Comparison of obesity, overweight and elevated blood pressure in children attending public and private primary schools in Benin City, Nigeria. *Niger J Clin Pract* **20**, 2017/08/10 ed., 839–846.

170. Sadoh WE, Sadoh AE & Onyiriuka AN (2016) Physical activity, body mass index and blood pressure in primary school pupils attending private schools. *Afr Health Sci* **16**, 2017/05/10 ed., 947–953.

171. Sagbo H, Ekouevi DK, Ranjandriarison DT, et al. (2018) Prevalence and factors associated with overweight and obesity among children from primary schools in urban areas of Lomé, Togo. *Public Health Nutr* **21**, 2018/01/25 ed., 1048–1056.

172. Sanou AS, Diallo AH, Holding P, et al. (2018) Association between stunting and neuro-psychological outcomes among children in Burkina Faso, West Africa. *Child and Adolescent Psychiatry and Mental Health* **12**, 30.

173. Schlossman N (2018) Higher levels of dairy result in improved physical outcomes: a synthesis of 3 randomized controlled trials in Guinea-Bissau comparing supplements with different levels of dairy ingredients among children 6 to 59 months, 5 to 19 year olds, and mothers in preschools, primary schools, and villages, and the implications for programs. *Food Nutr Bull* **39**, 2018/09/22 ed., S35-s44.

174. Schoenbuchner SM, Moore SE, Johnson W, et al. (2018) In rural Gambia, do adolescents have increased nutritional vulnerability compared with adults? *Annals of the New York Academy of Sciences* **1416**, 77–85.

175. Senbanjo IO & Oshikoya KA (2010) Physical activity and body mass index of school children and adolescents in Abeokuta, Southwest Nigeria. *World J Pediatr* **6**, 2010/06/16 ed., 217–22.

176. Senbanjo IO & Oshikoya KA (2012) Obesity and blood pressure levels of adolescents in Abeokuta, Nigeria. *Cardiovasc J Afr* **23**, 2011/11/01 ed., 260–4.

177. Senbanjo IO, Oshikoya KA & Njokanma OF (2011) Changes in the nutritional status of school children and adolescents in Abeokuta, Nigeria between 1983 and 2006. *West Afr J Med* **30**, 2012/07/13 ed., 425–31.

178. Senbanjo IO, Oshikoya KA & Njokanma OF (2014) Upper arm composition and nutritional status of school children and adolescents in Abeokuta, Southwest Nigeria. *World J Pediatr* **10**, 2014/03/07 ed., 336–42.

179. Senbanjo IO, Oshikoya KA, Odusanya OO, et al. (2011) Prevalence of and risk factors for stunting among school children and adolescents in Abeokuta, southwest Nigeria. *J Health Popul Nutr* **29**, 2011/10/01 ed., 364–70.

180. Sufiyan M, Sabitu K & Mande A (2011) Evaluation of the effectiveness of deworming and participatory hygiene education strategy in controlling anemia among children aged 6-15 years in Gadagau community, Giwa LGA, Kaduna, Nigeria. *Annals of African Medicine* **10**, 6–12.

181. Sumbele IU, Bopda OS, Kimbi HK, et al. (2015) Nutritional status of children in a malaria meso endemic area: cross sectional study on prevalence, intensity, predictors, influence on malaria parasitaemia and anaemia severity. *BMC Public Health* **15**, 2015/11/07 ed., 1099.

182. Sumbele IU, Kimbi HK, Ndamukong-Nyanga JL, et al. (2015) Malarial anaemia and anaemia severity in apparently healthy primary school children in urban and rural settings in the Mount Cameroon area: cross sectional survey. *PLoS One* **10**, 2015/04/22 ed., e0123549.

183. Sumbele IUN, Nkain AJ, Ning TR, et al. (2020) Influence of malaria, soil-transmitted helminths and malnutrition on haemoglobin level among school-aged children in Muyuka, Southwest Cameroon: a cross-sectional study on outcomes. *PLoS One* **15**, 2020/04/01 ed., e0230882.

184. Tabi ESB, Cumber SN, Juma KO, et al. (2019) A cross-sectional survey on the prevalence of anaemia and malnutrition in primary school children in the Tiko Health District, Cameroon. *Pan Afr Med J* **32**, 2019/06/22 ed., 111.

185. Teh RN, Sumbele IUN, Meduke DN, et al. (2018) Malaria parasitaemia, anaemia and malnutrition in children less than 15 years residing in different altitudes along the slope of Mount Cameroon: prevalence, intensity and risk factors. *Malar J* **17**, 2018/09/27 ed., 336.

186. Ubesie A, Okoli C, Uwaezuoke S, et al. (2016) Affluence and adolescent obesity in a city in south-east Nigerian: A cross-sectional survey. *Annals of Tropical Medicine and Public Health* **9**, 251–254.

187. Udo N, Bassey IE, Akpan UO, et al. (2020) Differences in micronutrient levels between urban and rural children in Cross River State, Nigeria. *New Zealand Journal of Medical Laboratory Science* **74**, 129–134.

188. Ugochukwu E, Onubogu C, Edokwe E, et al. (2014) Nutritional contents of lunch packs of primary school children in nnewi, Nigeria. *Ann Med Health Sci Res* **4**, 2014/09/04 ed., S108-14.

189. Ujunwa FA, Ikefuna AN, Nwokocha AR, et al. (2013) Hypertension and prehypertension among adolescents in secondary schools in Enugu, South East Nigeria. *Ital J Pediatr* **39**, 2013/11/05 ed., 70.

190. Umeokonkwo AA, Ibekwe MU, Umeokonkwo CD, et al. (2020) Nutritional status of school age children in Abakaliki metropolis, Ebonyi State, Nigeria. *BMC Pediatr* **20**, 2020/03/09 ed., 114.

191. Uwaezuoke SN, Okoli CV, Ubesie AC, et al. (2016) Primary hypertension among a population of Nigerian secondary school adolescents: prevalence and correlation with anthropometric indices: a cross-sectional study. *Niger J Clin Pract* **19**, 2016/08/20 ed., 649–54.

192. Wamba PC, Enyong Oben J & Cianflone K (2013) Prevalence of overweight, obesity, and thinness in Cameroon urban children and adolescents. *J Obes* **2013**, 2013/07/19 ed., 737592.

193. Ward KA, Cole TJ, Laskey MA, et al. (2014) The effect of prepubertal calcium carbonate supplementation on skeletal development in Gambian boys-a 12-year follow-up study. *J Clin Endocrinol Metab* **99**, 2014/04/26 ed., 3169–76.

**Supplementary Appendix C: Total number of studies reporting data for anthropometric indicators and anaemia in school-age children and adolescents, by country and region**

| **Region/country** | **Stunting** | | **Wasting** | | **Thinness** | | **Overweight and/or obesity** | | **Anaemia*** | |
| --- | --- | --- | --- | --- | --- | --- | --- | --- | --- | --- |
|  | **N (N multi-country)** | **N girls only (N multi-country)** | **N (N multi-country)** | **N girls only (N multi-country)** | **N (N multi-country)** | **N girls only (N multi-country)** | **N (N multi-country)** | **N girls only (N multi-country)** | **N (N multi-country)** | **N girls only (N multi-country)** |
| **West and Central Africa** |  |  |  |  |  |  |  |  |  |  |
| Benin | 3 |  |  |  | 2 (2) | (1) | (3) | (1) | (1) | (1) |
| Burkina Faso | 7 (1) |  | 1 |  | 5 (3) | (1) | 4 (4) | (1) | 5 (1) | (1) |
| Cameroon | 5 |  | 5 |  | 8 (1) | (1) | 4 (2) | (1) | 4 (1) | (1) |
| Cabo Verde |  |  |  |  |  |  | (1) |  |  |  |
| Chad |  |  |  |  | (1) | (1) | (2) | (1) |  |  |
| Côte d'Ivoire | 1 |  | 1 |  | 1 (1) | (1) | 1 (2) | (1) | 1 (1) | (1) |
| Democratic Republic of the Congo | 1 |  |  |  | 1 (1) | (1) | 2 (1) | (1) | 2 (1) | (1) |
| Gabon |  |  |  |  | (1) | (1) | (1) |  | (1) | (1) |
| Gambia | 2 |  |  |  | 2 (1) | (1) | 2 (2) | (1) | 1 (1) | (1) |
| Ghana | 8 (1) |  | 1 |  | 17 (4) | 1 (1) | 13 (6) | 1 (1) | 7 (1) | 1 (1) |
| Guinea |  |  |  |  | (1) | (1) | (2) | (1) | (1) | (1) |
| Guinea-Bissau | 2 |  |  |  | 2 |  | (1) |  | 1 |  |
| Liberia |  |  |  |  | (1) | (1) | (2) | (1) |  |  |
| Mali |  |  |  |  | (1) | (1) | 1 (2) | (1) | 1 (1) | (1) |
| Mauritania |  |  |  |  | (1) |  | (2) |  |  |  |
| Niger |  |  |  |  | (1) | (1) | (2) | (1) | (1) | (1) |
| Nigeria | 23 (1) |  | 10 |  | 18 (3) | 1 (1) | 54 (4) | 2 (1) | 6 |  |
| Sao Tome and Principe |  |  |  |  |  |  | (1) |  |  |  |
| Senegal | 2 |  |  |  | 1 (1) | (1) | 3 (3) | (1) | 1 (1) | (1) |
| Sierra Leone |  |  |  |  | (1) | (1) | (2) | (1) | (1) | (1) |
| Togo |  |  |  |  | (1) | (1) | 2 (1) | (1) | (1) | (1) |
| Multi-country | 1 |  |  |  | 4 |  | 6 |  | 1 |  |
| ***Region total*** | ***55*** | | ***18*** | | ***61*** | | ***92*** | | ***30*** | |
| **South Asia** |  |  |  |  |  |  |  |  |  |  |
| Afghanistan |  |  |  |  | 2 | 1 | 1 |  | 1 | 1 |
| Bangladesh | 3 |  |  |  | 15 (3) | 2 (2) | 14 (4) | 2 (2) | 2 (1) | 1 |
| Bhutan |  |  |  |  |  |  |  |  | 1 |  |
| India | 21 (2) | 4 | 2 |  | 51 (6) | 10 (3) | 63 (7) | 7 (3) | 26 (1) | 10 |
| Maldives |  |  |  |  |  |  |  |  |  |  |
| Nepal | 3 |  |  |  | 15 (2) | 3 (2) | 16 (3) | 3 (2) | 8 | 2 |
| Pakistan | 6 (2) | (1) | 1 |  | 10 | 1 | 15 | 1 | 5 | 1 |
| Sri Lanka | 5 (1) |  | 1 |  | 9 (1) |  | 9 (2) |  | 5 (1) |  |
| Multi-country | 2 | 1 |  |  | 6 | 2 | 6 | 2 | 1 |  |
| ***Region total*** | ***40*** | | ***4*** | | ***108*** | | ***124*** | | ***48*** | |
| **Eastern and Southern Africa** |  |  |  |  |  |  |  |  |  |  |
| Angola |  |  |  |  |  |  | 1 |  |  |  |
| Botswana |  |  |  |  |  |  |  |  |  |  |
| Burundi |  |  |  |  |  |  |  |  |  |  |
| Ethiopia | 18 (1*^¤^*) | 5 | 14 | 5 | 15 (1) | 1 | 10 (2) | 1 | 10 (2) | 3 |
| Kenya | 3 | 1 | 2 | 1 | 2 | 1 | 1 (2) |  | 2 (1) |  |
| Lesotho |  |  |  |  |  |  | 1 (1) |  |  |  |
| Madagascar | 1 |  | 1 |  | 1 |  |  |  |  |  |
| Malawi |  |  |  |  | 1 |  | (2) |  |  |  |
| Mozambique | 1 |  | 2 |  | 1 |  | 3 (1) | 2 | 2 | 2 |
| South Africa | 5 | 1 | 4 | 1 | 6 | 2 | 19 (3) | 3 | 4 (2) |  |
| Tanzania | 2 (1*^¤^*) |  | 2 |  | 2 (1) | 1 | 8 (3) |  | 1 |  |
| Uganda | 2 (1*^¤^*) |  | 2 |  | 1 (1) | 1 | 2 (3) | 1 | 3 | 1 |
| Zambia | 1 |  |  |  |  |  |  |  | 1 |  |
| Zimbabwe | 1 |  | 1 |  |  |  | 1 |  |  |  |
| Multi-country | 1 |  |  |  | 1 |  | 4 |  | 2 |  |
| ***Region total*** | ***35*** | | ***28*** | | ***30*** | | ***49*** | | ***25*** | |
| **Europe and Central Asia** |  |  |  |  |  |  |  |  |  |  |
| Albania |  |  |  |  |  |  | (2) |  |  |  |
| Azerbaijan |  |  |  |  |  |  | 1 (1) | 1 (15–49 years) | (1) | 1 (15–49 years) |
| Bosnia and Herzegovina |  |  |  |  |  |  | 1 (3) |  |  |  |
| Bulgaria |  |  |  |  |  |  | 1 (3) |  |  |  |
| Croatia |  |  |  |  |  |  | 4 (7) | 1 |  |  |
| Georgia |  |  |  |  |  |  | 1 (1) |  |  |  |
| Kazakhstan |  |  |  |  |  |  | 1 (1) |  | 3 (1) |  |
| Kosovo |  |  |  |  |  |  | 1 (1) |  |  |  |
| Macedonia | 2 |  |  |  | 2 |  | 4 (4) |  |  |  |
| Montenegro |  |  |  |  |  |  | 1 (1) |  |  |  |
| Romania |  |  |  |  | 2 |  | 5 (5) | (1) |  |  |
| Serbia | 1 |  | 1 |  | 1 |  | 6 (1) |  |  |  |
| Turkey | 5 |  | 2 |  | 17 |  | 44 (4) |  | 6 |  |
| Uzbekistan |  |  |  |  |  |  | (1) |  | (1) |  |
| Multi-country |  |  |  |  |  |  | 7 |  | 1 |  |
| ***Region total*** | ***8*** | | ***3*** | | ***22*** | | ***77*** | | ***10*** | |
| **Middle East and North Africa** |  |  |  |  |  |  |  |  |  |  |
| Algeria |  |  | 1 |  | 1 |  | 2 |  |  |  |
| Egypt |  |  |  |  | 1 |  | 3 |  |  |  |
| Iran | 1 |  | 1 |  | 2 |  | 4 |  | 2 |  |
| Iraq | 2 | 1 | 1 |  | 6 | 1 | 24 | 6 | 2 |  |
| Jordan |  |  |  |  | 1 |  | 1 (1) |  |  |  |
| Kuwait |  |  |  |  | 1 |  | 4 (1) | 1 |  |  |
| Lebanon | (1) |  | (1*^¤^*) |  | (1) |  | 1 (1) |  | (1) |  |
| Morocco | 1 |  |  |  |  |  | 5 |  | 1 |  |
| Oman |  |  |  |  |  |  | 4 |  |  |  |
| Qatar |  |  |  |  |  |  |  |  |  |  |
| Saudi Arabia |  |  |  |  | 1 |  | 1 |  |  |  |
| State of Palestine | 1 (1) |  | 1 (1*^¤^*) |  | 1 (1) |  | 10 (1) | 2 (1, boys only) | 1 (1) |  |
| Syrian Arab Republic | 2 (1) |  | (1*^¤^*) |  | 2 (1) |  | 4 (2) |  | 1 (1) |  |
| Tunisia |  |  |  |  |  |  | 1 |  |  |  |
| United Arab Emirates |  |  |  |  | 1 |  | 1 |  |  |  |
| Yemen |  |  |  |  |  |  | 2 |  |  |  |
| Multi-country | 1 |  | 1 |  | 1 |  | 2 |  | 1 |  |
| ***Region total*** | ***8*** | | ***5*** | | ***18*** | | ***69*** | | ***8*** | |
| **East Asia and the Pacific ^¥^** |  |  |  |  |  |  |  |  |  |  |
| Cambodia | 2 |  |  |  | 2 (2) | 1 | 2 (4) | 1 | 4 (1) | 1 |
| Fiji | (1) |  |  |  | 1 (3) | 1 | 3 (6) | 1 | (1) |  |
| Indonesia | 3 (1) | 1 |  |  | 7 (3) | 1 | 10 (5) |  | 7 (1) | 4 |
| Lao PDR |  |  |  |  | 1 (2) |  | 1 (4) |  | 1 (1) | 1 |
| Malaysia | 5 (1) |  |  |  | 14 (3) |  | 17 (5) |  | 3 (1) |  |
| Marshall Is. | 1 |  |  |  | 1 (1) |  | 1 (3) |  | (1) |  |
| Mongolia |  |  |  |  | (2) |  | (4) |  | (1) |  |
| Myanmar | 3 (1) | 1 |  |  | 4 (3) | 2 | 2 (5) | 1 | 2 (1) | 2 |
| Nauru | (1) |  |  |  | (3) |  | (4) |  |  |  |
| Papua New Guinea |  |  |  |  | (2) |  | (3) |  | (1) |  |
| Philippines | 1 (1) |  |  |  | 2 (3) |  | 2 (5) |  | 5 (1) |  |
| Samoa | (1) |  |  |  | 1 (3) |  | 1 (6) |  | 1 (1) |  |
| Thailand | 2 (1) | (1, boys only) |  |  | 3 (3) | (1, boys only) | 5 (4) |  | 2 (1) | (1, boys only) |
| Timor-Leste |  |  |  |  | 1 (2) |  | 1 (4) |  | 1 (1) |  |
| Tonga | (1) |  |  |  | (3) |  | 1 (6) |  | (1) |  |
| Vanuatu | 1 (1) |  |  |  | 2 (3) |  | 2 (6) |  | 1 (1) |  |
| Vietnam | 4 |  | 1 |  | 3 (2) |  | 9 (4) |  | 2 (1) |  |
| Multi-country | 1 |  |  |  | 3 |  | 7 |  | 1 |  |
| ***Region total*** | ***23*** | | ***1*** | | ***45*** | | ***63*** | | ***30*** | |
| **Latin America and the Caribbean** |  |  |  |  |  |  |  |  |  |  |
| Argentina |  |  |  |  |  |  | 1 |  | 1 |  |
| Barbados |  |  |  |  |  |  | 1 |  |  |  |
| Bolivia |  |  |  |  |  |  |  |  | 1 |  |
| Bonaire island |  |  |  |  |  |  | 1 |  | 1 |  |
| Brazil | 1 |  |  |  | 3 | 1 | 12 | 1 |  |  |
| Chile |  |  |  |  |  |  | 1 |  | 1 |  |
| Colombia | 3 |  |  |  | 2 |  | 3 |  |  |  |
| Costa Rica |  |  |  |  |  |  |  |  | 1 |  |
| Ecuador |  |  |  |  | 1 |  | 1 |  |  |  |
| Guatemala | 1 |  |  |  |  |  |  |  | 3 |  |
| Haiti | 3 |  |  |  | 2 |  | 2 |  | 4 |  |
| Mexico |  |  |  |  |  |  | 9 |  | 1 |  |
| Peru | 1 |  |  |  | 1 |  | 2 |  |  |  |
| Trinidad and Tobago |  |  |  |  |  |  | 1 |  |  |  |
| Turks and Caicos Islands |  |  |  |  | 1 |  | 1 |  |  |  |
| Uruguay |  |  |  |  |  |  |  |  | 1 |  |
| Venezuela | 1 |  | 1 |  | 1 |  | 4 |  |  |  |
| Multi-country |  |  |  |  |  |  |  |  |  |  |
| ***Region total*** | ***10*** | | ***1*** | | ***11*** | | ***39*** | | ***14*** | |

*Includes studies that also provide data for iron deficiency and/or iron deficiency anaemia; stunting, height-for-age <-2 standard deviations (SDs) below the WHO Child Growth Reference median; wasting, weight-for-height <-2 SD below the WHO Child Growth Reference median (90); thinness, BMI-for-age <-2 SDs below the WHO Growth Reference median (90); overweight, BMI-for-age >+1 SD above the WHO Growth Reference median OR BMI-for-age expressed as ≥85th percentile of the CDC growth reference OR BMI-for-age equivalent to BMI ≥25kg/m² using International Obesity Task Force (IOTF) cut-offs by sex; obese, BMI-for- age >+2 SD above the WHO Growth Reference median OR BMI-for-age expressed as ≥95th percentile of the CDC growth reference OR BMI-for-age equivalent to BMI ≥30kg/m² using IOTF cut-offs by sex (90–92); anaemia, children <12 years (haemoglobin, Hb, <115 g/L), children 12–14 years and females 15+ years (Hb <120 g/L), males 15+ years (Hb <130 g/L); iron deficiency, serum ferritin <15µg/L; iron-deficiency anaemia, various classifications used but involve a combination of anaemia and concurrent low ferritin level (iron deficiency + Hb <120 g/L) (93); N (N multi-country) indicates the total number of articles presenting data from single-country studies (and the number of multi-country articles in which the respective country is represented); ^¥^ indicates that study summary includes grey literature; ^¤^ indicates that the article presents pooled multi-country data; ; references cited as they appear in the reference list of the main article

**Supplementary Appendix D: Range in prevalence of anthropometric indicators, anaemia and/or iron deficiency in school-age children and adolescents, by country and region**

| **Region/country** | **Prevalence (% range)** | | | | | | | | | |
| --- | --- | --- | --- | --- | --- | --- | --- | --- | --- | --- |
|  | **Stunting** | **Wasting** | **Thinness** | **Overweight** | **Obesity** | **Overweight and obesity** | **Anaemia** | **Iron deficiency** | **Iron deficiency anaemia** |  |
| **West and Central Africa** |  |  |  |  |  |  |  |  |  |  |
| Benin | 21.8; 22.0 |  | 17.5; 39.8 | 8.3 (girls) | 0.6 | 11.2 | 57.4 (girls) |  |  |  |
| Burkina Faso | 8.8; 32.9 | 5.0 | 9.0; 15.1 | 5.9 (girls) | 0.6 (girls) | 2.1; 8.6 | 23.0; 40.4 |  |  |  |
| Cameroon | 5.3; 23.7 | 0.3; 5.5 | 0.6; 20.8 | 9.6; 12.4 | 1.9; 2.9 | 12.5; 17.8 | 5.0; 75.3 |  |  |  |
| Cabo Verde |  |  |  |  |  |  |  |  |  |  |
| Chad |  |  | 4.9 (girls) | 6.0 | 0.6 (girls) |  |  |  |  |  |
| Côte d'Ivoire | 61.0 |  | 13.0 | 8.0 | 1.0 |  | 41.6; 61.4 |  |  |  |
| Democratic Republic of the Congo | 5.8 | 26.8 | 15.5 | 12.8 (girls) | 5.0 |  | 53.9 (girls) | 33.9 | 30.0 |  |
| Gabon |  |  | 1.3 (girls) | 13.8 (girls) | 4.2 (girls) |  | 63.5 (girls) |  |  |  |
| Gambia | 9.1 |  | 19.8 | 9.2 (girls) | 2.1 (girls) | 13.3 | 35.0; 53.0 (boys), 29.0; 41.0 (girls) |  |  |  |
| Ghana | 3.2; 30.7 | 22.3 | 2.7; 25.7 | 3.2; 21.2 | 1.0; 11.5 | 6.0; 19.5 | 30.8; 73.0 | 4.0; 71.3 |  |  |
| Guinea |  |  | 2.3 (girls) | 10.2 (girls) | 1.2 (girls) |  | 47.1 (girls) |  |  |  |
| Guinea-Bissau | 6.4; 20.0 |  | 6.8 |  |  |  | 20.0; 42.0 |  |  |  |
| Liberia |  |  | 1.4 (girls) | 9.3 (girls) | 0.7 (girls) |  |  |  |  |  |
| Mali |  |  | 2.6 (girls) | 1.6 (boys), 3.3 (girls) | 0.9 (boys), 1.5 (girls) |  | 62.6; 63.2 |  |  |  |
| Mauritania |  |  | 22.3 |  | 3.4 | 24.3 |  |  |  |  |
| Niger |  |  | 6.3 (girls) | 4.8 (girls) | 0.9 (girls) |  | 46.0 (girls) |  |  |  |
| Nigeria | 0.4; 52.7 | 1.7; 10.9 | 5.6; 95.7 | 0.0; 16.4 | 0.0; 37.2 | 3.1 | 40.3; 85.5 |  |  |  |
| Sao Tome and Principe |  |  |  |  |  |  |  |  |  |  |
| Senegal | 2.0; 4.9 |  | 18.4 | 9.2; 11.0 | 1.4; 9.3 |  | 14.4 | 39.1 | 10.6 |  |
| Sierra Leone |  |  | 2.6 (girls) | 8.8 (girls) | 2.0 (girls) |  | 49.5 (girls) |  |  |  |
| Togo |  |  | 0.9 (girls) | 2.9; 5.2 | 1.7; 1.9 |  | 54.7 (girls) |  |  |  |
| Multi-country |  |  |  |  |  |  |  |  |  |  |
| ***Region total*** | **0.4; 61.0** | **0.3; 26.8** | **0.6; 95.7** | **0.0; 21.2** | **0.0; 37.2** | **2.1; 24.3** | **5.0; 85.5** | **4.0; 71.3** | **10.6; 30.0** |  |
| **South Asia** |  |  |  |  |  |  |  |  |  |  |
| Afghanistan |  |  | 9.5; 16.7* | 10.4; 25.3* | 2.0* |  | 29.9 (girls) |  |  |  |
| Bangladesh | 19.0; 36.8 |  | 26.7; 32.6* | 5.8; 19.2* | 1.3* |  | 17.1; 19.1* | 3.9; 9.5* | 1.3; 1.8* |  |
| Bhutan |  |  |  |  |  |  | 29.3* |  |  |  |
| India | 34.4* |  | 8.2; 44.9* | 4.9; 11.2* | 0.6; 5.2* |  | 23.4; 28.4* | 16.3; 50.0 | 16.0; 28.5 |  |
| Maldives |  | 6.9; 34.0 | 23.4* | 25.3* | 2.0* |  |  |  |  |  |
| Nepal | 32.7* |  | 7.2; 30.4* | 3.3; 14.7* | 0.4* |  | 31.0* | 3.7; 43.6 |  |  |
| Pakistan | 13.4* |  | 8.8; 28.8* | 3.0; 12.7* | 0.1; 1.3* |  | 37.1; 70.0 | 28.9; 41.5 |  |  |
| Sri Lanka | 30.4* | 30.0 | 25.1; 48.8* | 3.8; 8.5* | 0.4; 2.2* |  | 19.8* | 19.2* | 3.9; 12.4 |  |
| Multi-country |  | 12.0; 22.0 |  |  |  |  |  |  |  |  |
| ***Region total*** | **13.4*; 36.8** | **6.9; 34.0** | **7.2*; 48.8*** | **3.0*; 25.3*** | **0.1*; 5.2*** |  | **17.1; 70.0** | **3.7; 50.0** | **1.3; 28.5** |  |
| **Eastern and Southern Africa** |  |  |  |  |  |  |  |  |  |  |
| Angola |  |  |  | 17.7 |  |  |  |  |  |  |
| Botswana |  |  |  |  |  |  |  |  |  |  |
| Burundi |  |  |  |  |  |  |  |  |  |  |
| Ethiopia | 11.2; 57.0 | 7.1; 58.3 | 21.0; 27.1 | 4.4; 14.7 | 1.1; 13.3 |  | 10.7; 43.7 | 12.0 | 37.4 |  |
| Kenya | 27.0; 30.2 | 18.2 (boys), 15.6; 29.1 (girls) | 14.9 | 8.7; 14.4 | 3.1; 6.4 |  | 46.0; 53.0 | 29.0; 43.0 |  |  |
| Lesotho |  |  |  | 15.4 | 4.1 |  |  |  |  |  |
| Madagascar | 34.9 | 11.2 | 36.9 |  |  |  |  |  |  |  |
| Malawi |  |  | 24.4 (boys), 12.4 (girls) | 10.0 | 0.8 |  |  |  |  |  |
| Mozambique | 11.1 | 8.3; 12.0 |  | 12.6 (girls) |  | 11.4 | 42.4 | 32.7 |  |  |
| South Africa | 10.0; 37.0 | 18.3; 34.0 | 4.0; 81.0 | 4.7; 10.6 | 2.3; 3.4 |  | 13.8; 25.0 | 16.3; 27.7 | 6.0; 16.3 |  |
| Tanzania | 18.0; 30.7 | 4.5; 14.0 | 12.9 | 2.4; 25.0 | 4.5; 6.7 |  | 79.6 | 33.0 |  |  |
| Uganda | 6.6; 18.6 | 3.3; 10.1 | 5.8; 13.0 | 32.3 | 21.7 |  | 24.1; 46.0 |  |  |  |
| Zambia | 15.0 |  |  |  |  |  | 19.2 | 31.5 |  |  |
| Zimbabwe | 7.4 | 4.6 (boys), 3.6 (girls) |  |  | 11.4 |  |  |  |  |  |
| Multi-country |  |  |  |  |  |  |  |  |  |  |
| ***Region total*** | **6.6; 57.0** | **3.3; 58.3** | **4.0; 81.0** | **2.4; 32.3** | **0.8; 21.7** | **11.4** | **10.7; 53.0** | **12.0; 43.0** | **6.0; 37.4** |  |
| **Europe and Central Asia** |  |  |  |  |  |  |  |  |  |  |
| Albania |  |  |  |  |  | 28.2 (boys), 19.0 (girls) |  |  |  |  |
| Azerbaijan |  |  |  |  |  | 22.7 (boys), 24.7 (girls) | 6.3; 38.2 | 16.3; 34.1 | 23.8 |  |
| Bosnia and Herzegovina |  |  |  | 12.0 | 7.0 | 22.5 (boys), 17.7 (girls) |  |  |  |  |
| Bulgaria |  |  |  | 18.0 | 6.0 | 27.6 (boys), 23.2 (girls) |  |  |  |  |
| Croatia |  |  |  | 14.3 | 8.9 | 30.3 (boys), 20.9 (girls) |  |  |  |  |
| Georgia |  |  |  | 12.0 | 1.4 | 33.6 (boys), 20.8 (girls) |  |  |  |  |
| Kazakhstan |  |  |  | 2.3; 6.8 (boys), 3.2; 4.1 (girls) | 0.5; 0.9 (boys), 0.2; 0.8 (girls) | 17.4 (boys), 15.3 (girls) | 27.0; 32.4 | 13.0; 22.9 | 9.0 |  |
| Kosovo |  |  |  |  |  | 28.2 (boys), 18.9 (girls) |  |  |  |  |
| Macedonia | 2.0 |  | 2.0 | 1.5 | 3.2 | 29.3 (boys), 19.5 (girls) |  |  |  |  |
| Montenegro |  |  |  | 16.0 | 11.0 | 32.1 (boys), 22.9 (girls) |  |  |  |  |
| Romania |  |  | 5.4 | 6.0; 21.0 | 3.0; 11.8 | 22.9 (boys), 19.4 (girls) |  |  |  |  |
| Serbia | 20.0 | 4.0 | 8.0 | 17.0; 19.2 | 5.0; 6.9 | 22.7 (boys), 20.9 (girls) |  |  |  |  |
| Turkey | 4.4; 7.0 | 1.0 | 4.0; 26.0 | 1.5; 26.0 | 0.6; 26.0 | 28.0 (boys), 25.5 (girls) | 6.0; 41.0 | 19.0; 31.0 | 3.2; 4.0 |  |
| Uzbekistan |  |  |  |  |  |  | 17.0 |  |  |  |
| Multi-country |  |  |  |  |  |  |  |  |  |  |
| ***Region total*** | **2.0; 20.0** | **1.0; 4.0** | **2.0; 26.0** | **1.5; 26.0** | **0.6; 26.0** | **17.4; 33.6 (boys), 15.3; 25.5 (girls)** | **6.0; 41.0** | **13.0; 34.1** | **3.2; 23.8** |  |
| **Middle East and North Africa** |  |  |  |  |  |  |  |  |  |  |
| Algeria |  | 24.5 | 1.4; 8.8 | 22.8; 28.3 |  | 9.5 |  |  |  |  |
| Egypt |  |  | 8.6 (boys), 2.3 (girls) | 29.5 | 36.8 | 15.7; 28.9 (boys), 21.1; 30.7 (girls) |  |  |  |  |
| Iran | 34.2 | 0.9 | 3.4; 8.2 | 12.2 | 9.6; 14.9 | 21.8 | 26.0 | 9.5 |  |  |
| Iraq | 5.1 | 31.1 | 10.1; 23.5 | 7.5; 18.9 | 1.4; 12.5 | 5.2; 40.5 | 14.6 |  |  |  |
| Jordan |  |  | 12.2 |  |  | 38.8 |  |  |  |  |
| Kuwait |  |  | 11.0 | 24.4 (girls) | 8.9 (boys) | 30.0; 34.5 |  |  |  |  |
| Lebanon |  |  |  | 8.6 |  | 25.6; 50.5 (boys), 26.6; 46.5 (girls) | 18.3 | 14.2 |  |  |
| Morocco | 13.8 |  |  | 21.0; 34.8 | 7.5; 13.2 | 9.2; 30.2 | 18.3 |  |  |  |
| Oman |  |  |  | 5.1; 12.2 | 1.9; 3.6 |  |  |  |  |  |
| Qatar |  |  |  |  |  |  |  |  |  |  |
| Saudi Arabia |  |  | 8.6 (boys), 5.8 (girls) | 28.6 (boys), 18.9 (girls) | 7.9 (boys), 4.7 (girls) |  |  |  |  |  |
| State of Palestine | 3.4 | 5.1 | 1.9 | 12.2; 15.5 | 13.9; 27.0 | 23.1; 44.6 | 25.0 |  |  |  |
| Syrian Arab Republic | 7.0 |  | 3.0; 4.8 | 12.0; 24.3 | 6.0; 9.9 |  | 23.3; 30.0 |  |  |  |
| Tunisia |  |  |  | 18.9 | 8.6 | 19.7 (boys), 19.7 (girls) | 30.0 |  |  |  |
| United Arab Emirates |  |  | 4.8 | 17.4 (boys), 20.7 (girls) | 4.1 (boys), 4.4 (girls) |  |  |  |  |  |
| Yemen |  |  |  | 21.5 | 13.7 | 28.2 |  |  |  |  |
| Multi-country |  |  |  |  |  |  |  |  |  |  |
| ***Region total*** | **3.4; 34.2** | **0.9; 31.1** | **1.4; 23.5** | **5.1; 34.8** | **1.4; 36.8** | **5.2; 44.6** | **14.6; 30.0** | **9.5; 14.2** |  |  |
| **East Asia and the Pacific ^¥^** |  |  |  |  |  |  |  |  |  |  |
| Cambodia | 33.2*; 42.5 |  | 15.0 | 2.5 | 2.2 |  | 15.1; 49.4 |  |  |  |
| Fiji | 6.5 |  | 0.6; 25.9 | 13.9; 40.7 | 5.2; 14.7 | 19.2 |  |  |  |  |
| Indonesia | 23.8; 28.0 |  | 7.2*; 36.7 | 14.1; 28.9 | 7.9; 23.9 | 10.2; 32.6 | 14.0; 98.5 | 18.0 | 6.0; 13.5 |  |
| Lao PDR |  |  | 9.0 | 1.9 (boys), 6.6 (girls) | 8.4 (boys), 3.8 (girls) | 11.0; 14.0 | 42.5 |  |  |  |
| Malaysia | 3.5; 29.1 |  | 4.0; 22.0 | 10.9; 23.3 | 2.5; 26.5 | 23.8; 32.4 | 4.4 | 4.3 | 0.2 |  |
| Marshall Is. | 23.6 |  | 4.6 |  |  | 13.3 | 32.2 (boys),  24.6 (girls) |  |  |  |
| Mongolia |  |  | 2.0; 2.7 |  | 3.8; 7.1 (boys), 2.6; 4.7 (girls) | 10.1; 18.0 | 25.4 (boys),  23.3 (girls) |  |  |  |
| Myanmar | 20.6; 38.0 |  | 37.0 |  |  | 5.2 | 45.4; 59.1 (girls) |  | 35.5 |  |
| Nauru | 13.5 |  | 0.0; 1.0 |  |  | 44.5; 65.0 |  |  |  |  |
| Papua New Guinea |  |  | 1.0 |  |  | 32.0 | 24.5 (boys),  32.5 (girls) |  |  |  |
| Philippines | 20.3; 32.0* |  | 11.3; 30.0* | 9.0; 10.0 |  | 10.9 | 7.7; 12.5 |  |  |  |
| Samoa | 3.6 |  | 0.1; 1.0 | 32.6 | 19.3 | 51.7; 60.8 | 33.5 |  |  |  |
| Thailand | 7.4 |  | 7.4; 32.8 | 9.0 | 7.3 | 17.8 | 31.0 | 23.0 | 12.0 |  |
| Timor-Leste |  |  | 11; 21.8 |  |  | 1.7 | 19.5 (boys),  20.9 (girls) |  |  |  |
| Tonga | 5.7 |  | 0.2 | 13.9; 40.7 | 5.2; 21.1 | 19.2; 59.2 | 22.8 (boys),  26.4 (girls) |  |  |  |
| Vanuatu | 9.1 |  | 0.0; 7.1 | 11.5 | 0.4 | 0.0; 11.4 | 20.8 |  |  |  |
| Vietnam | 5.1; 25.4 | 5.3 | 14.0; 16.4 | 16.7; 32.2 | 5.1; 24.7 | 21.1; 22.1 | 11.4; 43.8 | 5.6; 6.3 | 0.4; 12.4 |  |
| Multi-country |  |  |  |  |  |  |  |  |  |  |
| ***Region total*** | **3.5; 42.5** | **5.3** | **0.0; 37.0** | **2.5; 40.7** | **0.4; 26.5** | **0.0; 59.2** | **4.4; 98.5** | **4.3; 23.0** | **0.2; 35.5** |  |
| **Latin America and the Caribbean** |  |  |  |  |  |  |  |  |  |  |
| Argentina |  |  |  | 23.0; 25.2 | 18.4; 18.5 |  | 4.4 |  |  |  |
| Barbados |  |  |  |  |  | 30.4 |  |  |  |  |
| Bolivia |  |  |  |  |  |  | 15.0; 21.5 |  |  |  |
| Bonaire island |  |  |  | 24.3 (boys), 31.9 (girls) | 9.9 (boys), 13.7 (girls) |  | 9.3 |  |  |  |
| Brazil | 1.7 |  | 2.1; 4.1 | 9.9; 73.0 | 3.4; 17.3 | 24.4; 42.0 |  |  |  |  |
| Chile |  |  |  |  | 18.5; 29.9 |  | 8.0; 11.0 |  |  |  |
| Colombia | 10.0; 11.3 |  | 4.4; 9.0 |  | 3.4; 23.8 | 17.6 |  |  |  |  |
| Costa Rica |  |  |  |  |  |  |  |  |  |  |
| Ecuador |  |  | 5.8 | 16.9 | 3.4 | 20.3 |  | 12.9; 13.3 |  |  |
| Guatemala | 14.8 |  |  |  |  |  | 51.1; 81.0 |  |  |  |
| Haiti | 12.2; 14.0 |  | 11.4; 14.5 | 5.1 | 1.8 |  | 3.9; 17.8 |  |  |  |
| Mexico |  |  |  | 16.0; 25.1 | 7.0; 28.1 | 43.0; 45.3 | 12.0 | 15.9 |  |  |
| Peru | 5.0 |  | <1.0 | 18.8; 28.0 | 22.7; 27.1 |  |  |  |  |  |
| Trinidad and Tobago |  |  |  |  |  | 23.0 |  |  |  |  |
| Turks and Caicos Islands |  |  | 9.1 | 17.2 | 23.6 |  |  |  |  |  |
| Uruguay |  |  |  |  |  |  | 47.4 |  |  |  |
| Venezuela | 24.0 | 9.0 | 10.0 | 9.5; 12.2 | 4.3; 37.2 | 14.1 |  |  |  |  |
| Multi-country |  |  |  |  |  |  |  |  |  |  |
| ***Region total*** | **1.7; 24.0** | **9.0** | **<1.0; 14.5** | **5.1; 73.0** | **1.8; 37.2** | **14.1; 45.3** | **3.9; 81.0** | **12.9; 15.9** |  |  |

Stunting, height-for-age <-2 standard deviations (SDs) below the WHO Child Growth Reference median; wasting, weight-for-height <-2 SD below the WHO Child Growth Reference median (90); thinness, BMI-for-age <-2 SDs below the WHO Growth Reference median (90); overweight, BMI-for-age >+1 SD above the WHO Growth Reference median OR BMI-for-age expressed as ≥85th percentile of the CDC growth reference OR BMI-for-age equivalent to BMI ≥25kg/m² using International Obesity Task Force (IOTF) cut-offs by sex; obese, BMI-for- age >+2 SD above the WHO Growth Reference median OR BMI-for-age expressed as ≥95th percentile of the CDC growth reference OR BMI-for-age equivalent to BMI ≥30kg/m² using IOTF cut-offs by sex (90–92); anaemia, children <12 years (haemoglobin, Hb, <115 g/L), children 12–14 years and females 15+ years (Hb <120 g/L), males 15+ years (Hb <130 g/L); iron deficiency, serum ferritin <15µg/L; iron-deficiency anaemia, various classifications used but involve a combination of anaemia and concurrent low ferritin level (iron deficiency + Hb <120 g/L) (93); prevalence range per region is based on the lowest and highest prevalence described across countries within the region for both sexes if available; references cited as they appear in the reference list of the main article
